# Design and Characterization of a Novel eEF2K Degrader with Potent Therapeutic Efficacy Against Triple‐Negative Breast Cancer

**DOI:** 10.1002/advs.202305035

**Published:** 2023-12-12

**Authors:** Changxin Zhong, Rongfeng Zhu, Ting Jiang, Sheng Tian, Xiaobao Zhao, Xiaoya Wan, Shilong Jiang, Zonglin Chen, Rong Gong, Linhao He, Jin‐Ming Yang, Na Ye, Yan Cheng

**Affiliations:** ^1^ Department of Pharmacy The Second Xiangya Hospital Central South University Changsha 410011 China; ^2^ Hunan Provincial Engineering Research Centre of Translational Medicine and Innovative Drug Changsha 410011 China; ^3^ Department of Medicinal Chemistry Jiangsu Key Laboratory of Neuropsychiatric Diseases and College of Pharmaceutical Sciences Soochow University Suzhou Jiangsu 215123 China; ^4^ Department of Pharmacy Xiangya Hospital Central South University Changsha 410011 China; ^5^ Department of General Surgery The Second Xiangya Hospital Central South University Changsha Hunan 410011 China; ^6^ Department of Cancer Biology and Toxicology Department of Pharmacology College of Medicine and Markey Cancer Center University of Kentucky Lexington KY 40536 USA; ^7^ Ministry of Education Key Laboratory of Diabetes Immunology (Central South University) Changsha 410011 China

**Keywords:** degrader, eEF2K, molecular glue, triple‐negative breast cancer

## Abstract

Dysregulated eEF2K expression is implicated in the pathogenesis of many human cancers, including triple‐negative breast cancer (TNBC), making it a plausible therapeutic target. However, specific eEF2K inhibitors with potent anti‐cancer activity have not been available so far. Targeted protein degradation has emerged as a new strategy for drug discovery. In this study, a novel small molecule chemical is designed and synthesized, named as compound **C1**, which shows potent activity in degrading eEF2K. **C1** selectively binds to F8, L10, R144, C146, E229, and Y236 of the eEF2K protein and promotes its proteasomal degradation by increasing the interaction between eEF2K and the ubiquitin E3 ligase βTRCP in the form of molecular glue. **C1** significantly inhibits the proliferation and metastasis of TNBC cells both in vitro and in vivo and in TNBC patient‐derived organoids, and these antitumor effects are attributed to the degradation of eEF2K by **C1**. Additionally, combination treatment of **C1** with paclitaxel, a commonly used chemotherapeutic drug, exhibits synergistic anti‐tumor effects against TNBC. This study not only generates a powerful research tool to investigate the therapeutic potential of targeting eEF2K, but also provides a promising lead compound for developing novel drugs for the treatment of TNBC and other cancers.

## Graphical abstract

Dysregulated eukaryotic elongation factor 2 kinase (eEF2K) expression is implicated in the pathogenesis of triple‐negative breast cancer (TNBC). Compound **C1** is designed and synthesized, which shows potent activity in degrading eEF2K protein. **C1** significantly inhibits the proliferation and metastasis of TNBC. These findings provide a promising therapeutic strategy for the development of novel therapeutic drugs for TNBC treatment.

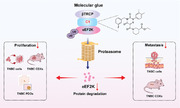

## 1 Introduction

Eukaryotic elongation factor 2 kinase (eEF2K), a member of the atypical protein kinase family termed alpha‐kinases and a regulator of protein synthesis,^[^ ^1^ ^]^ is highly overexpressed in breast, pancreatic, brain, and lung cancers, and is associated with poor survival of cancer patients.^[^ ^2^ ^]^ It was reported that deregulation of eEF2K is associated with unfavorable prognostic features and clinical outcomes of triple‐negative TNBC patients.^[^ ^3^ ^]^ We previously demonstrated that targeting eEF2K inhibited tumor progression,^[^ ^4^ ^]^ and enhanced the efficacy of cancer immunotherapy,^[^ ^5^ ^]^ suggesting that eEF2K could be exploited as a therapeutic target for cancer. However, few selective and potent inhibitors of eEF2K have been identified thus far due to weak (e.g., A‐484954) or non‐selective inhibition of eEF2K (e.g., NH125).^[^ ^6^ ^]^ Therefore, identification of highly effective and specific small molecules targeting eEF2K may help develop novel therapeutic agents against cancer.^[^ ^7^ ^]^

Targeted protein degradation (TPD) is an approach that fundamentally removes the protein through endogenous protein degradation mechanisms rather than inhibiting it.^[^ ^8^ ^]^ TPD offers many advantages over inhibition strategies, including removal of target proteins and consequent ablation of all associated functions, providing an opportunity to treat diseases driven by undruggable proteins.^[^ ^9^ ^]^ TPD, which includes proteolysis‐targeting chimeras (PROTAC) and molecular glue degraders,^[^ ^10^ ^]^ has shifted drug discovery from functional inhibitors to proteolytic degraders of targeted proteins through activation of the ubiquitin‐proteasome system. PROTACs are heterobifunctional degraders rationally designed by connecting a target protein ligand and an E3 ligase recruiter via a linker. In contrast, molecular glues act as monovalent inducers to reinforce or induce protein–protein interactions (PPI) between ubiquitin ligase and the target protein (substrate),^[^ ^11^ ^]^ which are mostly identified serendipitously from phenotypic screening.^[^ ^9^, ^12^ ^]^ Phenotypic screening as an alternative to target‐based screening represents a powerful and efficient approach to oncology drug discovery in recent decades.^[^ ^13^ ^]^ Pharmacologically active molecules identified from phenotypic assays (e.g., viability and cytotoxicity) with unprecedented drug mechanisms may demonstrate greater potential to be translated into therapeutically relevant efficacy than those from target‐based assays.^[^ ^13b^ ^]^

In the current study, we developed the compound **C1** as a novel small‐molecule degrader of eEF2K through a scaffold‐hopping design strategy and cell‐based phenotypic screening. The Compound **C1** acted as a molecular glue to enhance the interaction of eEF2K with the ubiquitin E3 ligase βTRCP, thereby promoting the proteasomal degradation of the kinase. Remarkably, compound **C1** exhibited potent anti‐cancer activity in the cell culture, mouse xenograft models and patient‐derived tumor organoids of TNBC, which highly express eEF2K; and the combination of **C1** and paclitaxel, a commonly used chemotherapeutic drug, showed a synergistic effect against TNBC both in vitro and in vivo. These results indicate that pharmacologic manipulation of eEF2K protein degradation may represent an innovative and potential targeted therapy for TNBC and other cancers.

## 2 Results

### 2.1 Design, Synthesis, and Biological Evaluation of Compound C1 as a Degrader of eEF2K

**Figure 1:**
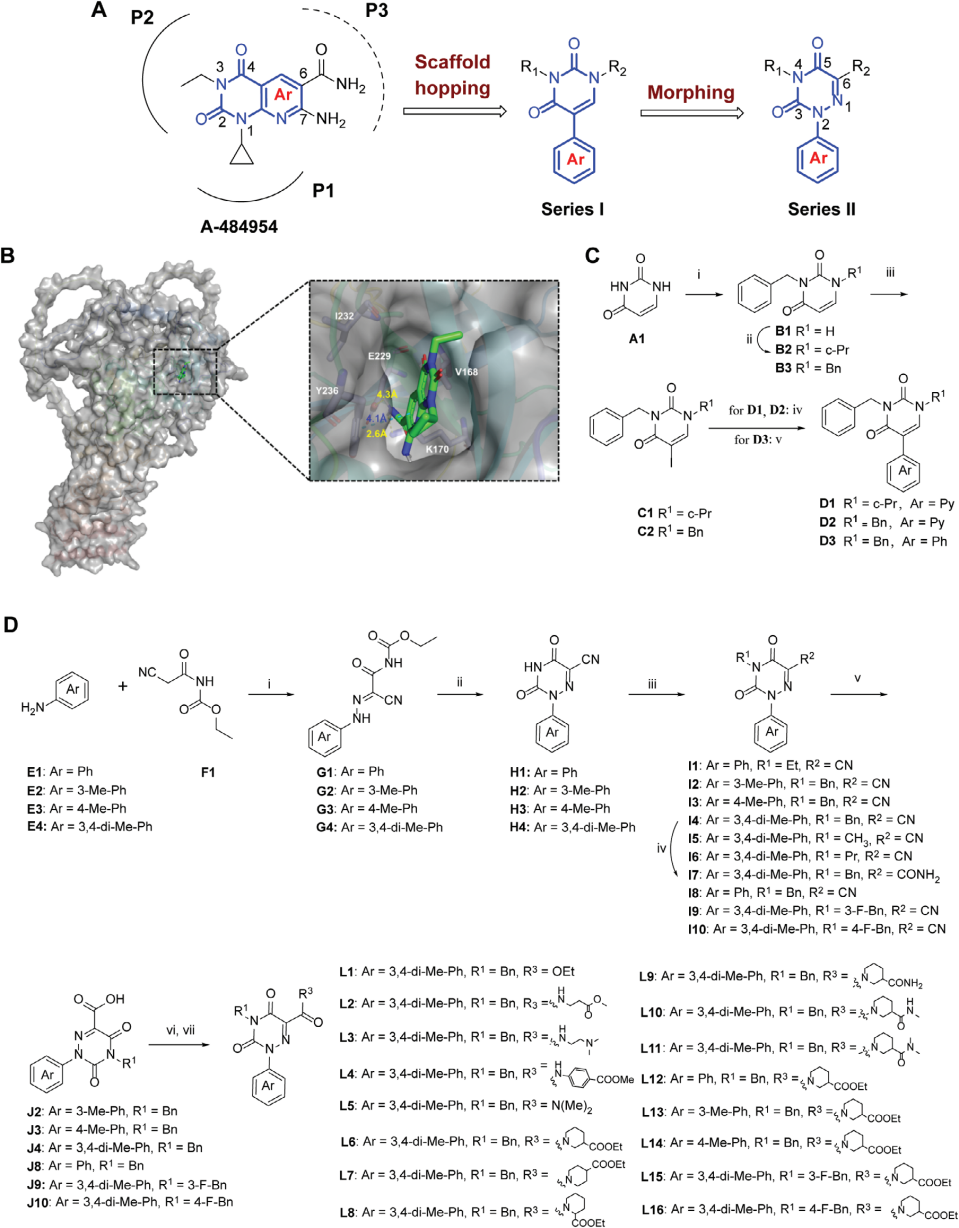
Development of eEF2K modulators. A) Design of new eEF2K modulators based on A‐484954. B) A‐484954 docked into the ATP‐binding pocket of eEF2K (PDB code: 7shq). Docking determined the three key residues (Y236, K170, and E229) that are shown in sticks. Hydrogen bonds are shown as yellow dashed lines, and *π*–*π* stacking is shown as a blue dashed line. C) The chemical synthesis of series I compounds. Reagents and conditions (i) K_2_CO_3_, acetonitrile, benzyl bromide, r.t, 12 h, 20–40%. (ii) Cyclopropyl boronic acid, 2,2′‐dipyridyl, Cu(OAc)_2_, dichloroethane, 80 °C, 12 h, 60–70%. (iii) NIS, AcOH, N_2_, r.t, 12 h, 65–75%. (iv) (2‐Pyridinyl)tributylstannane, Pd(PPh_3_)_2_Cl_2_, THF, r.t, 12 h, 50–65%. (v) Phenylboronic acid, Na_2_CO_3_, (Ph_3_)_4_P, acetonitrile, water, 80 °C, overnight, 40–50%. D) The chemical synthesis of series II compounds. Reagents and conditions: (i) NaNO_2_, H_2_O, pyridine, AcONa, 0–5 °C, 90–95%. (ii) AcONa, AcOH, 110 °C, 4 h, 80–90%. (iii) K_2_CO_3_, substituted benzyl bromide, benzyl bromide, or CH_3_I, acetonitrile, r.t, 75–85%. (iv) Ethanol, DMSO, H_2_O_2_, NaOH, 40–60%. (v) Concentrated HCl, AcOH, 110 °C, 70–80%. (vi) Oxaloyl chloride, N_2_, DCM, DMF, rt, 85–95%. (vii) Et_3_N, DCM, substituted amine, 0 °C to r.t, 60–70%.

Starting from A‐484954, a well‐recognized selective eEF2K inhibitor, we first investigated its binding mode to the protein structure of eEF2K predicted by AlphaFold due to the lack of available eEF2K full‐length crystal structure. As shown in **Figure** **1**A,B, A‐484954 binds to the kinase active site of the eEF2K protein, which is consistent with the most recent crystallized CaM•*pe*EF2K_TR_ complex bound to A‐484954.^[^ ^14^ ^]^ The pyrido[2,3‐d]pyrimidine‐2,4(1*H*,3*H*)‐dione core of A‐484954 generates a *π*–*π* stacking interaction with Y236. Its 1‐cyclopropyl and 3‐ethyl groups (P1 and P2) form two hydrophobic interactions, whereas the 6‐amide moiety (P3) forms key hydrogen bonds with residues K170 and E229. We thus designed, synthesized, and evaluated several 5‐arylpyrimidine‐2,4(1*H*,3*H*)‐dione derivatives (Series I, **D2**‐**D4**) through unfolding the fused bicyclic core of A‐484954 by using a “scaffold hopping” strategy (Figure 1A,C). Unfortunately, none of the series I compounds showed cytotoxicity against MDA‐MB‐231 cells with high expression of eEF2K.

Subsequent morphing of series I led to the discovery of 2‐aryl‐1,2,4‐triazine‐3,5(2*H*,4*H*)‐dione derivatives (Series II, Figure 1A,D) furnishing diverse hydrophilic moieties (R_2_) binding to the hydrogen‐bonding region P3. A comparison of the potency of cyano compounds **I1‐I6** indicates that the benzyl group (R_1_) and 3.4‐dimethyl substituent on the Ar moiety are important structural elements for achieving strong anti‐cancer activity, thus identifying **I4** as the most potent compound in this subseries with an IC_50_ value of 950.6 nm (Table S1, Supporting Information) against MDA‐MB‐231 cells and an Avg K_D_ value of 692 nm binding to eEF2K protein in the SPR assay (Table S2, Supporting Information). Later, the cyano group of **I4** was converted to primary amide (**I7**), acid (**J4**), ethyl ester (**L1**), secondary amides (**L2**‐**L4**), and tertiary amides (**L5**‐**L8**). The structure‐activity relationship (SAR) revealed that the substituent (R_2_) at the 2‐position was sensitive to structural modification. Only tertiary amides **L5**‐**L8** showed comparable or even better anti‐cancer activity against MDA‐MB‐231 cells than **I4** (Table S1, Supporting Information). Ethyl piperidine‐3‐carboxylate **L6**, with an IC_50_ value of 110.9 nm, is ≈8‐fold more potent than its 2‐carboxylate (**L7**) and 4‐carboxylate (**L8**) analogs. Further ammonolysis of ethyl ester **L6** dramatically decreased in cellular activity (as in **L9‐L11**). Likewise, removal of the 3‐ or/and 4‐methyl substituent on the Ar moiety of **L6** dramatically decreased cellular activity (as in **L12‐L14**), suggesting the significant contribution of these two methyl groups to improve anti‐cancer activity. Considering that fluorine is commonly used in drug design and development for improving protein–ligand interactions and membrane permeability,^[^ ^15^ ^]^ **L15**‐**L16** were thus designed by the introduction of a fluorine atom at the 3‐ and 4‐position of the benzyl group (R_1_) of **L6**, thereby leading to the identification of 3‐fluorobenzyl derivative **L15** with comparable cellular activity (IC_50_: 131.2 vs 110.9 nm) and the strongest binding affinity with the eEF2K protein in the SPR assay (Avg *K*_D_: 1.10 nm vs 822 nm, Tables S1 and S2, Supporting Information). Hence, ethyl 1‐(2‐(3,4‐dimethylphenyl)−4‐(3‐fluorobenzyl)−3,5‐dioxo‐2,3,4,5‐tetrahydro‐1,2,4‐triazine‐6‐carbonyl)piperidine‐3‐carboxylate (**L15**, also named as compound **C1**), as the most potent eEF2K modulator, was selected to further investigate its mode of action and potential anti‐cancer activity.

**Figure 2:**
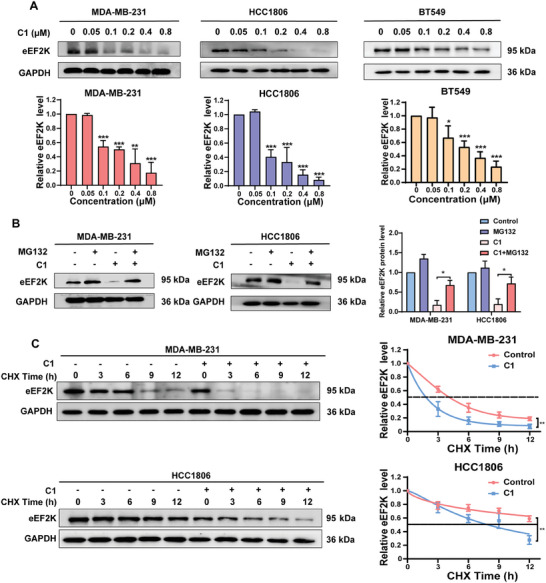
**C1** promotes the degradation of eEF2K. A) Western blotting was used to detect the expression of eEF2K in MDA‐MB‐231, HCC1806, and BT549 cells treated with **C1** at 0, 0.05, 0.1, 0.2, 0.4, and 0.8 µm for 48 h. GAPDH was used as a loading control. Relative eEF2K protein levels were shown as the means ± SDs, ^*^ *p* < 0.05, ^**^ *p* < 0.01, ^***^ *p* < 0.001, *t*‐test. B) MDA‐MB‐231 and HCC1806 cells were stimulated with 0.2 µm **C1** for 48 h with or without 20 µm MG132, and eEF2K levels were assessed by western blotting. GAPDH was used as a loading control. C) MDA‐MB‐231 and HCC1806 cells were stimulated with 0.2 µm **C1** for 48 h in the presence or absence of 20 µg mL^−1^ CHX. eEF2K levels were assessed by western blotting. GAPDH was used as a loading control.

We determined the effect of **C1** on eEF2K protein expression and found that **C1** decreased the level of eEF2K protein in a dose‐dependent manner in TNBC cells (**Figure** **2**A). However, **C1** had no significant effect on eEF2K mRNA levels (Figure S1, Supporting Information). MG132, a proteasome inhibitor, blocked the down regulation of eEF2K induced by **C1** (Figure 2B). Furthermore, the CHX chase assay showed that the turnover rate of eEF2K was significantly accelerated in **C1**‐treated TNBC cells (Figure 2C). These results suggest that **C1** down‐regulates eEF2K by promoting its proteasomal degradation.

### 2.2 C1 Increases the Interaction Between eEF2K and Ubiquitin E3 Ligase βTRCP

The SPR assay showed that the binding between **C1** and eEF2K became strong in a concentration‐dependent manner (**Figure** **3**A). To further validate that **C1** binds with eEF2K, a cellular thermal shift assay showed that eEF2K had higher thermal stability after **C1** treatment (Figure 3B). Furthermore, we developed a biotinylated probe, **C1**‐biotin (Figure S2, Supporting Information), and found that **C1**‐biotin could specifically pull down eEF2K (Figure 3C). These results supported the direct binding between **C1** and the eEF2K protein.

Figure 3
**C1** enhances the interaction between eEF2K and the ubiquitin E3 ligase βTRCP. A) The binding curves of various concentrations of **C1** with eEF2K were examined by SPR. B) TNBC cells were treated with or without **C1** followed by a cellular thermal shift assay (CETSA). Error bars represent means ± SDs of triplicate experiments. C) Biotin pull‐down assay using eEF2K overexpressed HEK‐293T cells. D‐biotin was used as a negative control. SA, streptavidin. D) The binding mode of **C1** to the homology model of eEF2K developed by AlphaFold. eEF2K protein is shown in cartoon representation. **C1** is labeled in color by atoms. The hydrogen bonds and halogen bonds are labeled as dashed lines. The key amino acid residues for the binding are labeled as carbon. E) Biotin pull‐down assay using wild‐type or site‐mutated eEF2K plasmid in HEK‐293T cells. F) HEK‐293T cells transfected with eEF2K wild type or mutant plasmids were treated with or without **C1** followed by a cellular thermal shift assay (CETSA). Error bars represent means ± SDs of triplicate experiments. G) MDA‐MB‐231 and HCC1806 cells were treated with 0.2 µm **C1** for 48 h in the presence or absence of 10 µm MLN4924, and eEF2K levels were assessed by western blotting. GAPDH was used as a loading control. H) HEK‐293T cells co‐transfected with Flag‐eEF2K and Myc‐Ub, were treated with 0.2 or 0.4 µm **C1** in the presence of 20 µm MG132 for 4 h to accumulate polyubiquitylated proteins. I) HEK‐293T cells were transfected with Flag‐eEF2K and HA‐βTRCP, followed by **C1** treatment, co‐immunoprecipitation was used to detect interaction between Flag‐eEF2K and HA‐βTRCP. J) Computational analysis of the binding mode of **C1** with βTRCP and eEF2K protein by using Glide docking of the Schrodinger 9.0. K) The binding free energy of βTRCP and eEF2K was analyzed by MM/GBSA calculations. L) The binding free energy of βTRCP and eEF2K in the presence of **C1** was analyzed by MM/GBSA calculations.

**Figure:**
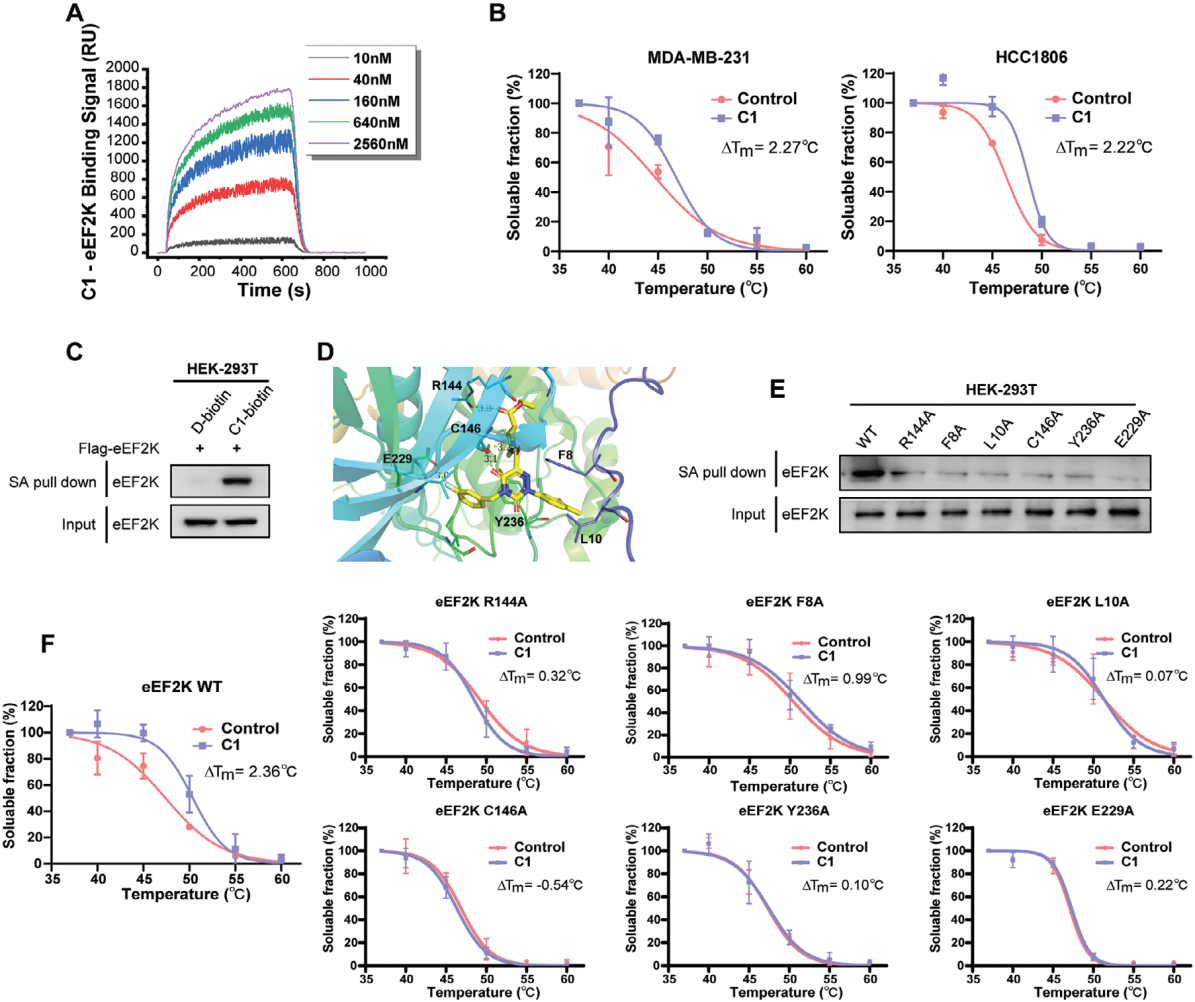

**Figure:**
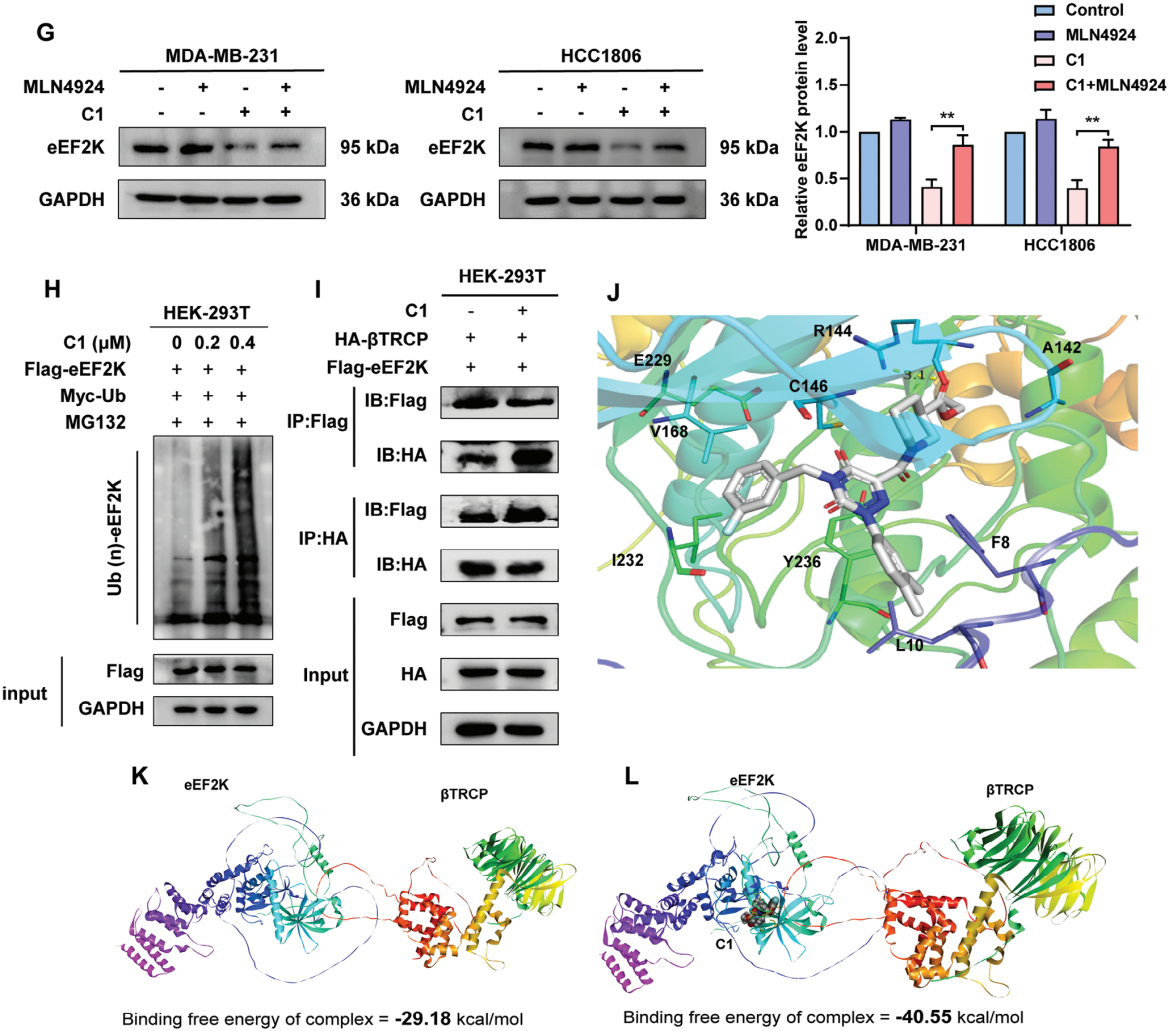

We next investigated the binding mode of **C1** to the protein structure of eEF2K predicted by AlphaFold. The molecular docking results showed that **C1** occupied the active pocket of the eEF2K protein, including residues F8, L10, R144, C146, E229, and Y236 (Figure 3D). The nonplanar 2‐phenyl‐1,2,4‐triazine‐3,5(2*H*,4*H*)‐dione scaffold of **C1** binds to eEF2K via aromatic face‐to‐face *π*–*π* stacking interactions with residues Y236 and F8, and the 3,4‐dimethyl group on the Ar group forms van der Waals interactions with L10. Moreover, key hydrogen bonds were observed between its dicarbonyl and ester moieties with residues C146 and R144, respectively. In addition, the fluorine atom of **C1** is potentially able to interact with E229 via a halogen bond, indicating the important role of R_1_ groups. Subsequently, the replacement of R144, F8, L10, C146, Y236, or E229 with alanine was found to prevent the binding of **C1** with eEF2K (Figure 3E), supporting the proposed binding mode from computer modeling. Consistently, a cellular thermal shift assay indicated that mutation of amino acids R144, F8, L10, C146, Y236, and E229 impaired the thermal stability of the eEF2K protein in the presence of **C1**, indicating that the interactions between eEF2K and **C1** were disrupted (Figure 3F). These data highlight the requirement of F8, L10, R144, C146, E229, and Y236 for the stable interaction with **C1**. Together, these results exhibit a positive correlation between the aforementioned SAR study, eEF2K binding affinity, molecular docking study, and point mutation assays, demonstrating the key structural features for achieving potent degradation of eEF2K.

Next, we treated TNBC cells with the E1 ubiquitin‐activating enzyme inhibitor MLN4924. As shown in Figure 3G, the down regulation of eEF2K induced by **C1** was remarkedly reversed by MLN4924 treatment, suggesting that **C1** induced the degradation of eEF2K via the ubiquitin‐proteasome pathway. We further showed that **C1** increased the polyubiquitylation of eEF2K (Figure 3H). It has been reported that βTRCP, an E3 ubiquitin ligase, mediates the degradation of eEF2K.^[^ ^16^ ^]^ Herein, we found that **C1** enhanced the binding of βTRCP to eEF2K (Figure 3I). We then constructed a binding model of the **C1**‐eEF2K‐βTRCP ternary complex (Figure 3J), and found that the predicted binding free energy between eEF2K and βTRCP was changed from −29.18 to −40.55 kcal mol^−1^ owing to the involvement of **C1**, suggesting that **C1** enhanced the binding stability of eEF2K and βTRCP (Figure 3K,L). These results indicate that **C1** acts as a molecular glue to promote the interaction of βTRCP and eEF2K, thus triggering degradation of eEF2K.

### 2.3 In Vitro Effects of C1 on TNBC Cells

**Figure 4:**
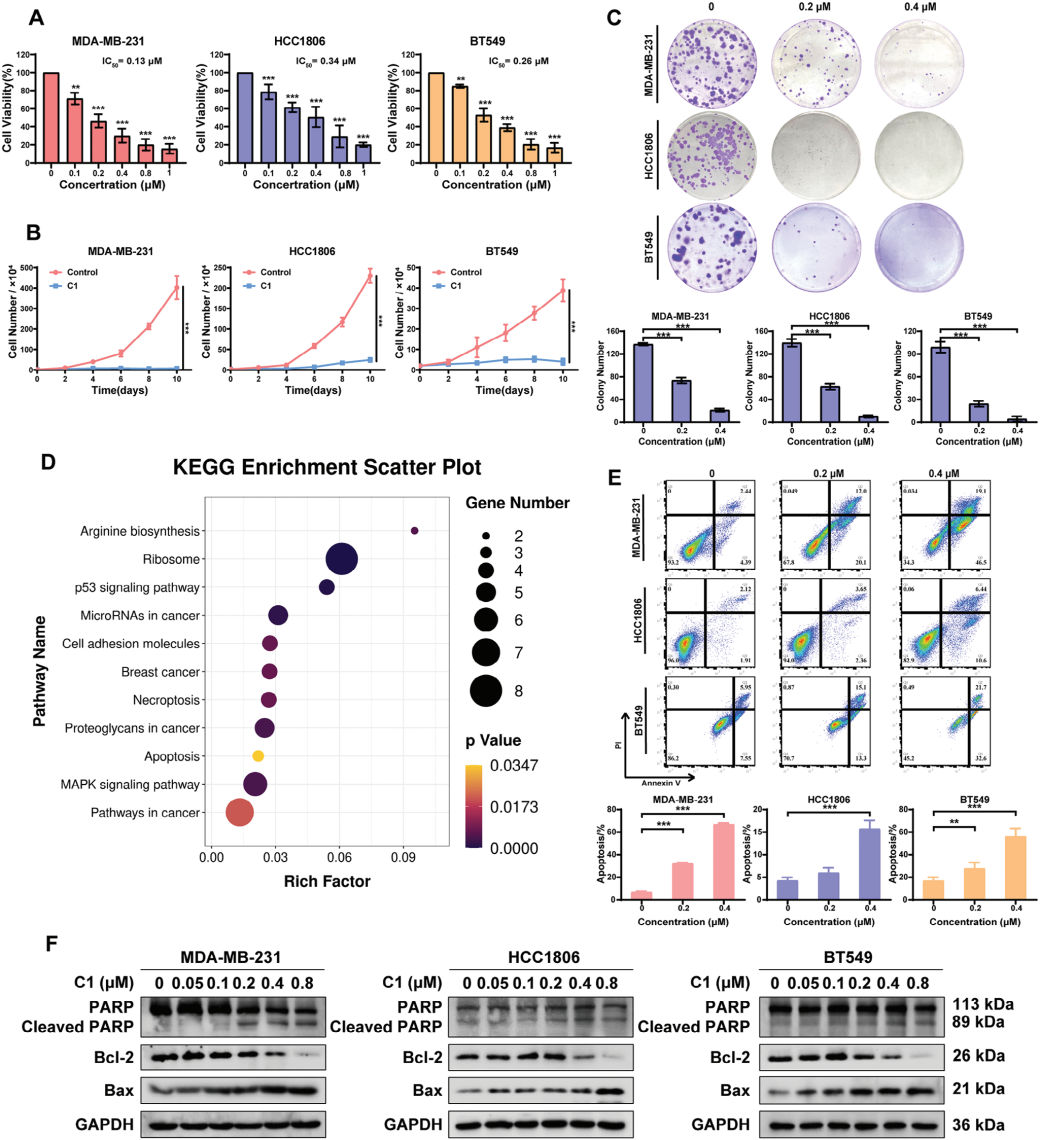
**C1** inhibits cell proliferation and induces apoptosis of TNBC cells. A) MDA‐MB‐231, HCC1806 and BT549 cells were treated with **C1** at different concentrations for 72 h followed by CCK‐8 assay. The IC_50_ was shown. The results are displayed as means ± SDs (*n* = 3). ^**^ *p* < 0.01, ^***^ *p* < 0.001, *t*‐test. B) TNBC cells were treated with 0.2 µm **C1** for indicated durations. Cell proliferation was analyzed by cell counting. ^**^ *p* < 0.01, ^***^ *p* < 0.001, *t*‐test. C) Colony‐formation assay of MDA‐MB‐231, HCC1806 and BT549 cells grown for 12 days in the presence of DMSO and **C1** (0.2 or 0.4 µm). The results are displayed as means ± SDs (*n* =  3). ^**^ *p* < 0.01, ^***^ *p* < 0.001, *t*‐test. D) KEGG enriched pathway analysis of the global proteomic profiling data in the **C1** treatment groups compared with the DMSO groups in MDA‐MB‐231 cells. E) The apoptosis of MDA‐MB‐231, HCC1806, and BT549 cells was determined by flow cytometry after treatment with DMSO and **C1** (0.2 or 0.4 µm) for 48 h. The apoptosis rates are displayed as means ± SDs (*n* = 3). ^**^ *p* < 0.01, ^***^ *p* < 0.001, *t*‐test. F) The apoptosis markers such as cleaved PARP, Bcl‐2 and Bax were analyzed by western blotting. GAPDH was used as a loading control.

To evaluate the functional consequence of eEF2K degradation caused by **C1**, we examined the effects of **C1** on TNBC cells. **Figure** **4**A shows that **C1** decreased the cell viability of MDA‐MB‐231, HCC1806, and BT549 cells in a dose‐dependent manner, with IC_50_ values of 0.131, 0.342, and 0.262 µm, respectively. The cell growth curve and colony formation assay showed that **C1** significantly inhibited cell proliferation of those TNBC cells (Figure 4B,C). Additionally, we performed the global proteomic profiling and found that eEF2K‐related signaling pathways, including ribosome and apoptosis, were enriched in **C1**‐treated MDA‐MB‐231 cells based on KEGG pathway analysis, indicating that the biological mechanism of **C1** depends on its regulation of eEF2K (Figure 4D). Activation of apoptosis by **C1** was confirmed by increased Annexin V/PI staining, cleaved PARP, and Bax, and decreased Bcl‐2 (Figure 4E,F).

**Figure 5:**
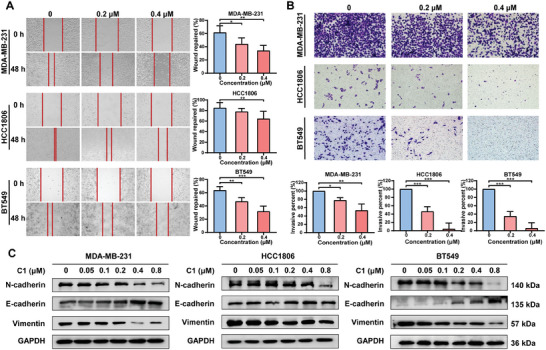
**C1** inhibits TNBC cell migration and invasion. A) The migration of MDA‐MB‐231, HCC1806, and BT549 cells after **C1** treatment was measured by wound‐healing assay. The results were displayed as the means ± SDs (*n* = 3), ^**^ *p* < 0.01; ^***^ *p* < 0.001, *t*‐test. B) The invasion of MDA‐MB‐231, HCC1806, and BT549 cells subjected to **C1** was examined by transwell assay. The results were presented as the means ± SDs (*n* = 3), ^*^ *p* < 0.05, ^**^ *p* < 0.01, ^***^ *p* < 0.001, *t*‐test. C) Western blotting analysis of E‐cadherin, *N*‐cadherin, and Vimentin in MDA‐MB‐231, HCC1806, and BT549 cells treated with **C1**.

The expression of eEF2K has been reported to be causally associated with tumor cell invasion and metastasis^[^ ^3^, ^15^ ^]^ and mediates epithelial‐to‐mesenchymal transition (EMT).^[^ ^18^ ^]^ We showed that **C1** treatment remarkably inhibited the cell migration and invasion of TNBC cells in wound healing and transwell assays (**Figure** **5**A,B) and led to an increase in the expression of E‐cadherin and a decrease in the expression of N‐cadherin and Vimentin (Figure 5C).

**Figure 6:**
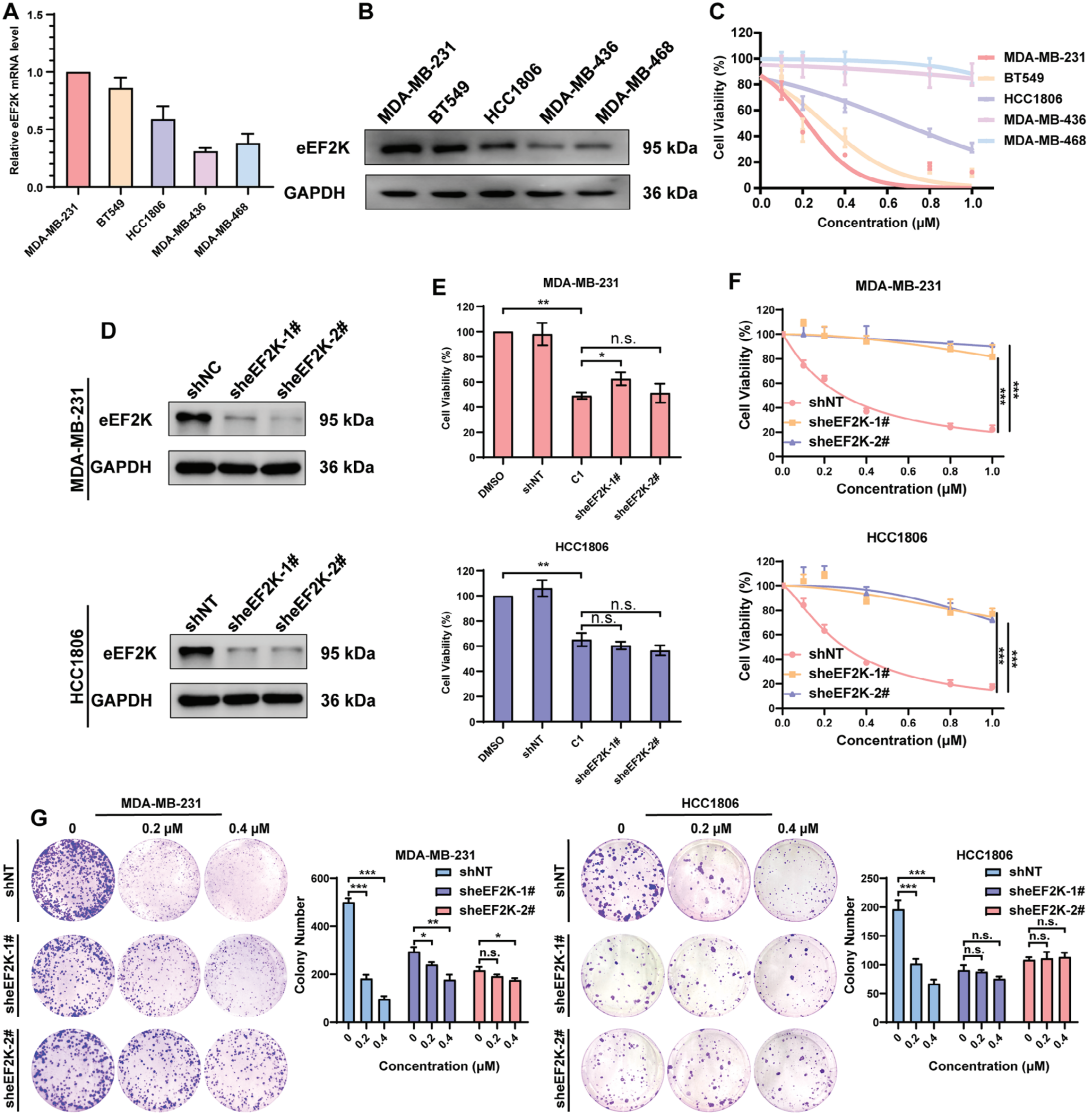
Inhibitory effects of **C1** on TNBC cells are dependent on eEF2K expression. A) The relative eEF2K mRNA expression in different TNBC cell lines was determined by qRT‐PCR. B) The expressions of eEF2K protein in different TNBC cell lines was determined by western blotting. GAPDH was used as a loading control. C) Anti‐proliferation effects of **C1** in different TNBC cell lines. D) Western blotting was employed to examine eEF2K expression in non‐target (shNT) or stable eEF2K knockdown (sheEF2K 1#, 2#) MDA‐MB‐231, and HCC1806 cells. E) Effects of C1 and sheEF2K on proliferation of MDA‐MB‐231 and HCC1806 cells. F) CCK‐8 assay for the cell viability of control or eEF2K‐KD cells treated with **C1**. The results are displayed as means ± SDs (*n* = 3). ^***^ *p* < 0.001, *t*‐test. G) Colony‐formation assay for proliferation of control and eEF2K‐KD cells treated with **C1**. The results are displayed as means ± SDs (*n* = 3). ^*^ *p* < 0.05, ^**^ *p* < 0.01, ^***^ *p* < 0.001. n.s., no significant, *t*‐test.

Furthermore, we assessed the relationship between the anti‐cancer activity and eEF2K expression in tumor cells treated with **C1**. We found that TNBC cells with high eEF2K expression were more sensitive to **C1** treatment than cells with low eEF2K expression, indicating that the inhibitory effect of **C1** on cell viability was positively correlated with the expression of eEF2K in TNBC cells (**Figure** **6**A–C). Furthermore, we constructed stable eEF2K knockdown TNBC cells (Figure 6D) and found that **C1** had comparable effects on cell survival as knockdown of eEF2K in MDA‐MB‐231 and HCC1806 cells (Figure 6E). MDA‐MB‐231 and HCC1806 cells subjected to stable knockdown of eEF2K were insensitive to **C1** compared to control cells (Figure 6F,G). These results support the concept that the anti‐tumor activity of **C1** results from its promoting effect on eEF2K degradation.

### 2.4 In Vivo Effects of C1 on TNBC

**Figure 7:**
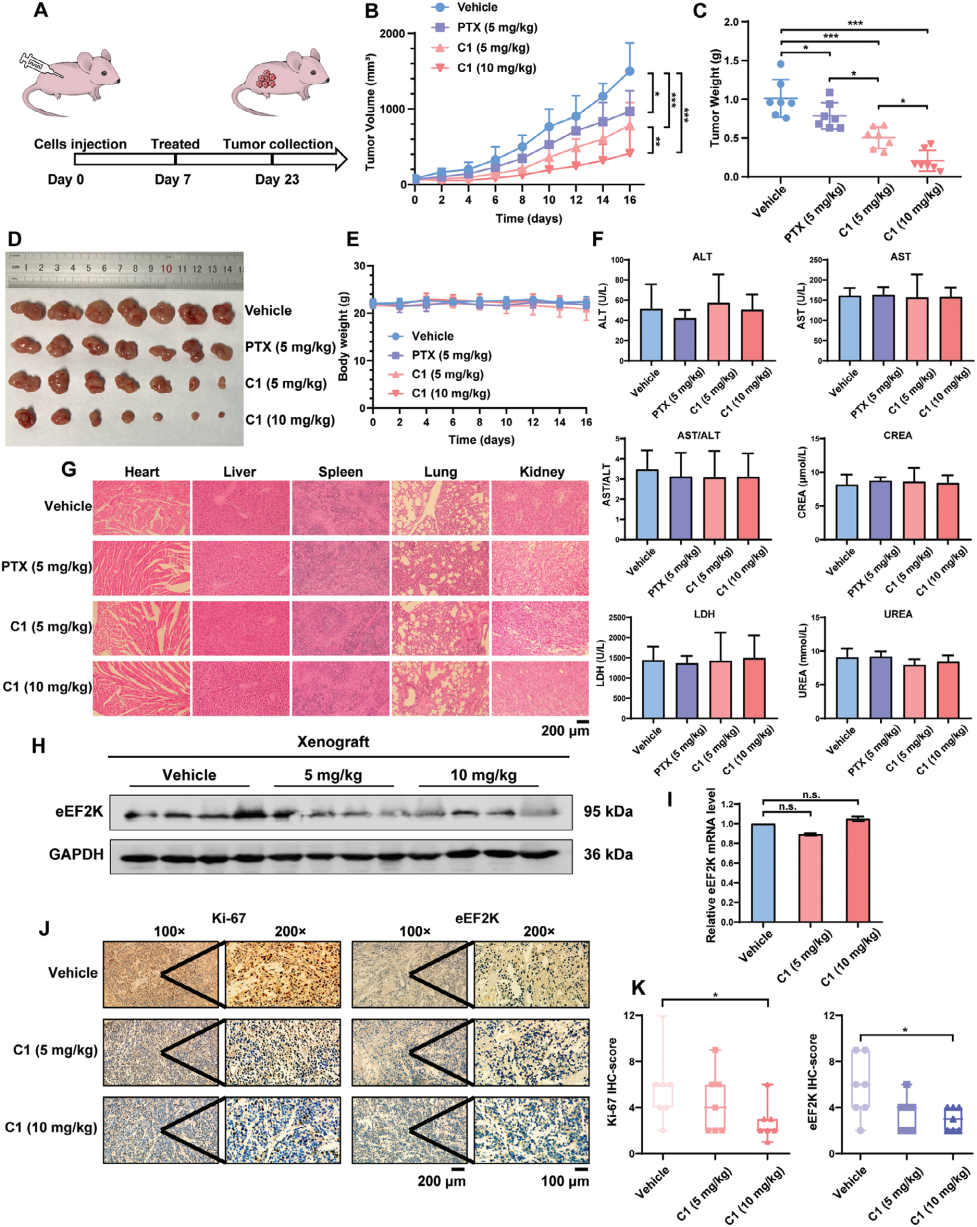
Effects of **C1** on TNBC growth in mouse xenograft models. A) Schematic view of the xenografted model. B) Mice bearing MDA‐MB‐231 xenografts were administered with vehicle (DMSO), paclitaxel (5 mg kg^−1^) and **C1** (5 or 10 mg kg^−1^), and tumor volumes were measured every other day (*n* = 7). Tumor growth curve was shown. C) The weight of xenograft tumors. D) Picture of the resected xenografts. E) Body weights of mice were shown. F) Serum biochemical parameters for kidney, liver, and blood toxicity were measured for each animal treated with vehicle control, paclitaxel or **C1**. G) Organs were subjected to hematoxylin and eosin (H&E) staining, and representative images are shown. H) eEF2K levels in tumors were measured by western blotting. GAPDH was used as a loading control. I) Relative eEF2K mRNA levels in tumors were measured by qRT‐PCR. Error bars represent means ± SDs of triplicate experiments. n.s., no significant, *t*‐test. J,K) Representative IHC staining of eEF2K and Ki‐67, the statistical results are displayed as means ± SDs (*n* = 7). ^*^ *p* < 0.05.

To recapitulate the in vivo effects of **C1**, we established a mouse model of TNBC using MDA‐MB‐231 cells (**Figure** **7**A). As shown in Figure 7B–D, the tumor volumes and weights were significantly decreased in mice bearing MDA‐MB‐231 xenograft tumors subjected to **C1** treatment compared to the control group receiving vehicle. Notably, **C1** showed stronger anti‐cancer activity than paclitaxel, a standard chemotherapeutic agent commonly used in the treatment of TNBC. There was no significant change in the body weights of mice treated with **C1** (Figure 7E). We tested a series of serum biochemical markers for hepatorenal toxicity in the animals and observed no overt drug‐related toxicity at therapeutic doses of **C1** (Figure 7F). In addition, H&E staining showed that there was no significant pathological injury in vital organs such as the heart, liver, spleen, lung, and kidney (Figure 7G). These data suggest that **C1** is effective and safe in the treatment of cancer.

eEF2K expression in the MDA‐MB‐231 xenograft tumors treated with **C1** showed a marked down‐regulation (Figure 7H). **C1** did not affect eEF2K mRNA levels in tumors, as analyzed by qRT‐PCR (Figure 7I). Immunohistochemical analysis showed that the expression levels of Ki‐67 and eEF2K were significantly reduced by **C1** treatment compared with the vehicle treatment (Figure 7J,K). Taken together, these results reveal that **C1** down‐regulates eEF2K and exerts a potent tumor suppressive effect in vivo.

**Figure 8:**
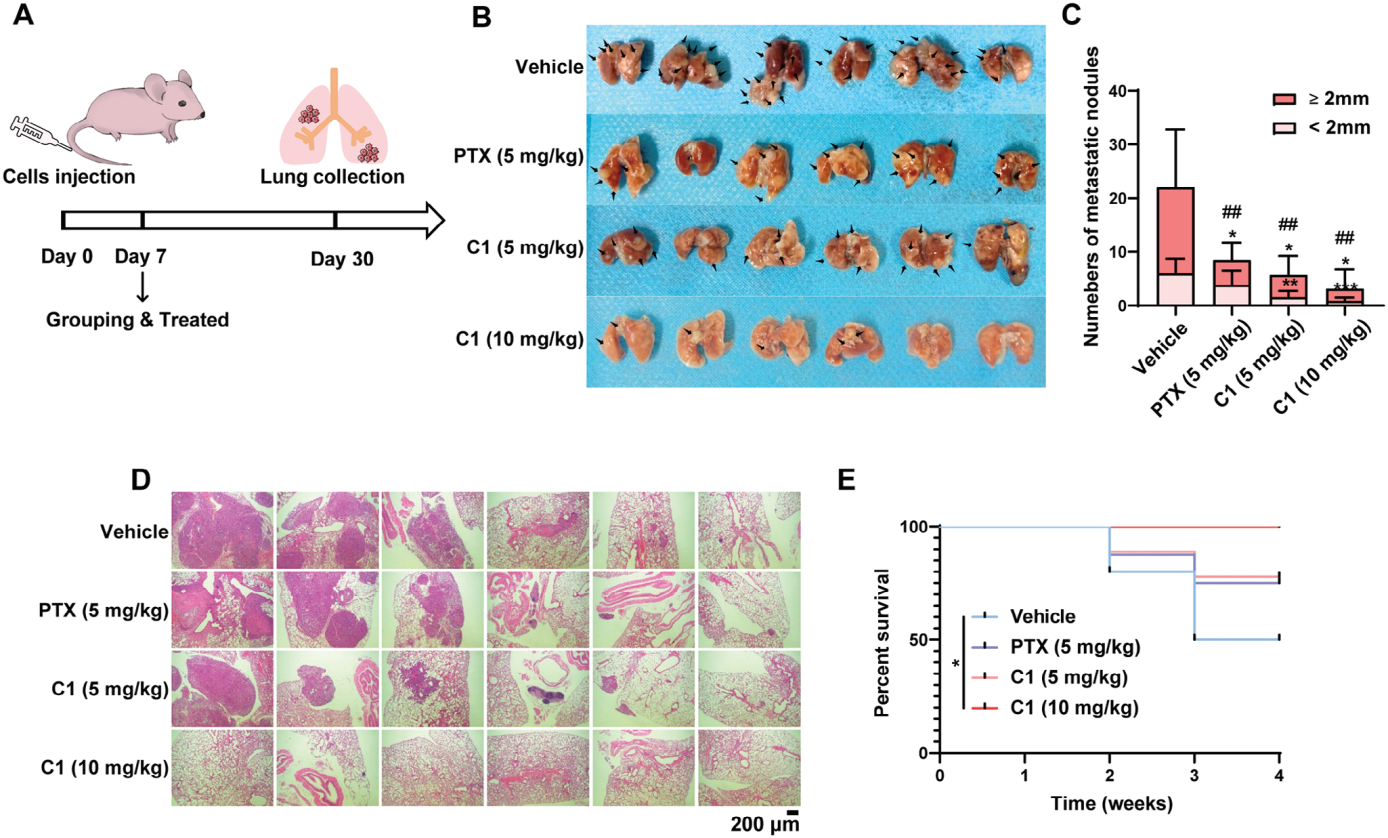
Effect of **C1** on TNBC metastasis in mouse xenograft models. A) Schematic view of the xenografted model. B,C) Tumor nodules formed on the surface of lung from different groups of the MDA‐MB‐231 tumor model. Black arrows indicate tumor nodules. The results were disaplayed as the means ± SDs (*n* = 6), *p < 0.05, **p < 0.01, ***p < 0.001, ^##^ *p* < 0.01, *t*‐test. D) Histological H&E staining performed on lung metastasis samples from the triple‐negative cancer models. E) Overall survival of mice with the indicated treatment.

**C1** also suppressed cell metastasis in the in vivo model (**Figure** **8**A). As shown in Figure 8B,C, **C1** at 5 mg kg^−1^ significantly reduced the number of lung nodules in the mice transplanted with MDA‐MB‐231 cells, while **C1** at 10 mg kg^−1^ almost completely prevented tumor lung metastasis. The histological analysis demonstrated that **C1** blocked the metastasis of TNBC cells into lungs (Figure 8D). Furthermore, it was observed that the survival duration of mice treated with **C1** was markedly prolonged compared with that of the mice treated with vehicle (Figure 8E).

### 2.5 C1 Exerts Significant Anti‐Cancer Activity in TNBC Patient‐Derived Organoids

**Figure 9:**
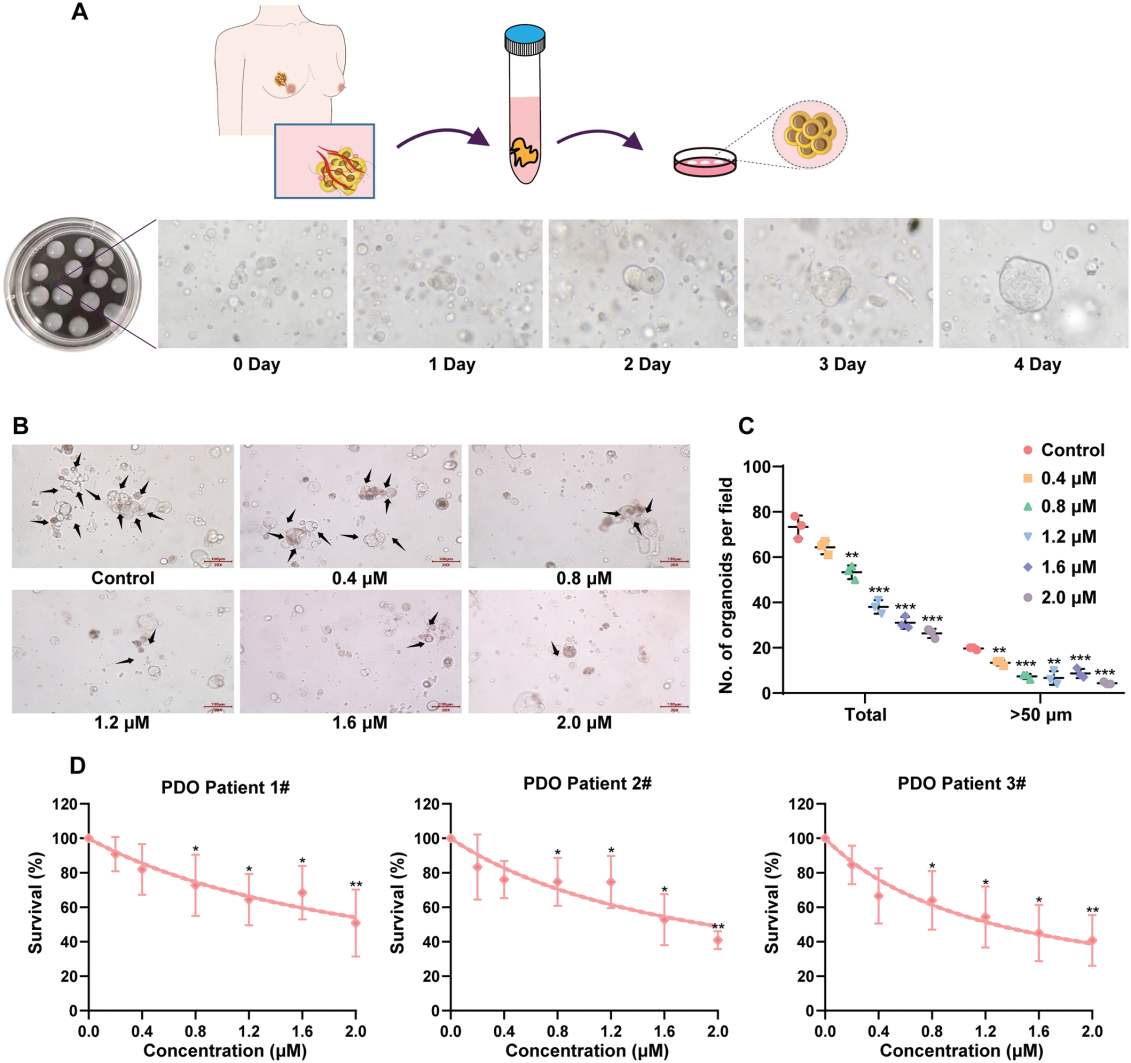
Efficacy of **C1** in Patient‐derived models of TNBCs. A) Schematic view of the TNBC patient‐derived organoid model. B) Representative images of the organoids treated with **C1**. Black arrows indicate survival organoids. C) Statistics of tumor organoid size and relative number of formed organoids. Error bars represent means ± SDs of triplicate experiments. ^**^ *p* < 0.01, ^***^ *p* < 0.001, *t*‐test. D) The proliferation curve of TNBC PDOs treated with vehicle and increasing concentrations of **C1** for 72 h. Error bars represent means ± SDs of triplicate experiments. ^*^ *p* < 0.05, ^**^ *p* < 0.01, ^***^ *p* < 0.001, *t*‐test.

Using the clinical specimens of TNBC, we built a patient‐derived organoid (PDO) model to further test the antitumor activity of **C1**. The tumor cells from the specimens of three TNBC patients were cultured in a 3D system to generate tumor organoids (**Figure** **9**A). As shown in Figure 9B,C, **C1** treatment resulted in a significant inhibition of the growth of TNBC organoids, as evidenced by the reduced size and number of organoids. The growth inhibition curve of TNBC patient‐derived tumor organoids demonstrated the potent inhibitory effect of **C1** on TNBC progression (Figure 9D), further supporting the therapeutic potential of **C1** in the treatment of TNBC.

### 2.6 Combination Therapy with C1 and Paclitaxel Exhibits Synergistic Anti‐Cancer Effects

**Figure 10:**
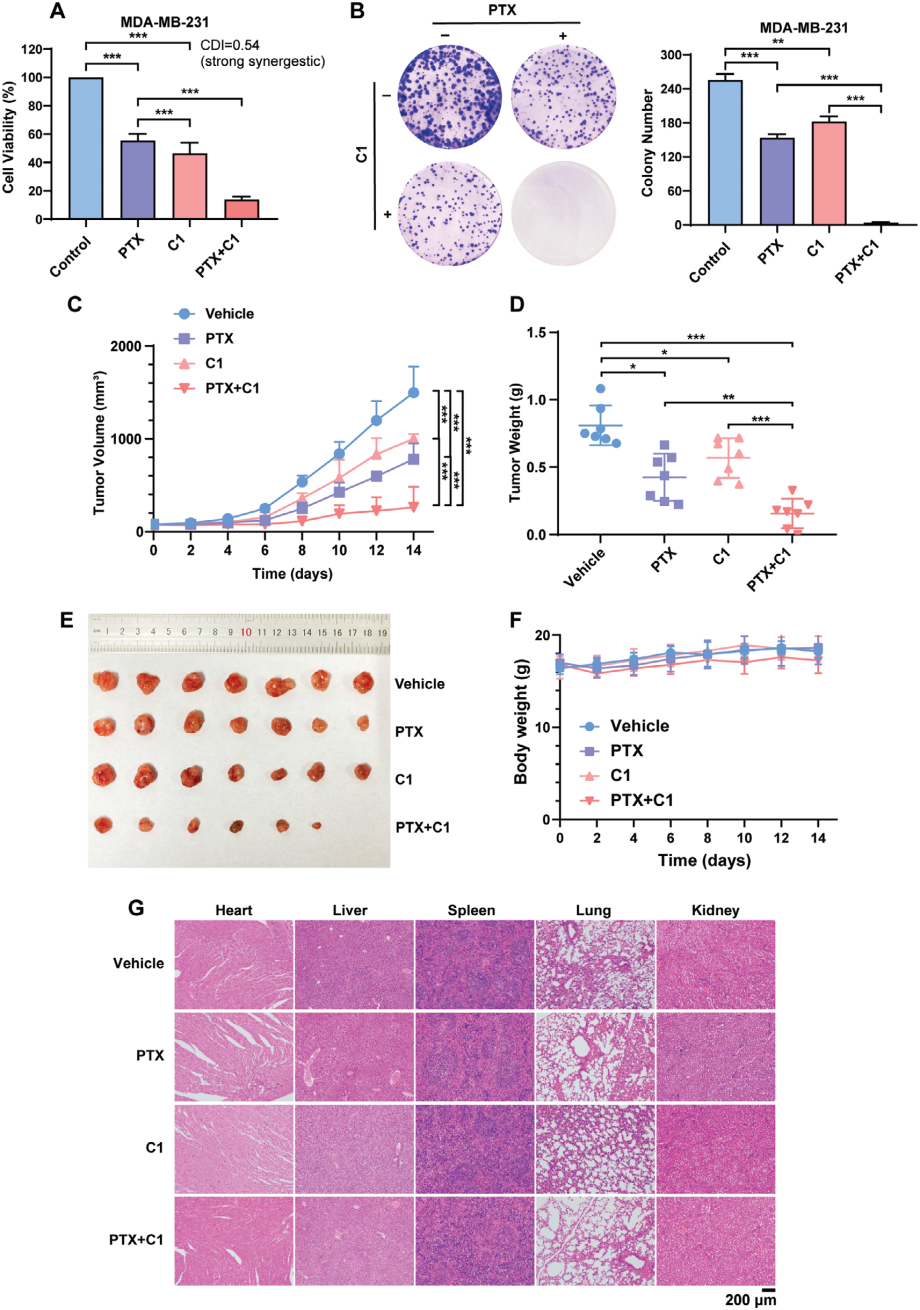
Combination treatment of **C1** with paclitaxel synergistically inhibits the growth of TNBC cells in vitro and in vivo. A) MDA‐MB‐231 cells were treated with PTX (50 nm) or **C1** (0.2 µm) alone or in combination, followed by CCK‐8 assay. CDI value was shown. B) Colony formation of MDA‐MB‐231 cells with PTX or **C1** alone or in combination. Representative images of wells are shown. C) Mice bearing MDA‐MB‐231 xenografts were administered with DMSO, PTX (10 mg kg^−1^), **C1** (5 mg kg^−1^) and PTX plus **C1**, and tumor volumes were measured every other day (*n* = 7). The tumor growth curve was shown. D) The weight of tumors. E) Picture of the resected xenografted tumors. F) Body weights of mice were shown. G) Organs were subjected to hematoxylin and eosin (H&E) staining, and representative images are shown.

Previous studies have shown the potential therapeutic utility of eEF2K in sensitizing cancer cells to paclitaxel. Here, we tested whether the combined use of **C1** with PTX can produce stronger anti‐cancer effects than **C1** or PTX alone. **Figure** **10**A shows that this combination treatment caused a significantly greater reduction in cell viability than **C1** or PTX alone, and the coefficient of drug interaction value (CDI) was 0.54, indicating that **C1** synergizes with PTX in suppressing tumor cell growth. **C1** in combination with PTX also exhibited synergistic inhibition of cell proliferation, as demonstrated by the dramatically attenuated colony formation (Figure 10B). Consistent with the in vitro observations, **C1** significantly potentiated the antitumor effect of PTX in vivo (Figure 10C–E). There was no measurable toxicity in the animals receiving the combined treatment, as evidenced by no observed changes in the body weight (Figure 10F) and no abnormalities in heart, liver, spleen, lung, and kidney histology (Figure 10G).

## 3 Discussion

Increasing evidence supports eEF2K as a promising therapeutic target for cancer including TNBC. However, due to the lack of a 3D structure of eEF2K, it is difficult to find an accurate binding domain to design or screen small molecule inhibitors for this kinase.^[^ ^1a^ ^]^ To date, only one study has reported the functional role of eEF2K PROTACs, but it showed limited eEF2K degradation and anti‐cancer effects.^[^ ^19^ ^]^ The concept of “molecular glue” was first introduced in the early 1990s,^[^ ^20^ ^]^ and since then, it has been considered as a small molecular degrader that mainly induces or stabilizes PPIs between an E3 ligase and a target protein to form a ternary complex, leading to protein ubiquitination and subsequent proteasomal degradation.^[^ ^11^ ^]^ The serendipitous discovery of molecular glues offered researchers solutions to unmarketable target proteins. In recent years, an increasing number of inhibitors have been found to degrade target proteins via molecular glues mechanisms and play a role in cancer treatment including CDK inhibitors,^[^ ^21^ ^]^ which offer an alternative approach to innovative drug discovery through targeted protein degradation.

In this study, we designed compound **C1** and demonstrated that this compound acts as a molecular glue to enhance the binding between eEF2K and βTRCP, thus promoting the ubiquitylation and degradation of eEF2K. Further study to determine the crystal structure of eEF2K and the accurate binding mode of **C1** and eEF2K may help us better understand the functional effect of **C1** as a molecular glue of eEF2K and facilitate the development of potent and selective eEF2K degraders.

The substantial down regulation of eEF2K by **C1** prompted us to test the antitumor activity of this compound. We show that **C1** exhibited a strong inhibitory effect on TNBC cell proliferation and metastasis both in vitro and in vivo. Importantly, **C1** exerted potent anti‐cancer effects in patient‐derived organoids, suggesting promising clinical application prospects. Combination therapy has been proven to be an effective way of treating cancers in the clinic. Paclitaxel (PTX) is a cancer chemotherapeutic agent of the taxane family;^[^ ^22^ ^]^ however, the clinical usage of PTX is limited due to intrinsic and acquired resistance.^[^ ^23^ ^]^ Thus, a significant effort is currently being directed toward improving its efficacy by combining it with different agents.^[^ ^24^ ^]^ We demonstrated here that co‐treatment of **C1** and PTC exhibited a synergistic anti‐cancer effect against TNBC, providing a novel combination treatment to sensitize the efficacy of PTX against TNBC. **C1** therefore might serve as a great candidate agent for further exploring eEF2K function in both physiological and pathological processes and provide a therapeutic avenue for treating cancers that are dependent on eEF2K.

Taken together, we report here that **C1**, the first molecular glue of eEF2K, possesses strong anti‐cancer activity in vitro, in vivo, and in TNBC organoids, and elicits a synergistic tumor inhibitory effect with PTX. Our study demonstrates the potential of new small‐molecule degraders of eEF2K as anticancer agents for the treatment of TNBC and other types of cancer.

## 4 Experimental Section

### Cell Culture

HCC1806 and HEK‐293T cells were derived from the Cancer Institute, Central South University. MDA‐MB‐231 and BT549 cells were purchased from Cell Bank (Chinese Academy of Sciences, Beijing, China). All cultured cells were supplemented with 10% fetal bovine serum and 1% penicillin‐streptomycin solution, and incubated at 37 °C in humid air with 5% CO_2_. Cell lines were authenticated using STR profile analysis and used within 3–20 passages of thawing the original stocks.

### Surface Plasmon Resonance (SPR) Assay

The chemically modified label‐free photocross‐linker sensor chips were provided by Betterways Inc. For array spotting, a BioDot AD‐1520 Array Printer (BIODOT Inc., USA) was employed to print samples and controls on the surface. Each candidate compound was printed with a volume of 1.875 nL per dot using the BioDot 1520 array printer to form five nonadjacent repetitions of each sample on the same chip. Then, the flow cell chambers were assembled onto the chip surface, and the chips were inserted and tested in the SPR instrument one after another. The solvents for chemical compounds (DMSO) and for proteins (PBS, pH 7.0) were crosswise tested individually as blank controls and background noise controls. To monitor the enrichment process of the target protein, a real‐time surface plasmon resonance experiment was performed using the bScreen LB 991 Label‐free Microarray System. During the SPR test, the chip surface was first primed three times with HBS‐EP running buffer at a rate of 2 µL s^−1^ for 40 s and one time with running buffer (1 × PBS with 5% DMSO) at a rate of 2 µL s^−1^ for 40 s. The mouse ELP2 protein was diluted separately with running buffer at concentrations of 200, 400, 800, 1600, and 3200 nm and injected over 600 s at a flow rate of 0.5 µL s^−1^ in each associating stage, followed by running buffer for 360 s at a flow rate of 0.5 µL s^−1^ in each dissociating stage. At the end of each associating–dissociating circle, the surface was regenerated to remove any remaining bound material with a pulse of 10 mm glycine‐HCl (pH 2.5) at 20 µL min^−1^ for 30 s.

### Biotin‐Binding Protein Pull‐Down

Flag‐eEF2K and Myc‐eEF2K mutant plasmid DNA was incubated with Lipofectamine 8000 (Invitrogen) in DMEM and added to HEK‐293T cells. Then, HEK‐293T cell lysates were divided into two equal volumes, added Flag or Myc antibody for 1 h at 4 °C, and incubated with **C1**‐biotin overnight at 4 °C, followed by pull‐down with streptavidin magnetic beads. The proteins bound to streptavidin magnetic beads were separated by SDS‐PAGE.

### Cellular Thermal Shift Assay

For the cellular thermal shift assay (CETSA), HEK‐293T cells were transfected with wild‐type eEF2K or the eEF2K mutants, R144A, F8A, L10A, C146A, Y236A, and E229A plasmids, using Lipofectamine 2000 according to the manufacturer's protocol. TNBC cells and HEK‐293T cells were treated with or without **C1** overnight. Cells were then collected and equal volumes of cell suspensions were heated at different temperatures for 3 min followed by cooling to RT for an additional 3 min. Cells were then lysed by three freeze‐thaw cycles in liquid nitrogen. Lysates were centrifuged at 15 000 g for 15 min at 4 °C and the supernatant was kept for SDS‐PAGE gel and western blotting analysis.

### Co‐Immunoprecipitation

For Co‐IP, HEK‐293T cells were treated overnight, and then collected and lysed with RIPA lysis buffer (Medium) supplemented with protease inhibitors and phosphatase inhibitors on ice for 30 min, followed by centrifugation at 16 000 g for 15 min to remove debris. Cleared lysates were then subjected to immunoprecipitation with indicated primary antibody and protein A/G agarose beads at 4 °C overnight. The immunocomplexes were then washed four times with RIPA buffer the next day, and proteins were boiled in sodium dodecyl sulfate‐polyacrylamide gel electrophoresis (SDS‐PAGE) sample buffer for 10 min, followed by western blot analysis.

### Cell Viability Assay

Cell viability was measured using a CCK‐8 (Bimake, TX, USA) reagent. Briefly, 3 × 10^3^ cells per well were plated in a 96‐well‐plate and exposed to various concentrations of drug. After treatment, 10 µL of CCK‐8 reagent was added to each well and incubated for 2 h. The absorbance was read at 450 nm wavelength. The percentage of growth was calculated as Cell Viability (%) = [A (Compound)–A (Blank)]/[A (Control)–A (Blank)] × 100%.

### Colony‐Formation Assay

In all, 500 cells per well were seeded into a six‐well‐plate and exposed to the indicated treatment for 4 days. Then, the medium was changed every 2 days. After staining with 0.5% crystal violet (Beyotime Biotechnology, Shanghai, China) and washing with PBS, the plates were photographed. Colonies comprising of >100 cells were taken into account.

### 4.1 Wound‐Healing Assay

Cells were plated into twelve‐well plates and scratched straight with a pipette tip to generate a wound when the cells reached 90−100% confluence. Subsequently, cells were treated with **C1** over 48 h. The migration of cells was visualized under a microscope photographed at different time points (0 and 48 h).

### Migration and Invasion Assay

Cells were collected and seeded into 24‐well Transwell upper chambers (10^4^ cells per well) and treated them with **C1** at different concentrations (0, 0.2 µm, 0.4 µm). DMEM and RPMI‐1640 containing 30% FBS were added to the lower chamber and treated with **C1** at different concentrations (0, 0.2 µm, 0.4 µm). The plates were placed overnight in an incubator. After incubating for 48 h, the cells that were situated in the upper chamber were discarded and then stained the cells that were situated in the lower chamber utilizing crystal violet. After staining for 0.5 h, migratory and invasive cells were observed and counted utilizing a microscope.

### Flow‐Cytometric Analysis of Apoptosis

After treatment, cells were collected by trypsin and then washed with PBS. On the day of analysis, cells were collected and washed twice with cold PBS. The cells were suspended in 5 µL Annexin V‐FITC and 10 µL propidium iodide (PI) staining buffer (BD) and incubated in the dark for >15 min. Stained cells were analyzed by FACS.

### Protein Identification, Quantification, and KEGG Enriched Analysis

MaxQuant (version 2.1.4.0) software was used to analyze the TMT‐plexed MS/MS raw data with the following settings: Type: Reporter ion MS2: TMT6plex, TMT10plex, TMT16plex, or TMT18plex; enzyme: Trypsin/P; maximum missed cleavages: 2; fixed modification: carbamidomethyl (C); variable modifications: oxidation (M) and acetyl (protein N‐term); precursor mass tolerance: 20 ppm; fragment mass tolerance: 0.05 Da; match between runs and second peptide search was enabled. All other parameters were in default. The MS/MS data were searched for protein sequences which were downloaded from the UniProt database. The FDR threshold was set as 1% at both PSM (peptide spectrum match) and protein levels. Protein from contaminants or reverses will be removed. Statistical analysis was performed in R (version 4.0.0). The raw protein intensity will be normalized by the “medium” method, and hypergeometric‐based enrichment analysis with KEGG Pathway was performed to annotate protein sequences individually. Subcellular localization analysis was performed by WoLF PSORT. Transcription factor annotation was based on AnimalTFDB/PlantTFDB.

### shRNA and Plasmid Transfection Experiment

For plasmid transfection, Flag‐eEF2K, HA‐βTRCP, and eEF2K mutant DNA were incubated with Lipofectamine 8000 (Invitrogen) in serum‐free DMEM medium and added to cells. All protein expression was examined 48 h later. The sequences of shRNA are as follows: sheEF2K‐1#‐CGGGGAATGGCTGGATGATGA, sheEF2K‐2#‐ ATGAACAATGAAGCAGGTAAA.

### Mouse Xenograft Model

All animal experiments were approved by the Department of Laboratory Animals of Central South University. MDA‐MB‐231 cells (1 × 10^6^ per mouse) were suspended in 100 µL DMEM medium and then injected subcutaneously into the right flank of 4‐week‐old female nude mice. There were seven mice in each group. Tumor‐bearing mice were randomized into groups and treatments began when the tumor size reached 70–80mm^3^ (volume = length × width^2^ × π/6). The mice were randomly divided into designed groups and treated for the next 14 days. Tumor volume and mouse weight were measured every other day.

### Mouse Metastasis Model

For the lung metastasis model, MDA‐MB‐231‐Luc cells were suspended in DMEM medium and injected into nude recipients via the lateral tail vein. The mice were randomly divided into four groups and treated with: 1) vehicle (DMSO, 100 µL), 2) paclitaxel (5 mg kg^−1^, peritoneal injection), 3) **C1** (5 mg kg^−1^, peritoneal injection), or 4) **C1** (10 mg kg^−1^, peritoneal injection), and then treated every other day. Mice were killed 30 days after cell injection. The lung was excised and fixed in paraformaldehyde neutral buffer solution. Tumor nodules were counted with a 40‐fold stereomicroscope and the lung sections (4 mm) were stained with H&E.^[^ ^25^ ^]^

### Blood Samples and Analyses for In Vivo Toxicity

Blood samples were analyzed 24 h after finishing the treatments, with bleeding from the eyeball. Serum biochemical parameters for lung, spleen, kidney, liver, and blood toxicity were measured for each animal by the Department of Pathology, The Second Xiangya Hospital, Central South University. These parameters included blood urea nitrogen (UREA), aspartate aminotransferase (AST), alanine aminotransferase (ALT), creatinine (CREA), and lactic dehydrogenase (LDH).

### Western Blot Analysis

After the indicated treatment, cells were lysed in RIPA buffer containing a protease inhibitor cocktail and phosphatase inhibitor. The cell lysate was centrifuged at 12000 × rpm at 4 °C for 15 min. The protein concentration of the supernatant was determined by Bradford assay. Furthermore, 20 µg of protein samples were subjected to SDS‐PAGE and transferred to the PVDF membranes. The membranes were then blocked with 5% skim milk for 1 h at room temperature and incubated with the primary antibody at 4 °C overnight, followed by secondary antibodies for 1 h at room temperature. The protein signals were detected by an enhanced chemiluminescence kit.

### Immunohistochemistry (IHC)

Immunohistochemical analysis of the fixed, paraffin‐embedded tissue was performed using Ki67 and eEF2K antibodies according to the manufacturer's protocol.

### Coefficient of Drug Interaction (CDI)

The coefficient of drug interaction (CDI) was tested with the CCK‐8 assay of each compound at various single dose concentrations and combination concentrations. CDI = AB (PTX + **C1** treatment)/[(A, PTX treatment alone) × (B, **C1** treatment alone)]. Values less than one indicate synergism, values equal to one indicate additive effects and values greater than one indicate antagonism.^[^ ^26^ ^]^

### Reagent and Antibodies

Streptavidin magnetic beads (Cat# 22305‐1) were purchased from Beaverbio (Suzhou, China), Protein A/G agarose beads (Cat# P2108) were obtained from Beyotime Biotechnology (Shanghai, China).

Fetal Bovine Serum (Cat# FND500) was purchased from ExCell Bio (Shanghai, China), SDS‐PAGE Loading Buffer (Cat# WB2001) was purchased from New Cell & Molecular Biotech (Suzhou, China). MLN4924 (Cat# T6332) was obtained from Targetmol (Washington, USA), MG132 (Cat# S2619), and Paclitaxel (Cat# S1150) was obtained from Selleck (Huston, TX, USA).

The anti‐eEF2K antibody (Cat# ab85721, RRID:AB_2 097 314) was obtained from Abcam (Cambridge, UK). Antibodies against GAPDH (Cat# GB11002, RRID:AB_2 904 017), Ki‐67 (Cat# GB111499, RRID:AB_2 927 572) was purchased from Servicebio (Wuhan, China). Antibodies against HA (Cat# 3724, RRID:AB_1 549 585), Myc (Cat# 2278, RRID:AB_490 778), Bcl‐2 (Cat# 15 071, RRID:AB_2 744 528) were purchased from Cell Signaling Technology (Danvers, MA, USA). The anti‐Flag (Cat# M185‐3L, RRID:AB_11 123 930) antibody was obtained from MBL (Japan). Antibodies against N‐Cadherin (Cat# ET1607‐37), Vimentin (Cat# M1412‐1), E‐Cadherin (Cat# ET1607‐75) were purchased from HUABIO (Hangzhou, China). Anti‐PARP1 (Cat# 13371‐1‐AP, RRID:AB_2 160 459) and anti‐Bax (Cat# 50599‐2‐Ig, RRID:AB_2 061 561) antibodies were purchased from Proteintech (Chicago, IL, USA).

### Statistical Analysis

All the experimental data were analyzed using GraphPad Prism (version 8.0.1). The results represent three or more independent experiments, and the data were performed as the mean ± SD (standard deviation). Comparisons between groups were performed using a two‐tailed independent sample Student's *t*‐test. *p* < 0.05 was considered a significant difference (^*^ *p* < 0.05, ^**^ *p* < 0.01, ^***^ *p* < 0.001).

## Conflict of Interest

The authors declare no conflict of interest.

## Author Contributions

C.Z. and R.Z. contributed equally to this work. Y.C. and N.Y. conceived and directed the project. C.Z. contributed to experimental design and program strategy. R.Z. and X.Z. contributed to design and chemical synthesis of compounds. S.T. performed molecular docking. C.Z., T.J., X.W., and L.H. contributed to the in vitro biology experiments and data analysis. C.Z., R.G., and S.J. contributed to the in vivo biology experiments and data analysis. Z.C. and J.Y. provided technological support in the experiments. C.Z., R.Z. Y.C. and N.Y wrote the manuscript. Y.C. and N.Y. reviewed and edited the paper. All authors read and approved the final manuscript.

## Supporting information

Supporting Information

## Data Availability

All data generated or analyzed during this study are included either in this article or in Supplementary Figures.
