# Supplementary material for: Design and Characterization of a Novel eEF2K Degrader with Potent Therapeutic Efficacy Against Triple‐Negative Breast Cancer
Source: Adv Sci (Weinh). 2023 Dec 12;11(5):2305035. doi: 10.1002/advs.202305035 (PMC10837347; doi:10.1002/advs.202305035)
Supplement: Supplementary file 1 — Supporting Information [file ADVS-11-2305035-s001.pdf]

ADVANCED SCIENCE
Open Access

Supporting Information

for *Adv. Sci.*, DOI 10.1002/advs.202305035

Design and Characterization of a Novel eEF2K Degrader with Potent Therapeutic Efficacy Against Triple-Negative Breast Cancer

Changxin Zhong, Rongfeng Zhu, Ting Jiang, Sheng Tian, Xiaobao Zhao, Xiaoya Wan, Shilong Jiang, Zonglin Chen, Rong Gong, Linhao He, Jin-Ming Yang, Na Ye and Yan Cheng**

Table of Contents

Synthesis of compounds **D2**-**D4**, **I1**-**I7**, **J4** and **L1**-**L16**---------------------------2

Spectra of compounds **D2**-**D4**, **I1**-**I7**, **J4** and **L1**-**L16**----------------------------18

General Information

All commercially available starting materials and solvents were of reagent grade and used without further purification. Reactions were performed under a nitrogen atmosphere in dry glassware with magnetic stirring. Column chromatography was carried out on silica gel (200–300 mesh). All reactions were monitored by UV (254 nm) via thin layer chromatography (TLC) on silica gel plates. NMR spectra were recorded on a Bruker-600, Bruker-400 or Vnmrs-300 (^{1}H, 400 and 300 MHz; ^{13}C, 151 and 101 MHz) spectrometer. ^{1}H and ^{13}C NMR spectra were recorded with tetramethylsilane (TMS) as an internal reference. Chemical shifts are expressed in parts per million, and *J* values are given in Hz. High-resolution mass spectra (HRMS) were obtained using a Waters Xevo G2-XS Tof (ESI) mass spectrometer and GCT Premier TM (EI) mass spectrometer. The purities of the final compounds were established by analytical HPLC. HPLC analysis conditions: COSMOSIL 5C18-MS-II, 3.5 μm, 4.6 mm × 250 mm; flow rate 1 mL/min; UV detection at 254 nm; linear gradient from 10% ACN in water to 100% ACN in water in 30 min. All biologically evaluated compounds were >95% pure.

Preparation of 3-benzyl-1-cyclopropyl-5-(pyridin-2-yl)pyrimidine-2,4(1*H*,3*H*)-dione (D2) and 1,3-dibenzyl-5-(pyridin-2-yl)pyrimidine-2,4(1*H*,3*H*)-dione (D3)

To a mixture of pyrimidine-2,4(1*H*, 3*H*)-dione **A1** (1000 mg, 8.92 mmol), K_2CO_3 (2000 mg, 17.84 mmol) and acetonitrile (100 mL), benzyl bromide (8.92 mmol) was added dropwise and the reaction mixture was stirred for 12 h. Upon completion of the reaction as indicated by TLC, the reaction mixture was decompressed, evaporated and extracted with water (20 mL) and dichloromethane (DCM, 30 mL). Then the organic phase was washed with brine (20 mL × 2) and dried over anhydrous sodium sulfate. The organic phase was purified with column chromatography (DCM : MeOH = 80 : 1) to get compound **B1** as a white solid (367 mg, yield 20 %) and **B3** as a colorless oil (1000 mg, yield 38%). **B1**: ^{1}H NMR (300 MHz, $CDCl_3$) δ 9.17 (s, 1H), 7.42 – 7.28 (m, 5H), 7.16 (d, *J* = 8.0 Hz, 1H), 5.70 (d, *J* = 7.9 Hz, 1H), 4.92 (s, 2H). **B3**: ^{1}H NMR (300 MHz, $CDCl_3$) δ 7.51 – 7.44 (m, 2H), 7.41 – 7.21 (m, 8H), 7.10 (d, *J* = 7.9 Hz, 1H), 5.74 (d, *J* = 7.9 Hz, 1H), 5.14 (s, 2H), 4.90 (s, 2H).

To a mixture of pyrimidine-2,4(1*H*, 3*H*)-dione **B1** (200 mg, 0.99 mmol), Na_2CO_3 (210 mg，1.98 mmol), cyclopropylboronic acid (102 mg, 1.19 mmol) and DCE (2 mL), the mixture of 2,2'-dipyridyl and $Cu(OAc)_2$ in DCE (2 mL) was added dropwise and the reaction mixture was stirred for 12 h. Upon completion of the reaction as indicated by TLC, the reaction mixture was decompressed, evaporated and extracted with 1N HCl (20 mL) and DCM (30 mL). Then the organic phase was washed with brine (20 mL × 2) and dried over anhydrous sodium sulfate. The organic phase was purified with column chromatography (PE : EA = 1 : 1) to get compound **B2** as a white solid (150 mg, yield 63%). ^{1}H NMR (300 MHz, $CDCl_3$) δ 7.44 – 7.27 (m, 5H), 7.14 (dd, *J* = 7.9, 1.7 Hz, 1H), 5.70 (dd, *J* = 7.9, 1.6 Hz, 1H), 4.92 (s, 2H), 2.71 (ddt, *J* = 10.2, 7.4, 4.1 Hz, 1H), 1.17 (t, *J* = 7.1 Hz, 2H), 0.88 – 0.78 (m, 2H).

To a mixture of **B2**-**B3** (1 eq), NIS (1 eq) under the atmosphere of N_2, AcOH (4 mL) was added, and the reaction mixture was stirred for 10 h at room temperature. Upon completion of the reaction as indicated by TLC, the reaction mixture was

decompressed, evaporated and extracted with water (20 mL) and DCM (30 mL). Then the organic phase was washed with brine (20 mL × 2) and dried over anhydrous sodium sulfate. The organic phase was concentrated to obtain compounds **C2**-**C3** used directly for next step. To a mixture of **C2**-**C3** (1 eq), (2-pyridinyl)tributylstannane (1 eq), CuI (0.2 eq) and $Pd(PPh_3)_2Cl_2$ (0.2 eq) under the atmosphere of N_2, THF (5 mL) was added, and the reaction mixture was stirred overnight at room temperature. Upon completion of the reaction as indicated by TLC, the reaction mixture was decompressed, evaporated and extracted with water (20 mL) and DCM (30 mL). Then the organic phase was washed with brine (20 mL × 3) and dried over anhydrous sodium sulfate. The organic phase was purified with column chromatography to obtain **D2**-**D3**.

*3-Benzyl-1-cyclopropyl-5-(pyridin-2-yl)pyrimidine-2,4(1H,3H)-dione (**D2**)*: Compound **D2** was prepared in 58% yield from **C2** (200 mg, 0.479 mmol). The title compound was obtained as a white solid. ^{1}H NMR (300 MHz, $CDCl_3$) δ 8.51 (s, 1H), 8.41 (s, 1H), 8.33 (d, *J* = 8.2 Hz, 1H), 7.69 (d, *J* = 8.1 Hz, 1H), 7.42 – 7.27 (m, 5H), 7.17 (d, *J* = 6.8 Hz, 1H), 5.04 (s, 2H), 2.77 (dq, *J* = 7.4, 3.8 Hz, 1H), 1.21 (q, *J* = 7.5, 6.9 Hz, 2H), 0.85 (dd, *J* = 6.8, 4.2 Hz, 2H). ^{13}C NMR (151 MHz, $CDCl_3$) δ 150.7, 135.5, 129.0, 128.4, 128.1, 122.3, 53.0, 25.4, 8.8. HRMS (ESI) calcd for $C_{19}H_{18}N_3O_2$ $[M+H]^+$: 320.1399, found 320.1400.

*1,3-Dibenzyl-5-(pyridin-2-yl) pyrimidine-2,4(1H,3H)-dione (**D3**)*: Compound **D3** was prepared in 58% yield from **C3** (205 mg, 0.491 mmol). The title compound was obtained as a white solid. ^{1}H NMR (300 MHz, $CDCl_3$) δ 8.51 (s, 1H), 8.42 (s, 1H), 8.33 (d, *J* = 7.9 Hz, 1H), 7.68 (d, *J* = 7.9 Hz, 1H), 7.51 (d, *J* = 6.8 Hz, 2H), 7.37 – 7.25 (m, 8H), 7.18 (d, *J* = 6.2 Hz, 1H), 5.25 (s, 2H), 5.07 (s, 2H). ^{13}C NMR (151 MHz, $CDCl_3$) δ 161.6, 151.2, 150.7, 148.7, 143.1, 136.7, 135.4, 129.1, 129.0, 128.4, 128.4, 128.0, 127.6, 123.1, 122.4, 53.1, 44.9. HRMS (EI) calcd for $C_{23}H_{19}N_3O_2$ $[M]^+$: 369.1477, found 369.1479.

Preparation of 1,3-dibenzyl-5-phenylpyrimidine-2,4(1*H*,3*H*)-dione (D4)

To a mixture of **C3** (100 mg, 0.24 mmol), phenylboronic acid (44 mg, 0.36

mmol), Na_2CO_3 (76 mg, 0.72 mmol) and $Pd(PPh_3)_4$ (28 mg, 0.024 mmol) under the atmosphere of N_2, water and acetonitrile (10 mL, 2:1) was added and the reaction mixture was refluxed overnight. Upon completion of the reaction as indicated by TLC, the reaction mixture was decompressed, evaporated and extracted with water (20 mL) and DCM (30 mL). Then the organic phase was washed with brine (20 mL × 2) and dried over anhydrous sodium sulfate. The organic phase was purified with column chromatography (PE:EA = 7:1) to obtain **D4** as a white solid in 45% yield. ^1H NMR (300 MHz, $CDCl_3$) δ 7.49 (d, J = 7.1 Hz, 2H), 7.37 (d, J = 7.9 Hz, 2H), 7.30 (t, J = 4.2 Hz, 4H), 7.28 – 7.16 (m, 8H), 5.17 (s, 2H), 4.93 (s, 2H). ^{13}C NMR (101 MHz, $CDCl_3$) δ 151.4, 139.4, 136.9, 135.3, 132.8, 129.4, 129.2, 128.6, 128.5, 128.4, 128.4, 128.1, 128.0, 127.7, 115.1, 52.5, 45.0. HRMS (EI) calcd for $C_{24}H_{20}N_2O_2$ $[M]^+$: 368.1525, found 368.1522.

Preparation of Compounds I1-I6

General procedure for the preparation of G1-G4: To a mixture of **E1-E4** (41.26 mmol), concentrated hydrochloric acid (22.5 mL) and water (60 mL), an aqueous solution of $NaNO_2$ (60.0 mL, 57.76 mmol) was added dropwise below 0 °C, and the reaction mixture was stirred for 0.5 h to generate a diazonium salt solution. Subsequently, ethyl (2-cyanoacetyl)carbamate **F1** (6.7 g, 41.26 mmol) and sodium acetate (10 g, 123.78 mmol) were dissolved in a solution of pyridine and water (50 mL, 1:1), and this mixture was then added dropwise to the previously resulting diazonium salt solution below 0 ℃ and stirred for an additional 2 h. The precipitate was collected by filtration, washed with water (40 mL × 3), and dried to give compound **G1-G4**.

Ethyl-(E)-(2-cyano-2-(2-phenylhydrazineylidene)acetyl)carbamate (G1): Compound **G1** was prepared as a yellow solid in 89% yield from **E1** (500 mg, 5.37 mmol) and **F1** (1010 mg, 6.44 mmol). ^1H NMR (300 MHz, DMSO-d_6) δ 12.08 (s, 1H), 10.58 (s, 1H), 7.72 (d, J = 6.8 Hz, 2H), 7.39 (t, J = 6.6 Hz, 2H), δ 7.17 (t, J = 7.9 Hz, 1H), 4.16 (q, J = 8.3 Hz, 2H) 1.29 – 1.25 (m, 3H).

Ethyl-(E)-(2-cyano-2-(2-(m-tolyl)hydrazineylidene)acetyl)carbamate ***(G2)***: Compound **G2** was prepared as a yellow solid in 92% yield from **E2** (513 mg, 4.79 mmol) and **F1** (897 mg, 5.74 mmol). ^{1}H NMR (300 MHz, $CDCl_3$) δ 9.68 (s, 1H), 8.55 (s, 1H), 7.31 (dd, *J* = 9.8, 6.6 Hz, 1H), 7.14 (s, 2H), 7.05 (d, *J* = 7.6 Hz, 1H), 4.33 (q, *J* = 7.0 Hz, 2H), 2.41 (s, 3H), 1.37 (t, *J* = 7.0 Hz, 3H).

Ethyl-(E)-(2-cyano-2-(2-(p-tolyl)hydrazineylidene)acetyl)carbamate ***(G3)***: Compound **G3** was prepared as a yellow solid in 91% yield from **E3** (521 mg, 4.86 mmol) and **F1** (911 mg, 5.83 mmol). ^{1}H NMR: (300 MHz, $CDCl_3$) δ 9.82 (s, 1H), 8.56 (s, 1H), 7.30-7.20 (m, 4H), 4.37 – 4.28 (q, *J* = 7.1 Hz, 2H), 2.37 (s, 3H), 1.36 (t, *J* = 7.1 Hz, 3H).

Ethyl-(E)-(2-cyano-2-(2-(3,4-dimethylphenyl)hydrazineylidene)acetyl)carbamate ***(G4)***: Compound **G4** was prepared as a yellow solid in 88% yield from **E4** (1000 mg, 8.25 mmol) and **F1** (1551 mg, 9.90 mmol). ^{1}H NMR (300 MHz, DMSO-d_6) δ 11.95 (s, 1H), 10.42 (d, *J* = 4.5 Hz, 1H), 7.50 – 7.35 (m, 2H), 7.11 (d, *J* = 7.9 Hz, 1H), 4.15 (tt, *J* = 7.7, 4.7 Hz, 2H), 2.18 (d, *J* = 11.3 Hz, 6H), 1.41 – 1.16 (m, 3H).

General procedure for the preparation of H1-H4: A mixture of compounds **G1-G4** (1 eq, 34.82 mmol), sodium acetate (5 eq, 174.12 mmol) and glacial acetic acid (120 mL) was stirred at reflux for 2 h. Subsequently, upon cooling to room temperature, the mixture was poured into water (500 mL) and then stirred for 0.5 h. The precipitate was collected by filtration, and then washed successively with water (60 mL ×2) and petroleum ether (40 mL × 2) and dried to give compounds **H1**-**H4** as yellowish solids.

3,5-Dioxo-2-phenyl-2,3,4,5-tetrahydro-1,2,4-triazine-6-carbonitrile ***(H1)***: Compound **H1** was prepared as a yellowish solid in 91% yield from **G1** (500 mg, 1.92 mmol). ^{1}H NMR (300 MHz, DMSO-d_6) δ 12.64 (s, 1H), 8.01 – 7.95 (m, 2H), 7.52 – 7.51 (m, 2H), 7.46 – 7.40 (m, 1H).

3,5-Dioxo-2-(m-tolyl)-2,3,4,5-tetrahydro-1,2,4-triazine-6-carbonitrile ***(H2)***: Compound **H2** was prepared as a yellowish solid in 90% yield from **G2** (500 mg, 1.82 mmol). ^{1}H NMR (300 MHz, $CDCl_3$) δ 12.80 (s, 1H), 8.01 – 7.96 (m, 1H), 7.38 (t, *J* = 7.0 Hz, 1H), 7.30 (t, *J* = 9.3 Hz, 2H), 2.37 (s, 3H).

3,5-Dioxo-2-(p-tolyl)-2,3,4,5-tetrahydro-1,2,4-triazine-6-carbonitrile ***(H3)***: Compound **H3** was prepared as a yellowish solid in 90% yield from **G3** (500 mg, 1.82 mmol). ^{1}H NMR (300 MHz, DMSO-d_6) δ 12.99 (s, 1H), 7.34 (q, *J* = 8.4 Hz, 4H), 2.36 (s, 3H).

2-(3,4-Dimethylphenyl)-3,5-dioxo-2,3,4,5-tetrahydro-1,2,4-triazine-6-carbonitrile (**H4**): Compound **H4** was prepared as a yellowish solid in 92% yield from **G4** (500 mg, 1.73 mmol). ^{1}H NMR (300 MHz, $CDCl_3$) δ 9.31 (s, 1H), 7.36-7.24 (m, 3H), 2.40 (s, 6H).

General procedure for the preparation of compounds I1-I6 and I8-I10: To a solution of **H1-H4** (1 eq) and potassium carbonate (2 eq) in acetonitrile, various alkyl halides (1.1 eq) were added dropwise and stirred for 4 h. The reaction mixture was decompressed, evaporated and extracted with water (20 mL) and DCM (30 mL). The organic phase was concentrated, recrystallized, filtered, and then dried to give compounds **I1-I6** and **I8-I10**.

4-Ethyl-3,5-dioxo-2-phenyl-2,3,4,5-tetrahydro-1,2,4-triazine-6-carbonitrile ***(I1)***: Compound **I1** was prepared as a white solid in 35% yield from **H1** (500 mg, 2.33 mmol) and iodoethane (437 mg, 2.80 mmol). ^{1}H NMR (300 MHz, $CDCl_3$) δ 7.55-7.45 (m, 5H), 4.19 (q, *J* = 7.2 Hz, 2H), 1.42 (t, *J* = 7.1 Hz, 3H). ^{13}C NMR (151 MHz, $CDCl_3$) δ 152.7, 146.8, 139.2, 129.5, 129.2, 125.0, 121.8, 111.1, 37.6, 12.2. HRMS (EI) calcd for $C_{12}H_{10}N_4O_2$ $[M]^+$: 242.0804, found 242.0806.

4-Benzyl-3,5-dioxo-2-(m-tolyl)-2,3,4,5-tetrahydro-1,2,4-triazine-6-carbonitrile ***(I2)***: Compound **I2** was prepared as a white solid in 72% yield from **H2** (1400 mg, 6.13 mmol) and (bromomethyl)benzene (1150 mg，6.75 mmol). ^{1}H NMR (300 MHz, $CDCl_3$) δ 7.55 – 7.46 (m, 2H), 7.40 – 7.28 (m, 4H), 7.28 – 7.17 (m, 3H), 5.17 – 5.08 (m, 2H), 2.39 (t, *J* = 3.8 Hz, 3H). ^{13}C NMR (151 MHz, $CDCl_3$) δ 152.88, 147.1, 139.5, 139.1, 134.1, 130.4, 130.1, 129.0, 128.8, 128.8, 125.6, 122.2, 121.7, 111.1, 45.4, 21.3. HRMS (EI) calcd for $C_{18}H_{14}N_4O_2$ $[M]^+$: 318.1117, found 318.1115.

4-Benzyl-3,5-dioxo-2-(p-tolyl)-2,3,4,5-tetrahydro-1,2,4-triazine-6-carbonitrile ***(I3)***: Compound **I3** was prepared as a white solid in 82% yield from **H3** (1400 mg，

6.13 mmol) and (bromomethyl)benzene (1150 mg，6.75 mmol). ^{1}H NMR (300 MHz, $CDCl_3$) δ 7.53-7.32 (m, 9H), 5.15 (s, 2H), 2.41 (s, 3H). ^{13}C NMR (151 MHz, $CDCl_3$) δ 152.9, 147.1, 139.9, 136.7, 134.1, 130.1, 129.8, 128.8, 128.8, 124.8, 121.7, 111.1, 45.4, 21.2. HRMS (EI) calcd for $C_{18}H_{14}N_4O_2$ $[M]^+$: 318.1117, found 318.1119.

*4-Benzyl-2-(3,4-dimethylphenyl)-3,5-dioxo-2,3,4,5-tetrahydro-1,2,4-triazine-6-carbonitrile (**I4**)*: Compound **I4** was prepared as a white solid in 88% yield from **H4** (1.68 g, 6.94 mmol) and (bromomethyl)benzene (1.3 g, 7.63 mmol). ^{1}H NMR (300 MHz, $CDCl_3$) δ 7.58 – 7.49 (m, 2H), 7.35 (q, J = 2.8 Hz, 3H), 7.26 – 7.15 (m, 3H), 5.16 (s, 2H), 2.31 (s, 6H).^{13}C NMR (151 MHz, $CDCl_3$) δ 152.9, 147.1, 138.6, 137.9, 136.8, 134.2, 130.2, 130.1, 128.8, 128.8, 125.9, 122.4, 121.5, 111.1, 45.4, 19.9, 19.5. HRMS (EI) calcd for $C_{19}H_{16}N_4O_2$ $[M]^+$: 332.1273, found 332.1270.

*2-(3,4-Dimethylphenyl)-4-methyl-3,5-dioxo-2,3,4,5-tetrahydro-1,2,4-triazine-6-carbonitrile (**I5**)*: Compound **I5** was prepared as a white solid in 73% yield from **H4** (1000 mg，4.13 mmol) and iodomethane (703 mg，4.95 mmol). ^{1}H NMR (300 MHz, $CDCl_3$) δ 7.25 – 7.15 (m, 3H), 3.46 (s, 3H), 2.32 (s, 6H). ^{13}C NMR (101 MHz, $CDCl_3$) δ 153.1, 147.3, 138.6, 138.0, 136.9, 130.3, 125.9, 122.4, 121.1, 111.1, 28.2, 19.9, 19.5. HRMS (EI) calcd for $C_{13}H_{12}N_4O_2$ $[M]^+$: 256.0960, found 256.0963.

*3,5-Dioxo-2-phenyl-4-propyl-2,3,4,5-tetrahydro-1,2,4-triazine-6-carbonitrile (**I6**)*: Compound **I6** was prepared as a white solid in 19% yield from **H1** (700 mg，2.89 mmol) and 1-iodopropane (589 mg，3.47 mmol). ^{1}H NMR (300 MHz, $CDCl_3$) δ 7.26 – 7.17 (m, 3H), 3.98 (t, J = 7.6 Hz, 2H), 2.32 (s, 6H), 1.74 (q, J = 7.6 Hz, 2H), 0.99 (t, J = 8.4 Hz, 3H). ^{13}C NMR (101 MHz, $CDCl_3$) δ 153.0, 147.1, 138.6, 138.0, 136.9, 130.2, 125.9, 122.4, 121.4, 111.2, 43.8, 20.4, 19.9, 19.6, 11.2. HRMS (EI) calcd for $C_{15}H_{16}N_4O_2$ $[M]^+$: 284.1273, found 284.1271.

*4-Benzyl-3,5-dioxo-2-phenyl-2,3,4,5-tetrahydro-1,2,4-triazine-6-carbonitrile (**I8**)*: Compound **I8** was prepared as a yellowish solid in 78% yield from **H1** (250 mg, 1.17 mmol) and iodomethane (219.6 mg, 1.28 mmol) and used directly for next step without purification.

*4-(3-Fluorobenzyl)-2-(3,4-dimethylphenyl)-3,5-dioxo-2,3,4,5-tetrahydro-1,2,4-triazine-6-carbonitrile (**I9**)*: Compound **I9** was prepared as a white solid in 74% yield

from **H4** (2000 g, 8.26 mmol) and 1-(bromomethyl)-3-fluorobenzene (1720 mg, 9.08 mmol). ^{1}H NMR (300 MHz, $CDCl_3$) δ 7.37 – 7.12 (m, 6H), 7.09 – 6.94 (m, 1H), 5.14 (s, 2H), 2.31 (s, 6H). ^{13}C NMR (101 MHz, $CDCl_3$) δ 162.9 (d, *J* = 247.7 Hz), 153.0, 147.2, 138.9, 138.2, 136.9, 136.3 (d, *J* = 7.7 Hz), 130.5 (d, *J* = 8.6 Hz), 130.4, 126.1, 125.8, 122.5, 121.6, 117.1 (d, *J* = 22.1 Hz), 116.0 (d, *J* = 20.9 Hz), 111.2, 45.0, 20.0, 19.7. HRMS (EI) calcd for $C_{19}H_{15}FN_4O_2$ $[M]^+$: 350.1179, found 350.1178.

*4-(3-Fluorobenzyl)-2-(3,4-dimethylphenyl)-3,5-dioxo-2,3,4,5-tetrahydro-1,2,4-triazine-6-carbonitrile **(I10)***: Compound **I10** was prepared as a white solid in 74% yield from **H4** (500 mg, 2.06 mmol) and 1-(bromomethyl)-4-fluorobenzene (468 mg, 2.48 mmol). ^{1}H NMR (300 MHz, $CDCl_3$) δ 7.55 (dd, *J* = 5.9, 2.7 Hz, 2H), 7.39 – 7.10 (m, 3H), 7.04 (t, *J* = 8.7 Hz, 2H), 5.15 (s, 2H), 2.34 (s, 6H). ^{13}C NMR (151 MHz, $CDCl_3$) δ 163.0 (d, *J* = 248.2 Hz), 153.0, 147.2, 138.8, 138.1, 136.9, 132.3, 132.3, 130.4, 130.1 (d, *J* = 3.3 Hz), 126.1, 122.5, 121.6, 115.9, 115.8, 111.2, 44.7, 20.0, 19.7. HRMS (ESI) calcd for $C_{19}H_{15}FN_4O_2Na$ $[M+Na]^+$: 373.1077, found 373.1069.

Preparation of 4-benzyl-2-(3,4-dimethylphenyl)-3,5-dioxo-2,3,4,5-tetrahydro-1,2,4-triazine-6-carboxamide (I7)

I4 (100 mg, 300.88 mmol), NaOH (6 mg) and DMSO (0.015 mL) were dissolved in EtOH (2 mL) below 0 °C. Then, 30% H_2O_2 was added dropwise, and the reaction mixture was stirred overnight. The reaction mixture was extracted with DCM, and the organic phase was washed with saturated sodium chloride solution. The organic phase was further purified with silica gel chromatography (DCM: MeOH = 80: 1) and concentrated to obtain a white solid (50 mg, 47.43%). ^{1}H NMR (300 MHz, $CDCl_3$) δ 8.43 (s, 1H), 7.50 – 7.44 (m, 2H), 7.30 – 7.25 (m, 3H), 7.19 (d, *J* = 2.1 Hz, 3H), 5.92 (s, 1H), 5.15 (s, 2H), 2.22 (d, *J* = 3.2 Hz, 6H). ^{13}C NMR (151 MHz, $CDCl_3$) δ 160.1, 155.8, 147.9, 138.1, 137.8, 137.3, 134.6, 132.8, 130.1, 129.8, 128.7, 128.5, 126.3, 122.8, 45.1, 19.9, 19.5. HRMS (EI) calcd for $C_{19}H_{18}N_4O_3$ $[M]^+$: 350.1379, found 350.1378.

General procedure for the preparation of J2-J4 and J8-J10:

Compounds **I2-I4** and **I8-I10** (4.52 mmol) was dissolved in a mixture of concentrated hydro chloric acid (16 mL) and glacial acetic acid (32 mL) and refluxed for 4 h. The solvent was evaporated in vacuo, and then water (50 mL) was added. Subsequently, the precipitate was filtrated and stirred in anhydrous ether (30 mL) for 10 min. Compounds **J2-J4** and **J8-J10** were obtained by filtration and dryness under vacuum without purification.

4-Benzyl-2-(3,4-dimethylphenyl)-3,5-dioxo-2,3,4,5-tetrahydro-1,2,4-triazine-6-carboxylic acid (J4): Compound **J4** was prepared as a white solid in 74% yield from **I4**. ^{1}H NMR (300 MHz, DMSO-d_6) δ 7.46 (s, 1H), δ 7.38 (d, J = 7.8 Hz, 2H), 7.34 (s, 3H), 7.29 (d, J = 9.3 Hz, 2H), 7.25 (s, 1H), 5.01 (s, 2H), 2.26 (s, 6H). ^{13}C NMR (151 MHz, DMSO-d_6) δ 161.8, 153.3, 148.2, 137.8, 136.9, 136.8, 135.6, 134.9, 129.6, 128.4, 128.0, 127.5, 126.7, 123.3, 43.9, 19.3, 19.0. HRMS (ESI) calcd for $C_{19}H_{17}N_3O_4Na$ [M+Na]$^+$: 374.1117, found 374.1116.

General procedure for the synthesis of compounds L1-L16

To a solution of **J2-J4** and **J8-J10** (100 mg, 300.88 mmol) in DCM (5 mL), one drop of DMF and oxalyl dichloride (0.12 mL) was added dropwise under the atmosphere of N_2 and then stirred at room temperature for 3 h. The reaction mixture was concentrated to dryness to obtain the corresponding acyl chlorides.

To the solution of the corresponding amines (300.88 mmol) in DCM 5 mL with Et_3N (60.89 mg, 601.76 mmol), aforementioned acyl chlorides were added dropwise by keeping the temperature below 0 °C. Then, the reaction mixture was stirred at rt for 3 h. Upon completion of the reaction as indicated by TLC, the mixture was washed successively with water (20 mL × 3) and brine (20 mL × 2), and the organic phase was dried over anhydrous sodium sulfate. The solid was removed by filtration, and the filtrate was purified with silica gel chromatography to yield title compounds **L1-L16**.

*Ethyl 4-benzyl-2-(3,4-dimethylphenyl)-3,5-dioxo-2,3,4,5-tetrahydro-1,2,4-triaz-6-carboxylate (**L1**)*: Compound **L1** was prepared as a white solid in 96% yield from **J4** (52 mg, 0.15 mmol) and ethanol (0.1 mL). ^{1}H NMR (300 MHz, DMSO-d_6) δ 7.39

(d, J = 7.7 Hz, 2H), 7.33 (s, 1H), 7.30 (d, J = 2.2 Hz, 2H), 7.29 – 7.21 (m, 3H), 5.00 (s, 2H), 4.30 (q, J = 7.1 Hz, 2H), 2.26 (s, 6H), 1.26 (t, J = 7.0 Hz, 3H). ^{13}C NMR (151 MHz, DMSO-d_6) δ 160.4, 153.1, 148.2, 137.8, 137.1, 136.9, 135.5, 133.8, 129.6, 128.3, 128.0, 127.4, 126.8, 123.4, 61.8, 44.0, 19.3, 19.0, 13.9. HRMS (ESI) calcd for $C_{21}H_{21}N_3O_4Na$ [M+Na]$^+$: 402.1430, found 402.1434

*Methyl 3-(4-benzyl-2-(3,4-dimethylphenyl)-3,5-dioxo-2,3,4,5-tetrahydro-1,2,4-triazine-6-carboxamido)propanoate (**L2**)*: Compound **L2** was prepared as a white solid in 90% yield from **J4** (400 mg, 1.10 mmol) and methyl 3-aminopropanoate (126 mg, 0.91 mmol). ^{1}H NMR (300 MHz, DMSO-d_6) δ 8.80 (s, 1H), 7.43 – 7.19 (m, 8H), 5.03 (s, 2H), 3.61 (d, J = 5.1 Hz, 3H), 3.48 (q, J = 6.5 Hz, 2H), 2.57 (t, J = 6.7 Hz, 2H), 2.27 (s, 6H). ^{13}C NMR (101 MHz, $CDCl_3$) δ 172.5, 159.1, 155.8, 147.9, 137.9, 137.7, 137.5, 134.7, 133.1, 130.0, 129.8, 128.7, 128.5, 126.3, 122.7, 51.9, 45.0, 35.3, 33.7, 19.8, 19.5. HRMS (ESI) calcd for $C_{23}H_{25}N_4O_5$ [M+H]$^+$: 437.1825, found 437.1825.

*4-Benzyl-N-(2-(dimethylamino)ethyl)-2-(3,4-dimethylphenyl)-3,5-dioxo-2,3,4,5-tetrahydro-1,2,4-triazine-6-carboxamide (**L3**)*: Compound **L3** was prepared as a white solid in 50% yield from **J4** (210 mg, 0.57 mmol) and N^1,N^1-dimethylethane-1,2-diamine (41.82 mg, 0.48 mmol). ^{1}H NMR (300 MHz, $CDCl_3$) δ 8.80 (s, 1H), 7.56 – 7.50 (m, 2H), 7.38 – 7.30 (m, 3H), 7.26 – 7.16 (m, 3H), 5.20 (s, 2H), 3.60 – 3.54 (m, 2H), 2.55 (t, J = 6.2 Hz, 2H), 2.31 (s, 6H), 2.28 (s, 6H). ^{13}C NMR (101 MHz, $CDCl_3$) δ 159.1, 155.7, 147.9, 137.8, 137.6, 137.5, 134.8, 133.5, 130.0, 129.7, 128.7, 128.5, 126.3, 122.7, 57.9, 45.3, 44.9, 37.6, 19.8, 19.5. HRMS (ESI) calcd for $C_{23}H_{28}N_5O_3$ [M+H]$^+$: 422.2192, found 422.2187.

*Methyl 4-(4-benzyl-2-(3,4-dimethylphenyl)-3,5-dioxo-2,3,4,5-tetrahydro-1,2,4-triazine-6-carboxamido)benzoate (**L4**)*: Compound **L4** was prepared as a white solid in 50% yield from **J4** (210 mg，0.57 mmol) and methyl 4-aminobenzoate (71.7 mg，0.48 mmol). ^{1}H NMR (300 MHz, DMSO-d_6) δ 10.95 (s, 1H), 7.97 (d, J = 7.7 Hz, 2H), 7.80 (d, J = 8.4 Hz, 2H), 7.48 – 7.20 (m, 8H), 5.07 (s, 2H), 3.83 (s, 3H), 2.26 (s, 6H). ^{13}C NMR (151 MHz, DMSO-d_6) δ 166.1, 159.1, 154.9, 148.5, 142.6, 138.2, 137.5, 137.4, 137.2, 135.9, 130.9, 130.1, 128.9, 128.7, 128.1, 127.0, 125.6, 123.6, 119.8,

52.5, 44.6, 40.5, 19.8, 19.5. HRMS (ESI) calcd for $C_{27}H_{25}N_4O_5$ $[M+H]^+$: 485.1825, found 485.1826.

*4-Benzyl-2-(3,4-dimethylphenyl)-N,N-dimethyl-3,5-dioxo-2,3,4,5-tetrahydro-1,2,4-triazine-6-carboxamide (**L5**)*: Compound **L5** was prepared in 41% yield from **J4** (110 mg, 0.30 mmol) and dimethylamine (12.19 mg, 0.27 mmol). The title compound was obtained as a white solid. HPLC purity 100.0% (t_R = 11.72 min). ^{1}H NMR (300 MHz, $CDCl_3$) δ 7.56 (s, 2H), 7.37 – 7.30 (m, 3H), 7.23 (d, *J* = 11.3 Hz, 3H), 5.19 (s, 2H), 3.12 (s, 3H), 3.03 (s, 3H), 2.30 (s, 6H). ^{13}C NMR (101 MHz, $CDCl_3$) δ 161.7, 153.7, 148.1, 139.6, 137.6, 137.5, 137.4, 135.0, 130.0, 128.7, 128.4, 126.5, 122.6, 44.7, 38.1, 35.0, 19.9, 19.5. HRMS (EI) calcd for $C_{21}H_{22}N_4O_3$ $[M]^+$: 378.1692, found 378.1689. HPLC purity 99.85% (t_R = 22.11 min).

*Ethyl 1-(4-benzyl-2-(3,4-dimethylphenyl)-3,5-dioxo-2,3,4,5-tetrahydro-1,2,4-triazine-6-carbonyl) piperidine-3-carboxylate (**L6**)*: Compound **L6** was prepared in 62% yield from **J4** (105 mg, 0.284 mmol) and ethyl piperidine-3-carboxylate (40.58 mg, 0.258 mmol). The title compound was obtained as a white solid. HPLC purity 100.0% (t_R = 12.57 min). ^{1}H NMR (300 MHz, $CDCl_3$) δ 7.57 – 7.51 (m, 2H), 7.33 – 7.30 (m, 3H), 7.23 (d, *J* = 5.8 Hz, 1H), 7.19 (s, 2H), 5.16 (s, 2H), 4.72 (d, *J* = 13.6 Hz, 0.5H), 4.42 (d, *J* = 13.3 Hz, 0.5H), 4.19 – 4.08 (m, 2H), 3.75 (d, *J* = 13.8 Hz, 0.5H), 3.55 (d, *J* = 13.7 Hz, 0.5H), 3.33 (dd, *J* = 13.7, 10.2 Hz, 0.5H), 3.02 (dq, *J* = 23.1, 13.3, 11.8 Hz, 1.5H), 2.62 (s, 1H), 2.28 (d, *J* = 3.0 Hz, 6H), 2.15 (s, 1H), 1.76 (d, *J* = 15.3 Hz, 3H), 1.28 – 1.21 (m, 3H). ^{13}C NMR (101 MHz, $CDCl_3$) δ 170.3, 170.2, 161.5, 153.6, 153.6, 148.1, 148.0, 139.5, 137.5, 137.4, 137.4, 135.1, 135.0, 130.1, 130.0, 129.9, 129.9, 128.6, 128.4, 128.4, 126.2, 126.1, 122.6, 122.5, 61.6, 61.5, 57.5, 52.3, 45.1, 44.7, 44.7, 40.0, 27.5, 26.6, 25.0, 24.3, 20.9, 20.6, 19.9, 19.8, 19.5, 14.2, 14.0. HRMS (EI) calcd for $C_{27}H_{30}N_4O_5$ $[M]^+$: 490.2216, found 490.2214. HPLC purity 99.14% (t_R =24.59 min).

*Ethyl 1-(4-benzyl-2-(3,4-dimethylphenyl)-3,5-dioxo-2,3,4,5-tetrahydro-1,2,4-triazine-6-carbonyl) piperidine-2-carboxylate (**L7**)*: Compound **L7** was prepared in 63% yield from **J4** (105 mg, 0.284 mmol) and ethyl piperidine-2-carboxylate (45 mg, 0.28 mmol). The title compound was obtained as a white solid. ^{1}H NMR (300 MHz,

$CDCl_3$) δ 7.57 - 7.51 (m, 2H), 7.37 – 7.27 (m, 3H), 7.25 – 7.12 (m, 3H), 5.42 (d, *J* = 5.7 Hz, 0.7H), 5.16 (d, *J* = 6.8 Hz, 2H), 4.64 (d, *J* = 13.6 Hz, 0.4H), 4.41 (s, 0.3H), 4.23 (q, *J* = 7.1 Hz, 1H), 4.12 – 4.00 (m, 0.6H), 3.61 – 3.36 (m, 1.7H), 3.07 (t, *J* = 12.8 Hz, 0.3H), 2.35 (s, 1H), 2.28 (d, *J* = 2.4 Hz, 6H), 1.77 (d, *J* = 11.6 Hz, 2H), 1.65 – 1.35 (m, 3H), 1.29 (t, *J* = 7.2 Hz, 2H), 1.13 (t, *J* = 7.2 Hz, 1H). ^{13}C NMR (101 MHz, $CDCl_3$) δ 170.3, 170.2, 161.5, 153.6, 153.6, 148.1, 148.0, 139.5, 137.5, 137.4, 137.4, 135.1, 135.0, 130.1, 130.0, 129.9, 129.9, 128.6, 128.4, 128.4, 126.2, 126.1, 122.6, 122. 5, 61.6, 61.5, 57.5, 52.3, 45.1, 44.7, 44.7, 40.0, 27.5, 26.6, 25.0, 24.3, 20.9, 20.6, 19.9, 19.8, 19.5, 14.2, 14.0. HRMS (ESI) calcd for $C_{27}H_{31}N_4O_5$ $[M+H]^+$: 491.2294, found 491.2295. HPLC purity 99.78% (t_R =24.90 min).

*Ethyl-1-(4-benzyl-2-(3,4-dimethylphenyl)-3,5-dioxo-2,3,4,5-tetrahydro-1,2,4-triazine-6-carbonyl) piperidine-4-carboxylate (**L8**)*: Compound **L8** was prepared in 52% yield from **J4** (105 mg, 0.28 mmol) and ethyl piperidine-4-carboxylate (45 mg, 0.28 mmol). The title compound was obtained as a white solid. ^{1}H NMR (400 MHz, $CDCl_3$) δ 7.57 – 7.53 (m, 2H), 7.34 – 7.31 (m, 3H), 7.23 – 7.21 (m, 1H), 7.22 – 7.18 (m, 2H), 5.16 (s, 2H), 4.45 – 4.39 (m, 1H), 4.15 (q, *J* = 7.1 Hz, 2H), 3.65 – 3.60 (m, 1H), 3.23 – 3.17 (m, 1H), 3.13 – 3.07 (m, 1H), 2.61 – 2.55 (m, 1H), 2.28 (s, 6H), 2.03 (dd, *J* = 9.6, 4.3 Hz, 1H), 1.93 (dd, *J* = 13.6, 4.1 Hz, 1H), 1.84 – 1.75 (m, 2H), 1.27 (d, *J* = 7.1 Hz, 3H). ^{13}C NMR (101 MHz, $CDCl_3$) δ 173.8, 160.1, 153.8, 148.0, 139.5, 137.6, 137.5, 137.4, 135.0, 130.0, 128.7, 128.4, 126.2, 122.6, 76.8, 60.8, 46.3, 44.7, 41.3, 40.8, 40.7, 28.1, 27.5, 19.9, 19.5, 14.2. HRMS (ESI) calcd for $C_{27}H_{31}N_4O_5$ $[M+H]^+$: 491.2294, found 491.2294. HPLC purity 98.18% (t_R =24.39 min).

*1-(4-Benzyl-2-(3,4-dimethylphenyl)-3,5-dioxo-2,3,4,5-tetrahydro-1,2,4-triazine-6-carbonyl)piperidine-3-carboxamide (**L9**)*: Compound **L9** was prepared in 46% yield from **J4** (105 mg, 0.28 mmol) and piperidine-3-carboxamide (30 mg, 0.24 mmol). The title compound was obtained as a white solid. ^{1}H NMR (300 MHz, $CDCl_3$) δ 7.43-7.30 (m, 4H), 7.29-7.20 (m, 4H), 6.98 (d, *J* = 8.1 Hz, 2H), 6.83 (t, *J* = 7.3 Hz, 1H), 5.03 (s, 2H), 3.72 (s, 4H), 3.14 (d, *J* = 19.4 Hz, 4H), 2.25 (s, 6H). ^{13}C NMR (101 MHz, $CDCl_3$) δ 174.8, 174.5, 160.6, 160.1, 154.1, 153.8, 148.0, 148.0, 139.4, 139.1, 137.6, 137.6, 137.4, 137.3, 135.0, 134.9, 130.0, 130.0, 130.0, 129.7, 128.7, 128.7,

128.4, 128.4, 126.3, 126.2, 122.7, 122.5, 49.4, 47.7, 44.8, 44.4, 43.0, 42.4, 42.0, 28.2, 27.3, 26.9, 24.6, 24.1, 19.9, 19.8, 19.5. HRMS (EI) calcd for $C_{25}H_{27}N_5O_4$ $[M]^+$: 461.2063, found 461.2067.

*1-(4-Benzyl-2-(3,4-dimethylphenyl)-3,5-dioxo-2,3,4,5-tetrahydro-1,2,4-triazine-6-carbonyl)-N-methylpiperidine-3-carboxamide (**L10**)*: Compound **L10** was prepared in 80% yield from **J4** (105 mg, 0.28 mmol) and *N*-methylpiperidine-3-carboxamide (40 mg，0.28 mmol). The title compound was obtained as a white solid. HPLC purity 100% (t_R = 11.36 min). ^{1}H NMR (300 MHz, $CDCl_3$) δ 7.60-7.48 (m, 2H), 7.37-7.29 (m, 3H), 7.26 – 7.16 (m, 3H), 6.04 (d, *J* = 5.4 Hz, 0.5H), 5.45 (s, 0.5H), 5.16 (d, *J* = 4.7 Hz, 2H), 4.60 (d, *J* = 13.5 Hz, 0.5H), 4.39 (dd, *J* = 13.7, 4.0 Hz, 0.5H), 3.67 (d, *J* = 13.6 Hz, 0.5H), 3.52 (d, *J* = 13.3 Hz, 0.5H), 3.37 – 3.19 (m, 2H), 2.83 – 2.70 (m, 3H), 2.47 – 2.39 (m, 1H), 2.29 (s, 6H), 2.01 – 1.76 (m, 3H), 1.60 (d, *J* = 12.7 Hz, 1H). ^{13}C NMR (151 MHz, $CDCl_3$) δ 173.2, 173.1, 160.3, 160.2, 154.0, 153.8, 148.0, 139.2, 137.5, 137.5, 137.4, 137.4, 135.0, 135.0, 130.0, 129.9, 129.6, 129.5, 128.6, 128.6, 128.3, 128.2, 126.3, 126.2, 122.7, 122.6, 49.4, 47.5, 44.6, 44.6, 43.5, 42.7, 42.4, 28.2, 27.4, 26.2, 26.1, 24.8, 24.2, 19.8, 19.7, 19.4. HRMS (ESI) calcd for $C_{26}H_{30}N_5O_4$ $[M+H]^+$: 476.2298, found 476.2298.

*1-(4-Benzyl-2-(3,4-dimethylphenyl)-3,5-dioxo-2,3,4,5-tetrahydro-1,2,4-triazine-6-carbonyl)-N,N-dimethylpiperidine-3-carboxamide (**L11**)*: Compound **L11** was prepared in 34% yield from **J4** (105 mg，0.28 mmol) and *N,N*-dimethylpiperidine-3-carboxamide (30.4 mg，0.24 mmol). The title compound was obtained as a white solid. ^{1}H NMR (300 MHz, $CDCl_3$) δ 7.56 – 7.43 (m, 2H), 7.34 – 7.24 (m, 3H), 7.23-7.10 (m, 3H), 5.12 (s, 2H), 4.67 (d, *J* = 12.4 Hz, 1H), 3.67 – 3.30 (m, 2H), 3.16 – 3.06 (m, 2H), 2.97 – 2.89 (m, 2H), 2.89 – 2.79 (m, 3H), 2.77 – 2.63 (m, 1H), 2.25 (s, 6H), 2.02 – 1.81 (m, 2H), 1.74 – 1.56 (m, 2H). ^{13}C NMR (151 MHz, $CDCl_3$) δ 172.7, 172.4, 1602, 160.0, 154.0, 153.9, 148.0, 139.7, 139.5, 137.6, 137.5, 137.5, 137.5, 137.4, 135.0, 134.9, 130.0, 129.9, 129.9, 129.6, 128.6, 128.4, 128.3, 126.2, 126.2, 122.6, 122.6, 49.6, 47.6, 44.7, 44.7, 42.4, 39.7, 38.9, 37.2, 36.9, 35.5, 35.4, 27.9, 27.4, 25.4, 24.4, 19.8, 19.8, 19.4. HRMS (ESI) calcd for $C_{27}H_{32}N_5O_4$ $[M+H]^+$: 490.2454, found 490.2454.

*Ethyl 1-(4-benzyl-3,5-dioxo-2-phenyl-2,3,4,5-tetrahydro-1,2,4-triazine-6-carbonyl) piperidine-3-carboxylate (**L12**)*: Compound **L12** was prepared in 64% yield from **J8** (105 mg, 0.31 mmol) and ethyl piperidine-3-carboxylate (48.3 mg, 0.31 mmol). The title compound was obtained as a white solid. HPLC purity 97.8% (t_R = 12.15 min). ^{1}H NMR (400 MHz, $CDCl_3$) δ 7.56 – 7.54 (m, 1H), 7.54 – 7.52 (m, 1H), 7.5-7.51 (m, 0.5H), 7.50 – 7.49 (m, 1H), 7.48 – 7.47 (m, 1H), 7.45 – 7.44 (m, 1H), 7.43-7.42 (m, 0.5 H), 7.41 – 7.38 (m, 1H), 7.34 – 7.30 (m, 3H), 5.17 (s, 2H), 4.71 (dd, *J* = 13.2, 4.1 Hz, 0.5H), 4.41 (d, *J* = 13.4 Hz, 0.5H), 4.18 – 4.09 (m, 2H), 3.75 (dd, *J* = 13.5, 4.0 Hz, 0.5H), 3.55 (d, *J* = 13.6 Hz, 0.5H), 3.35 (dd, *J* = 12.0, 9.0 Hz, 0.5H), 3.17 – 3.10 (m, 0.5H), 3.08 – 3.02 (m, 0.5H), 3.01-2.94 (m, 0.5H), 2.67-2.57 (m, 1H), 2.15 (s, 1H), 1.85 – 1.77 (m, 1H), 1.76 – 1.70 (m, 1H), 1.68-1.60 (m, 1H), 1.27 – 1.22 (m, 3H). ^{13}C NMR (101 MHz, $CDCl_3$) δ 172.7, 172.6, 160.1, 153.8, 153.7, 147.9, 147.9, 139.9, 139.7, 139.7, 139.6, 134.9, 134.9, 130.0, 129.9, 129.0, 128.9, 128.7, 128.7, 128.6, 128.4, 128.4, 125.2, 125.1, 60.9, 60.9, 48.7, 47.4, 44.8, 44.7, 43.8, 42.4, 41.7, 41.1, 27.4, 27.3, 24.8, 23.9, 14.2. HRMS (EI) calcd for $C_{25}H_{26}N_4O_5$ $[M]^+$: 462.1903, found 462.1901.

*Ethyl 1-(4-benzyl-3,5-dioxo-2-(m-tolyl)-2,3,4,5-tetrahydro-1,2,4-triazine-6-carbonyl)piperidine-3-carboxylate (**L13**)*: Compound **L13** was prepared in 57% yield from **J2** (105 mg, 0.30 mmol) and ethyl piperidine-3-carboxylate (45 mg, 0.29 mmol). The title compound was obtained as a white solid. ^{1}H NMR (300 MHz, $CDCl_3$) δ 7.56 – 7.49 (m, 2H), 7.35 –7.19 (m, 7H), 5.16 (s, 2H), 4.71 (d, *J* = 13.4 Hz, 0.5H), 4.41 (d, *J* = 13.3 Hz, 0.5H), 4.22 - 4.03 (m, 2H), 3.74 (d, *J* = 13.7 Hz, 0.5H), 3.54 (d, *J* = 13.6 Hz, 0.5H), 3.34 (t, *J* = 12.1 Hz, 0.5H), 3.20 – 2.91 (m, 1.5H), 2.62 (s, 1H), 2.39 s, 3H), 2.15 (s, 1H), 1.80 -1.65 (m, 3H), 1.31 – 1.18 (m, 3H). ^{13}C NMR (101 MHz, $CDCl_3$) δ 172.8, 158.1, 140.6, 140.4, 139.6, 138.9, 138.1, 132.8, 132.1, 131.6, 131.0, 127.2, 125.5, 123.6, 123.0, 122.5, 122.1, 116.7, 60.8, 49.3, 48.1, 44.4, 43.0, 41.9, 41.3, 27.6, 27.4, 25.0, 24.3, 19.9, 19.6, 14.2. HRMS (EI) calcd for $C_{26}H_{28}N_4O_5$ $[M]^+$: 476.2060, found 476.2057.

*Ethyl 1-(4-benzyl-3,5-dioxo-2-(p-tolyl)-2,3,4,5-tetrahydro-1,2,4-triazine-6-carbonyl)piperidine-3-carboxylate (**L14**)*: Compound **L14** was prepared in 57% yield

from **J3** (105 mg, 0.30 mmol) and ethyl-piperidine-3-carboxylate (45 mg, 0.29 mmol). The title compound was obtained as a white solid. ^{1}H NMR (300 MHz, $CDCl_3$) δ 7.54 (s, 2H), 7.41 – 7.30 (m, 4H), 7.27 – 7.21 (m, 3H), 5.16 (s, 2H), 4.71 (d, *J* = 13.2 Hz, 0.5H), 4.42 (d, *J* = 13.3 Hz, 0.5H), 4.14 (s, 2H), 3.75 (d, *J* = 14.2 Hz, 0.5H), 3.55 (d, *J* = 12.0 Hz, 0.5H), 3.33 (t, *J* = 11.8 Hz, 0.5H), 3.22 – 2.90 (m, 1.5H), 2.62 (s, 1H), 2.39 (d, *J* = 3.8 Hz, 3H), 2.15 (s, 1H), 1.73 (s, 3H), 1.33 – 1.24 (m, 3H). ^{13}C NMR (151 MHz, $CDCl_3$) δ 172.7, 172.6, 160.2, 160.1, 153.8, 153.7, 148.0, 139.6, 139.3, 138.7, 137.2, 137.2, 135.0, 134.9, 130.0, 129.9, 129.5, 129.5, 128.7, 128.6, 128.4, 128.3, 125.0, 124.9, 60.9, 60.8, 48.7, 47.4, 44.7, 44.7, 43.8, 42.4, 41.7, 41.1, 27.4, 27.3, 24.8, 23.9, 21.1, 14.2. HRMS (EI) calcd for $C_{26}H_{28}N_4O_5$ $[M]^+$: 476.2060, found 476.2064. HPLC purity 99.37% (t_R = 24.31 min).

*Ethyl 1-(2-(3,4-dimethylphenyl)-4-(3-fluorobenzyl)-3,5-dioxo-2,3,4,5-tetrahydro-1,2,4-triazine-6-carbonyl) piperidine-3-carboxylate (**L15**)*: Compound **L15** was prepared in 63% yield from **J9** (100 mg, 0.27 mmol) and ethyl piperidine-3-carboxylate (39 mg, 0.25 mmol). The title compound was obtained as a white solid. ^{1}H NMR (400 MHz, $CDCl_3$) δ 7.32 – 7.27 (m, 2H), 7.26 – 7.17 (m, 4H), 7.02 – 6.96 (m, 1H), 5.14 (s, 2H), 4.70 (ddd, *J* = 13.3, 3.9, 1.8 Hz, 0.5H), 4.40 – 4.35 (m, 0.5H), 4.19 – 4.10 (m, 2H), 3.74 (dt, *J* = 13.8, 2.6 Hz, 0.5H), 3.55 (d, *J* = 13.7 Hz, 0.5H), 3.37 (dd, *J* = 13.7, 10.0 Hz, 0.5H), 3.17 – 2.98 (m, 1.5H), 2.65 – 2.58 (m, 1H), 2.28 (s, 6H), 2.18 – 2.12 (m, 1H), 1.85 – 1.59 (m, 3H), 1.29 – 1.20 (m, 3H). ^{13}C NMR (151 MHz, $CDCl_3$) δ 172.7, 172.6, 162.7 (d, *J* = 246.9 Hz), 160.1, 153.7, 153.6, 147.9, 147.9, 139.4, 139.2, 137.6 (t, *J* = 3.5 Hz), 137.3, 137.2, 137.1, 137.0, 130.2, 130.1 (d, *J* = 3.0 Hz), 130.1, 130.0, 130.0, 126.2, 126.1, 125.6 (d, *J* = 3.2 Hz), 125.5 (d, *J* = 3.6 Hz), 122.6, 122.5, 117.0 (d, *J* = 3.1 Hz), 116.8 (d, *J* = 3.3 Hz), 115.5, 115.4, 115.3, 115.3, 60.9, 60.8, 48.6, 47.4, 44.1, 43.8, 42.4, 41.6, 41.0, 27.3, 27.2, 24.8, 23.9, 19.8, 19.8, 19.4, 14.1, 14.1.HRMS (ESI) calcd for $C_{27}H_{29}FN_4O_5$ $[M]^+$:508.2122, found 508.2129. HPLC purity 99.98% (t_R = 24.59 min).

*Ethyl 1-(2-(3,4-dimethylphenyl)-4-(4-fluorobenzyl)-3,5-dioxo-2,3,4,5-tetrahydro-1,2,4-triazine-6-carbonyl) piperidine-3-carboxylate (**L16**)*: Compound **L16** was prepared in 9.5% yield from **J10** (113 mg, 0.29 mmol) and ethyl-piperidine-3-

carboxylate (46 mg, 0.29 mmol). The title compound was obtained as a white solid. 1H NMR (300 MHz, $CDCl_3$) δ 7.53 (t, *J* = 6.6 Hz, 2H), 7.22 (d, *J* = 7.7 Hz, 2H), 7.19 (s, 1H), 7.04 – 6.93 (m, 2H), 5.12 (s, 2H), 4.70 (dd, *J* = 13.4, 4.1 Hz, 0.5H), 4.40 (d, J = 13.4 Hz, 0.5H), 4.13 (dq, *J* = 16.7, 5.2, 3.3 Hz, 2H), 3.73 (dd, *J* = 13.7, 4.0 Hz,0.5H), 3.55 (d, *J* = 13.5 Hz, 0.5H), 3.33 (dd, *J* = 13.7, 10.1 Hz, 0.5H), 3.18 – 2.91 (m, 1.5H), 2.61 (ddt, *J* = 10.6, 7.2, 4.3 Hz, 1H), 2.28 (s, 6H), 2.16 (d, *J* = 12.6 Hz, 1H), 1.85 – 1.70 (m, 3H), 1.36 – 1.17 (m, 3H). ^{13}C NMR (151 MHz, $CDCl_3$) δ 172.8, 172.7, 162.8 (d, *J* = 247.4 Hz), 160.3, 153.9, 153.8, 148.1, 139.6, 139.4, 137.7, 137.4 (d, *J* = 6.3 Hz), 132.2, 132.1, 132.1, 130.9, 130.1, 130.1, 126.3, 126.2, 122.7, 122.6, 115.7 (d, *J* = 4.1 Hz), 115.6 (d, *J* = 3.6 Hz), 61.1, 61.0, 48.8, 47.5, 44.1, 43.9, 42.6, 41.8, 41.2, 27.5, 27.4, 24.9, 24.1, 20.0, 19.9, 19.6, 14.3, 14.3. HRMS (ESI) calcd for $C_{27}H_{29}FN_4O_5Na$ $[M+Na]^+$:531.2020, found 531.2015.

Spectra of compounds D2-D4, I1-I7, J4 and L1-L16

D2

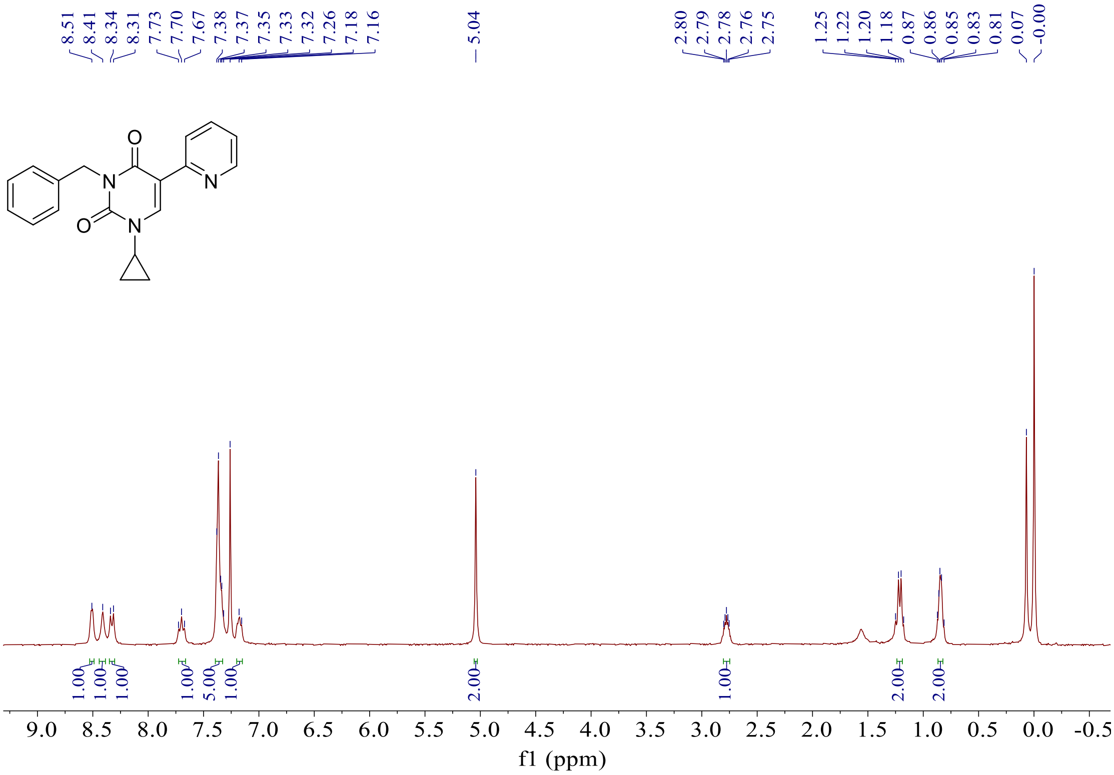

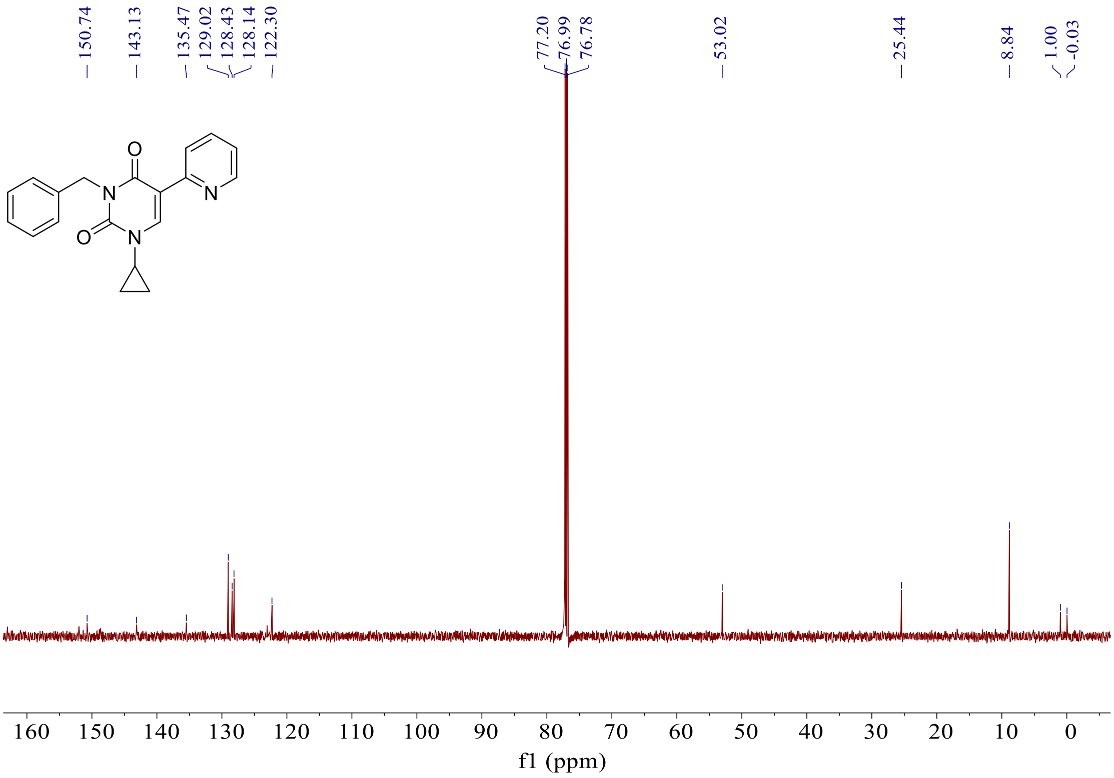

D3

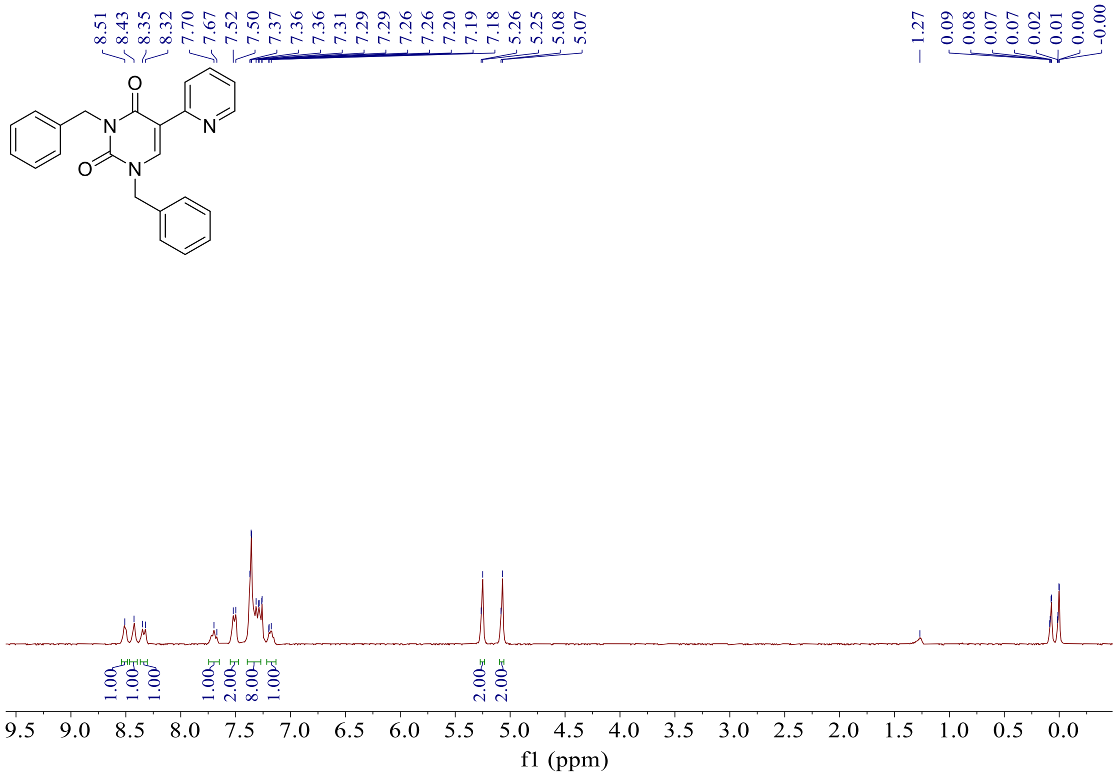
8.51
8.43
8.35
8.32
7.70
7.67
7.52
7.50
7.37
7.36
7.36
7.31
7.29
7.29
7.26
7.26
7.20
7.19
7.18
5.26
5.25
5.08
5.07
1.27
0.09
0.08
0.07
0.07
0.02
0.01
0.00
-0.00
O
N
N
O
N
1.00
1.00
1.00
1.00
2.00
8.00
1.00
2.00
2.00
9.5 9.0 8.5 8.0 7.5 7.0 6.5 6.0 5.5 5.0 4.5 4.0 3.5 3.0 2.5 2.0 1.5 1.0 0.5 0.0
f1 (ppm)

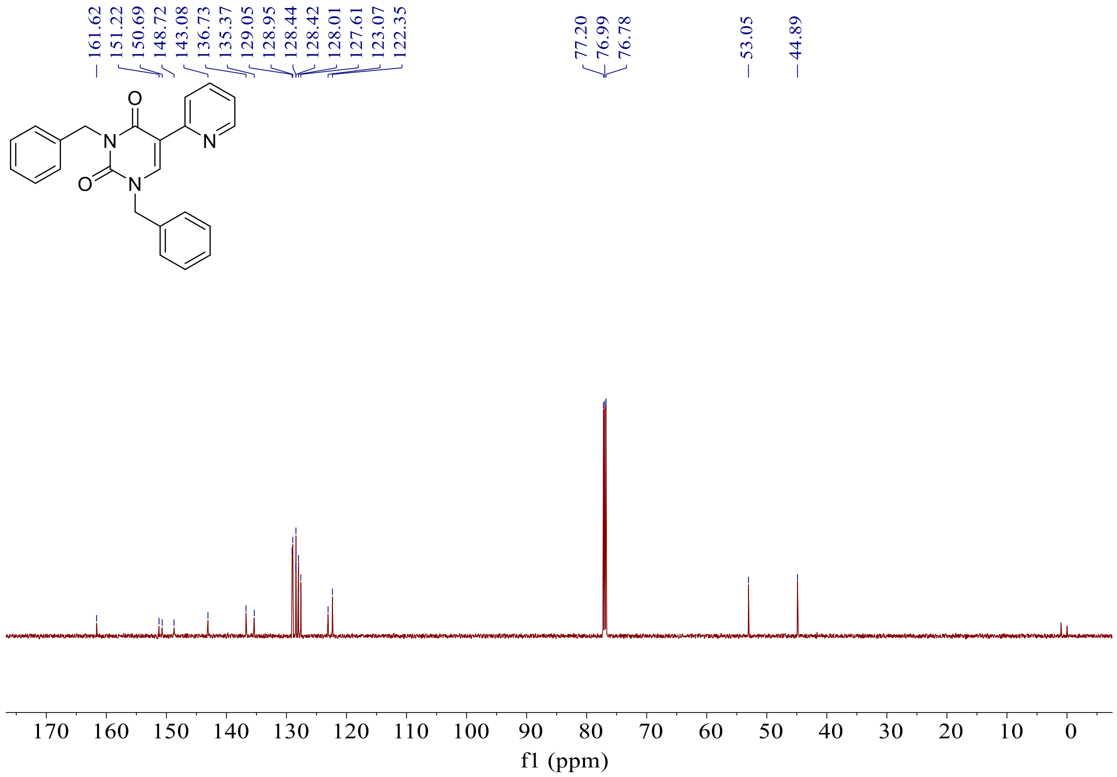
161.62
151.22
150.69
148.72
143.08
136.73
135.37
129.05
128.95
128.44
128.42
128.01
127.61
123.07
122.35
77.20
76.99
76.78
53.05
44.89
O
N
N
O
N
170 160 150 140 130 120 110 100 90 80 70 60 50 40 30 20 10 0
f1 (ppm)

D4

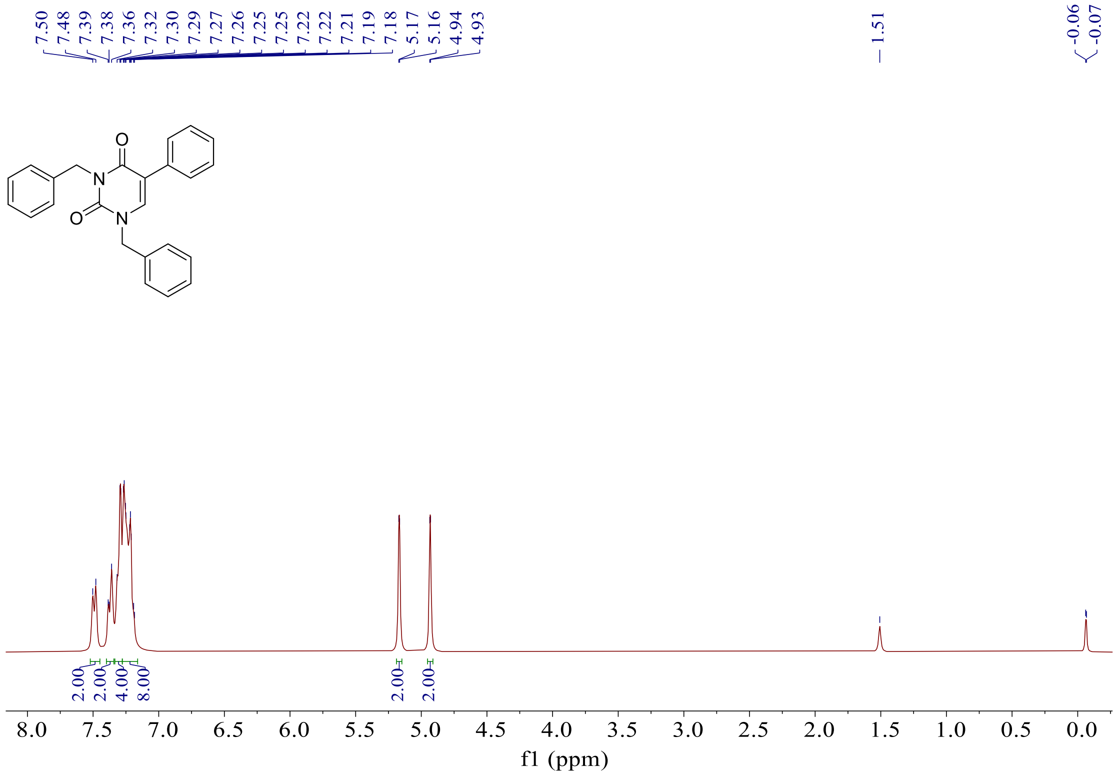
7.50 7.48 7.39 7.38 7.36 7.32 7.30 7.29 7.27 7.26 7.25 7.25 7.22 7.22 7.21 7.19 7.18 5.17 5.16 4.94 4.93
1.51
-0.06 -0.07
O
N
O
N
2.00 2.00 4.00 8.00
2.00 2.00
8.0 7.5 7.0 6.5 6.0 5.5 5.0 4.5 4.0 3.5 3.0 2.5 2.0 1.5 1.0 0.5 0.0
f1 (ppm)

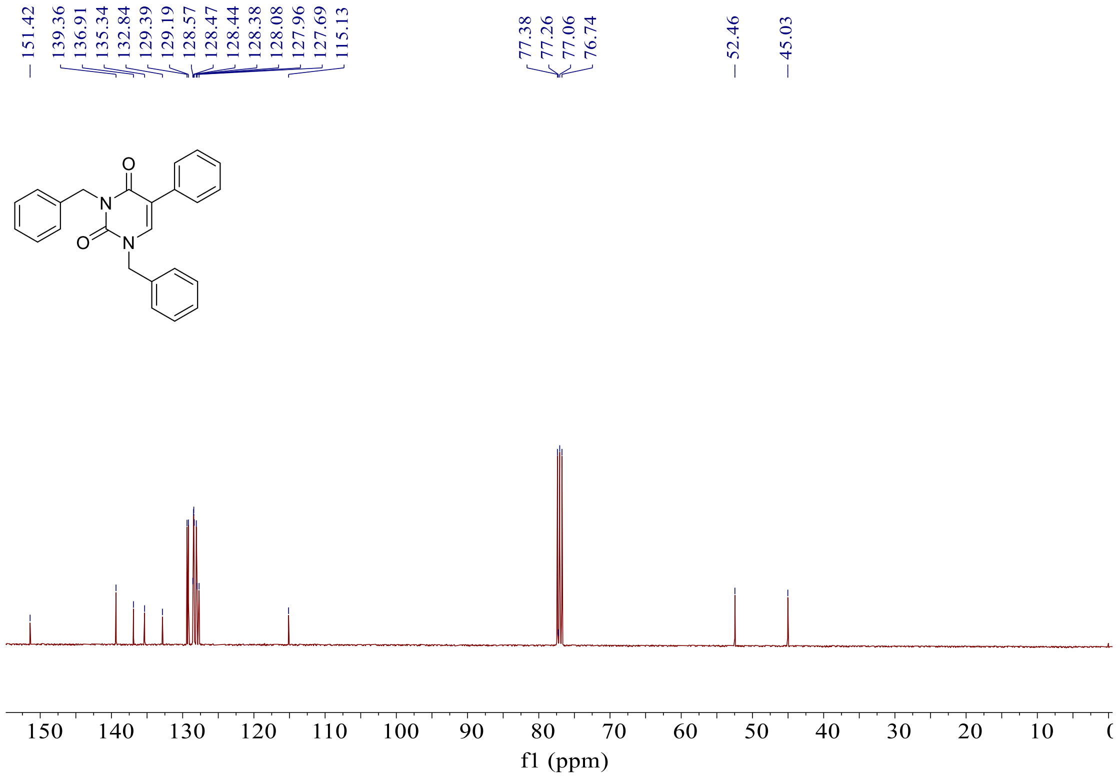
151.42
139.36 136.91 135.34 132.84 129.39 129.19 128.57 128.47 128.44 128.38 128.08 127.96 127.69 115.13
77.38 77.26 77.06 76.74
52.46
45.03
O
N
O
N
150 140 130 120 110 100 90 80 70 60 50 40 30 20 10
f1 (ppm)

I1

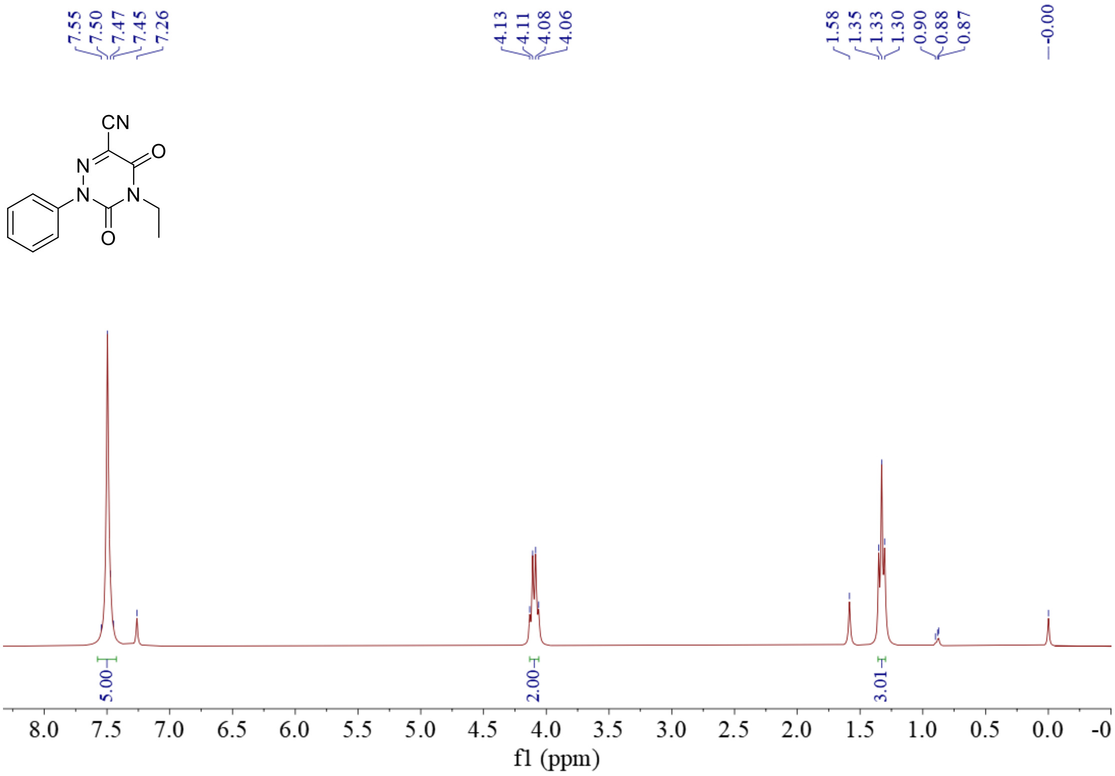
7.55
7.50
7.47
7.45
7.26
4.13
4.11
4.08
4.06
1.58
1.35
1.33
1.30
0.90
0.88
0.87
-0.00
CN
N
N
N
O
O
5.00
2.00
3.01
8.0 7.5 7.0 6.5 6.0 5.5 5.0 4.5 4.0 3.5 3.0 2.5 2.0 1.5 1.0 0.5 0.0 -0
f1 (ppm)

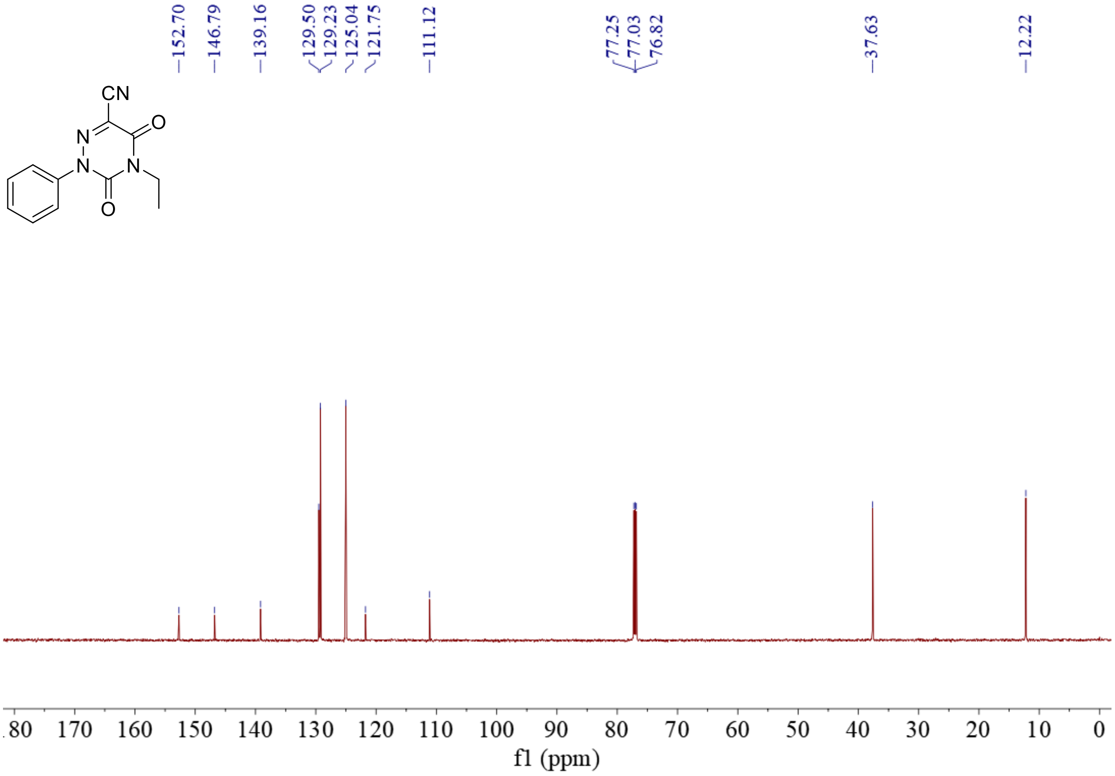
152.70
146.79
139.16
129.50
129.23
125.04
121.75
111.12
77.25
77.03
76.82
37.63
12.22
CN
N
N
N
O
O
180 170 160 150 140 130 120 110 100 90 80 70 60 50 40 30 20 10 0
f1 (ppm)

I2

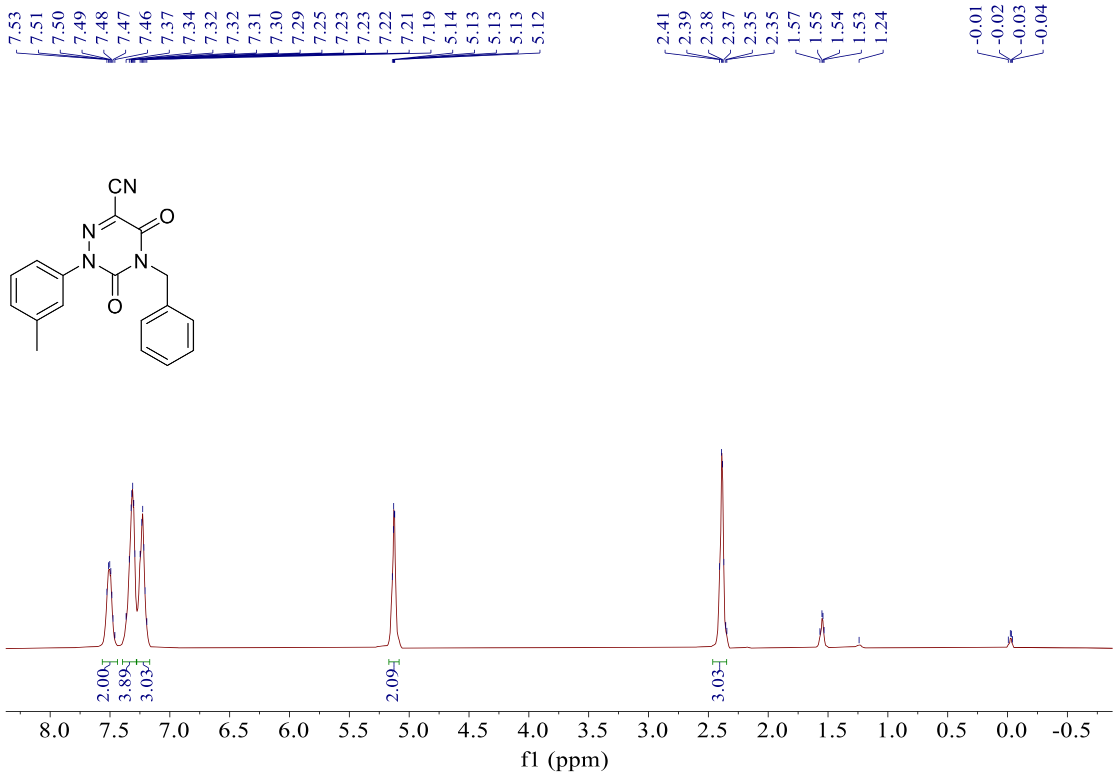
7.53 7.51 7.50 7.49 7.48 7.47 7.46 7.37 7.34 7.32 7.32 7.31 7.30 7.29 7.25 7.23 7.23 7.22 7.21 7.19 5.14 5.13 5.13 5.13 5.12
2.41 2.39 2.38 2.37 2.35 2.35 1.57 1.55 1.54 1.53 1.24
-0.01 -0.02 -0.03 -0.04
CN
N
N
N
O
O
2.00 3.89 3.03
2.09
3.03
8.0 7.5 7.0 6.5 6.0 5.5 5.0 4.5 4.0 3.5 3.0 2.5 2.0 1.5 1.0 0.5 0.0 -0.5
f1 (ppm)

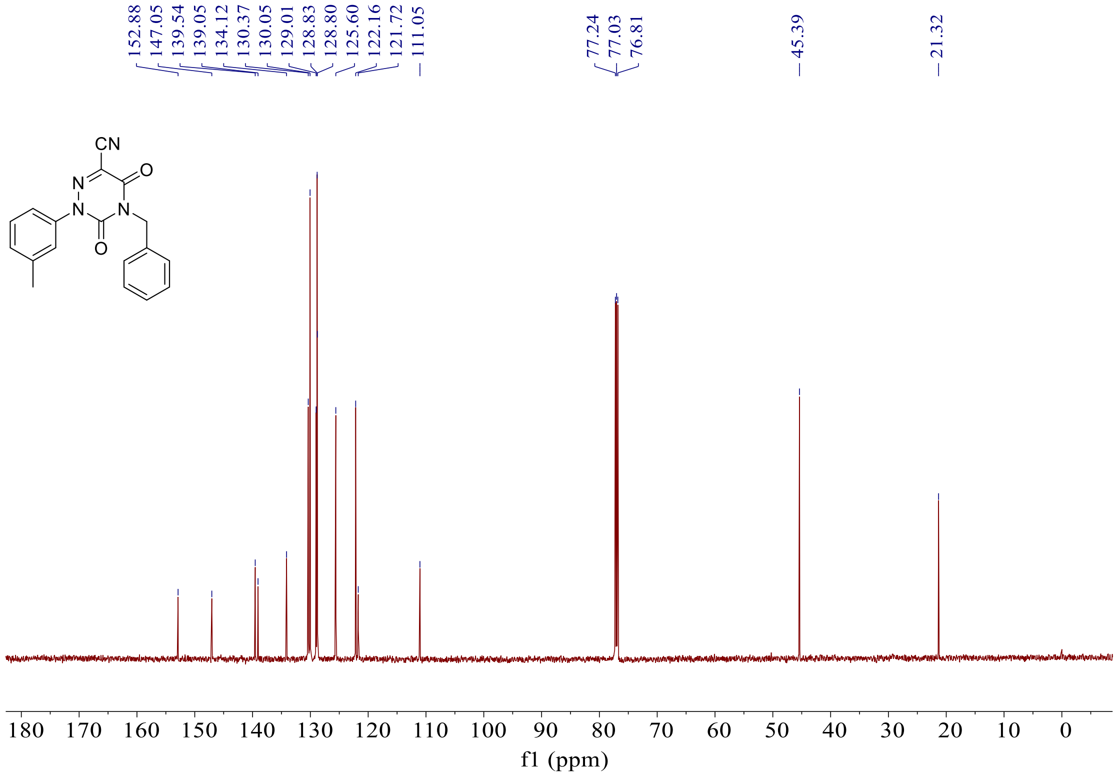
152.88 147.05 139.54 139.05 134.12 130.37 130.05 129.01 128.83 128.80 125.60 122.16 121.72 111.05
77.24 77.03 76.81
45.39
21.32
CN
N
N
N
O
O
180 170 160 150 140 130 120 110 100 90 80 70 60 50 40 30 20 10 0
f1 (ppm)

I3

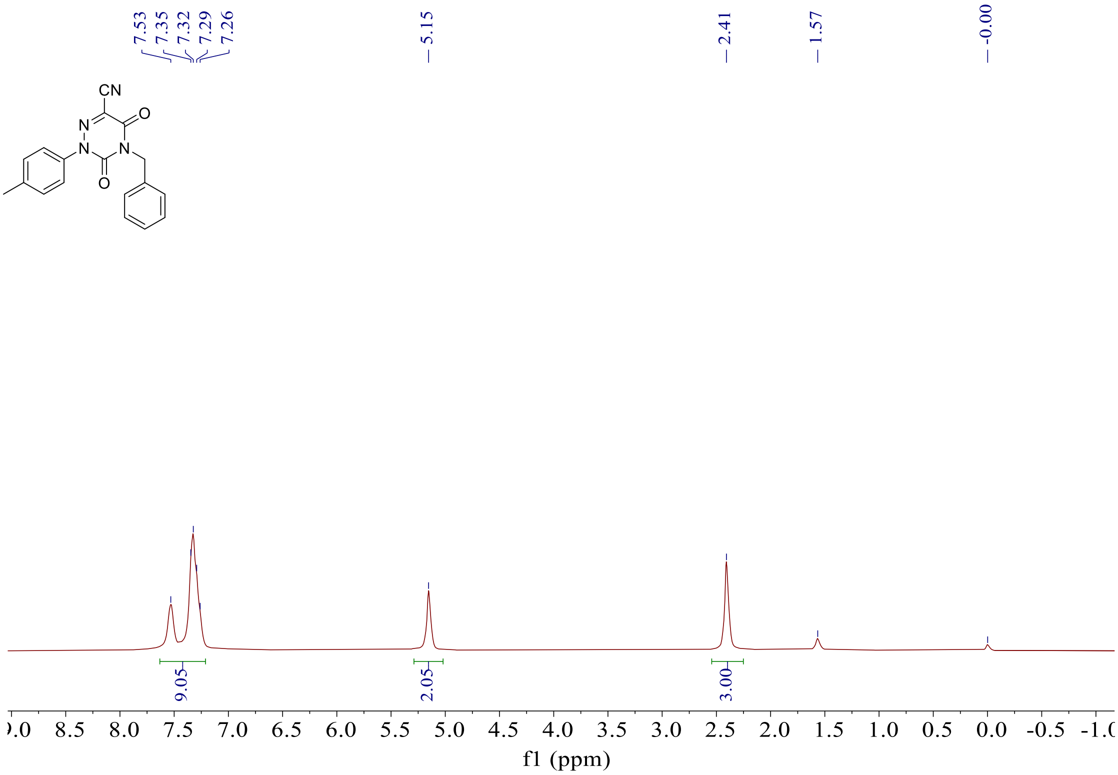
7.53
7.35
7.32
7.29
7.26
5.15
2.41
1.57
-0.00
CN
N
N
N
O
O
9.05
2.05
3.00
f1 (ppm)

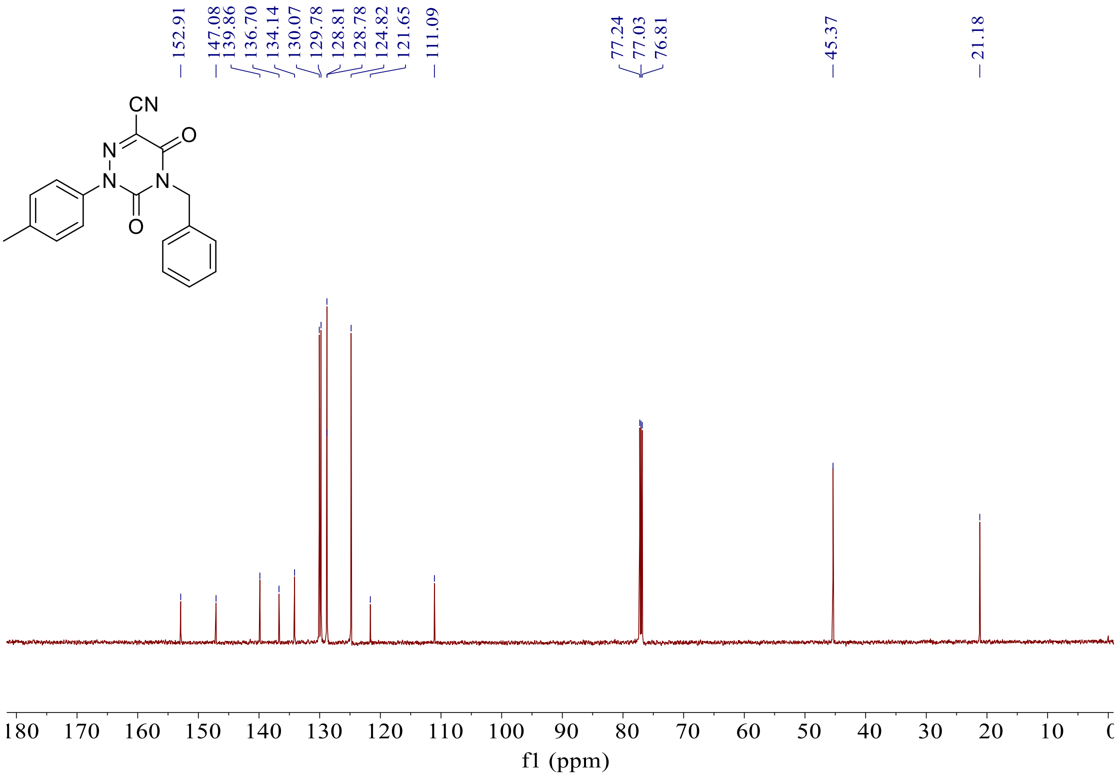
152.91
147.08
139.86
136.70
134.14
130.07
129.78
128.81
128.78
124.82
121.65
111.09
77.24
77.03
76.81
45.37
21.18
CN
N
N
N
O
O
f1 (ppm)

I4

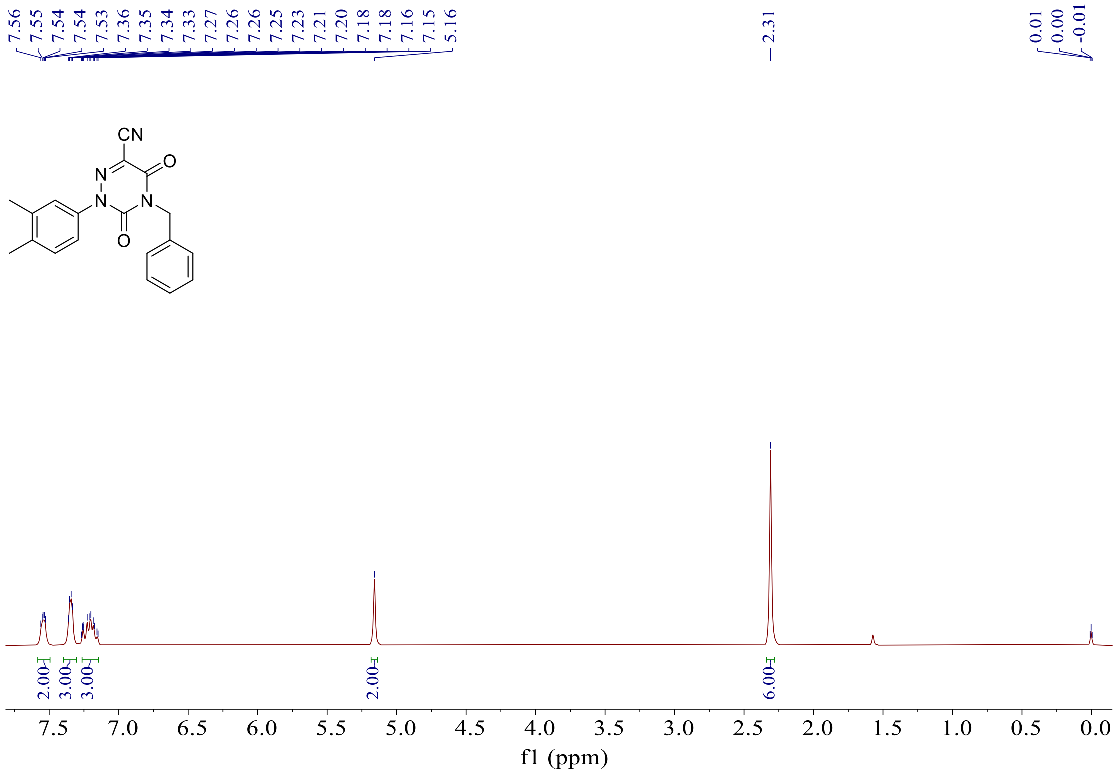
7.56
7.55
7.54
7.54
7.53
7.36
7.35
7.34
7.33
7.27
7.26
7.26
7.25
7.23
7.21
7.20
7.18
7.18
7.16
7.15
5.16
2.31
0.01
0.00
-0.01
CN
N
N
N
O
O
2.00
3.00
3.00
2.00
6.00
7.5
7.0
6.5
6.0
5.5
5.0
4.5
4.0
3.5
3.0
2.5
2.0
1.5
1.0
0.5
0.0
f1 (ppm)

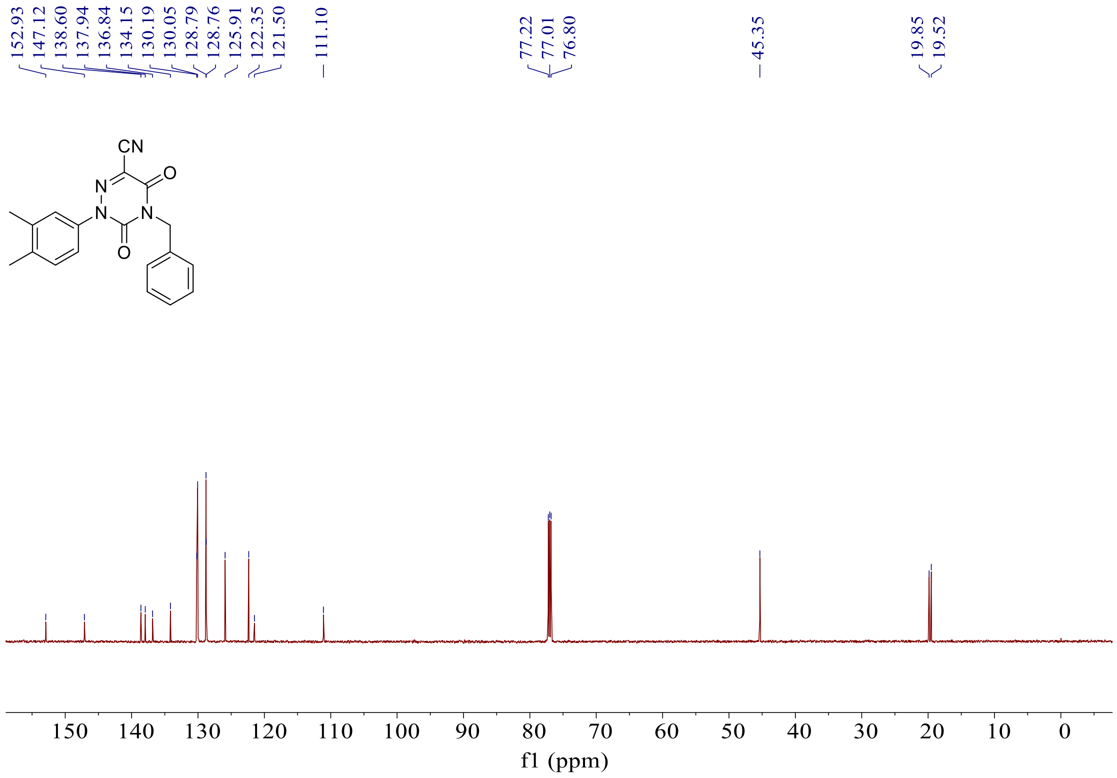
152.93
147.12
138.60
137.94
136.84
134.15
130.19
130.05
128.79
128.76
125.91
122.35
121.50
111.10
77.22
77.01
76.80
45.35
19.85
19.52
CN
N
N
N
O
O
150
140
130
120
110
100
90
80
70
60
50
40
30
20
10
0
f1 (ppm)

I5

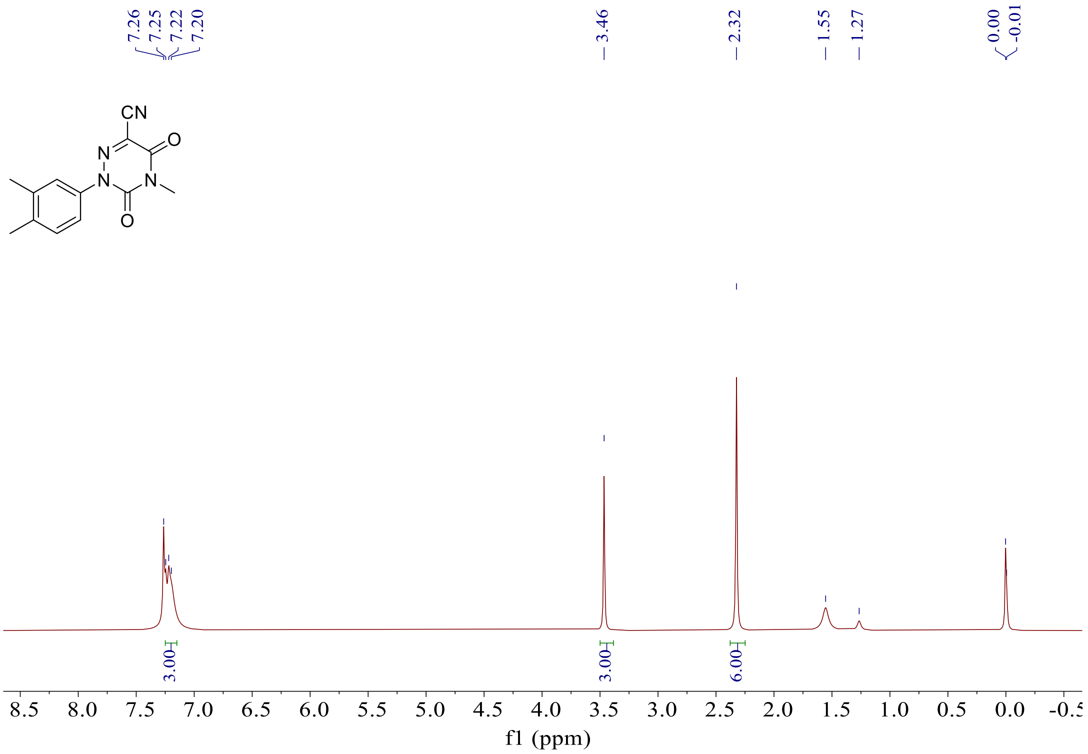

7.26
7.25
7.22
7.20
3.46
2.32
1.55
1.27
0.00
-0.01
CN
N
N
N
O
O
3.00
3.00
6.00
8.5 8.0 7.5 7.0 6.5 6.0 5.5 5.0 4.5 4.0 3.5 3.0 2.5 2.0 1.5 1.0 0.5 0.0
f1 (ppm)

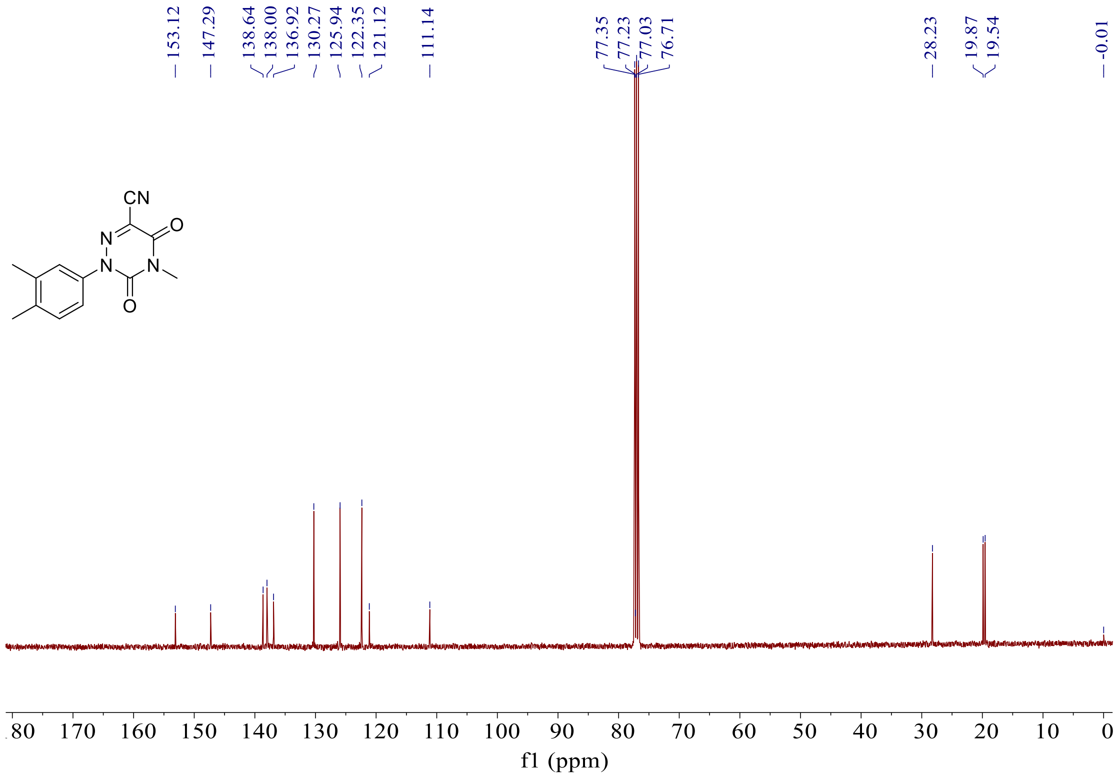

153.12
147.29
138.64
138.00
136.92
130.27
125.94
122.35
121.12
111.14
77.35
77.23
77.03
76.71
28.23
19.87
19.54
-0.01
CN
N
N
N
O
O
170 160 150 140 130 120 110 100 90 80 70 60 50 40 30 20 10 0
f1 (ppm)

I6

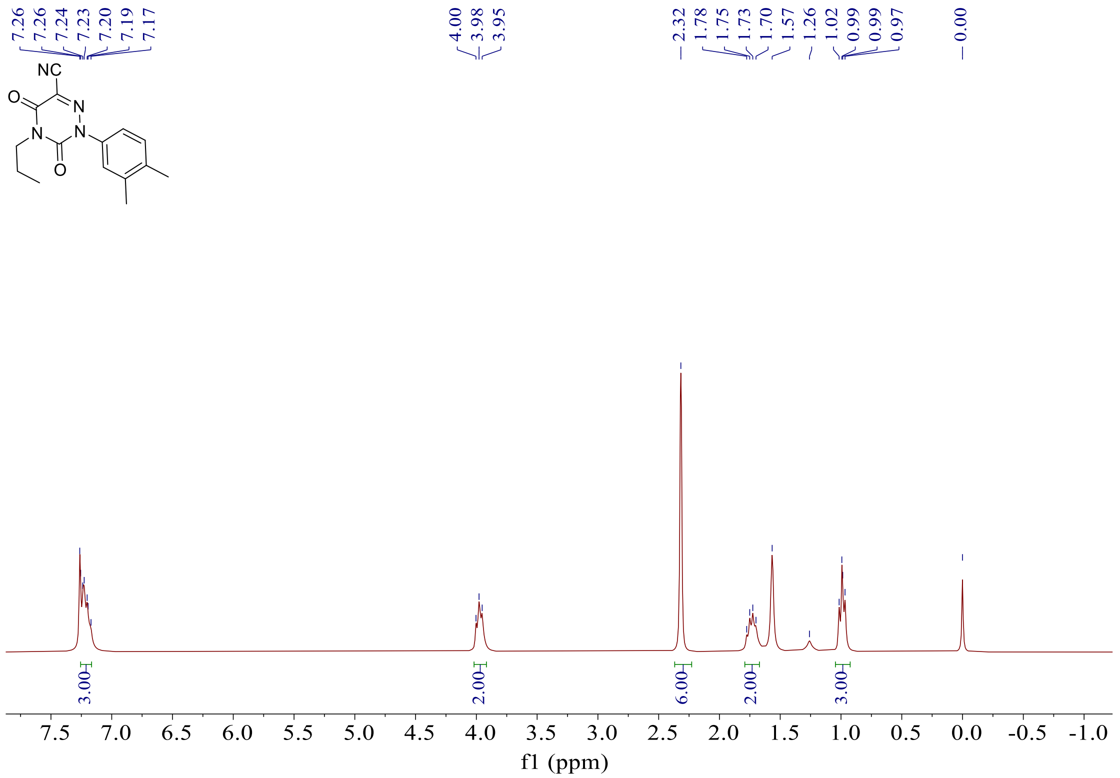

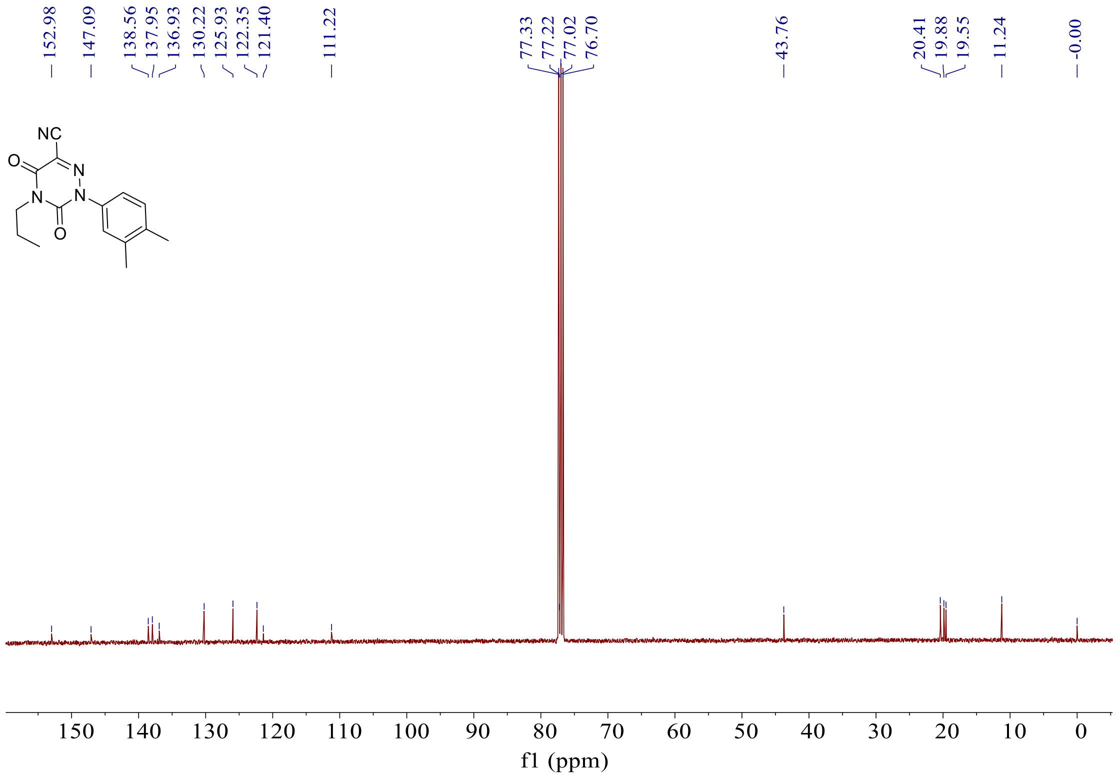

I7

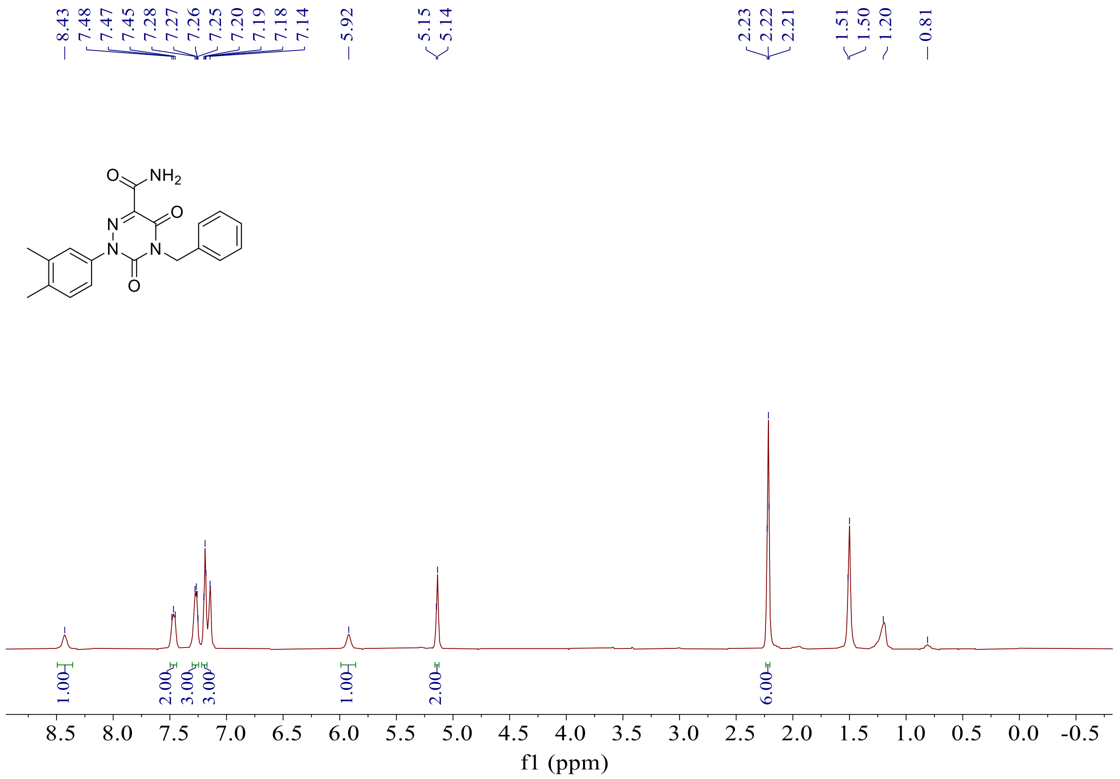

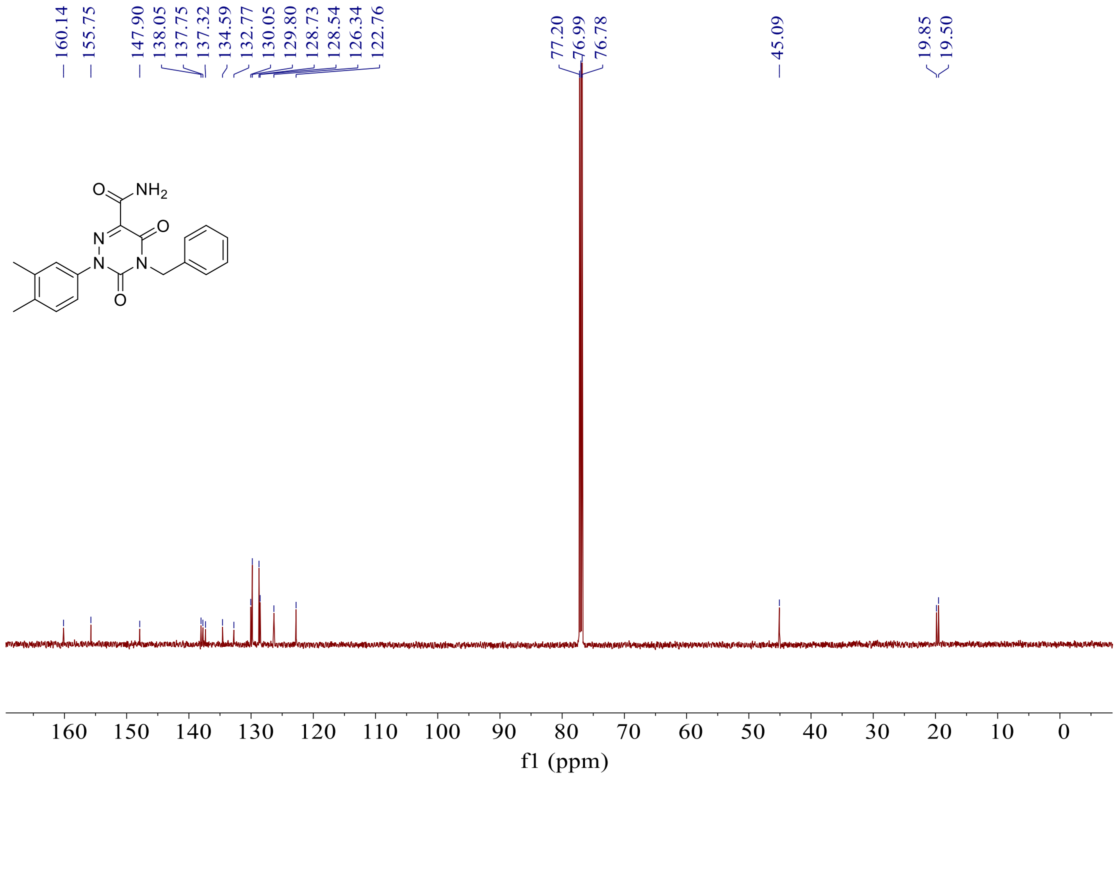

I9

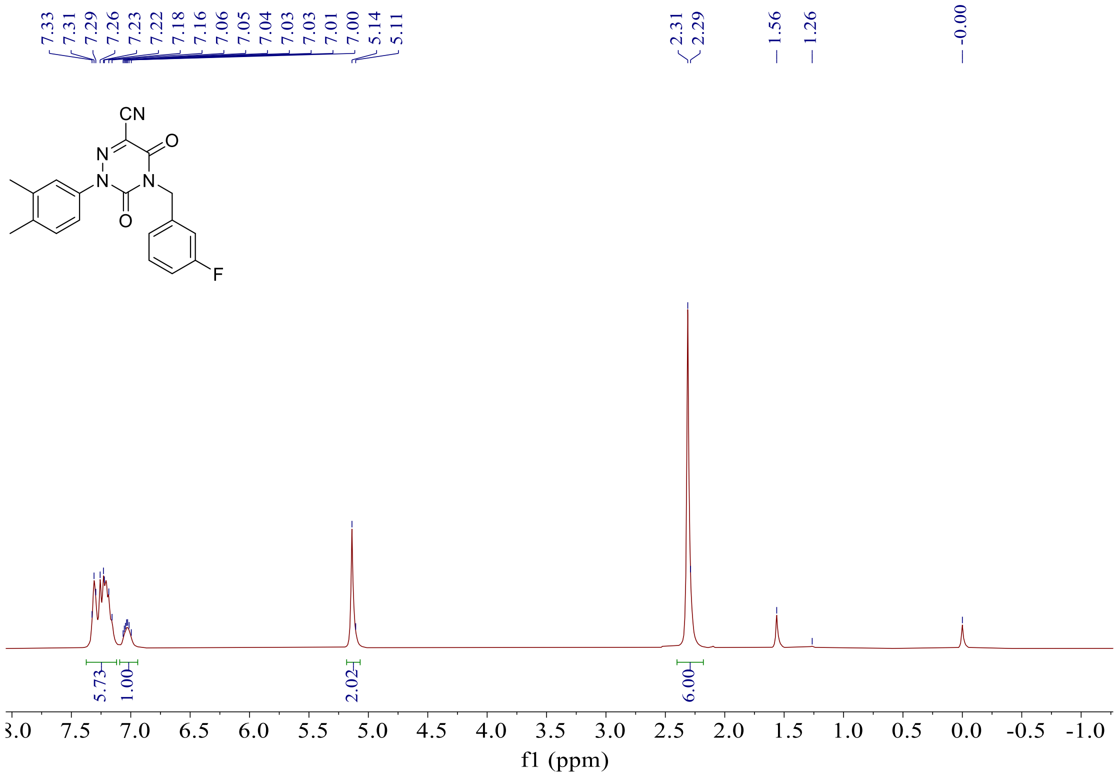

7.33 7.31 7.29 7.26 7.23 7.22 7.18 7.16 7.06 7.05 7.04 7.03 7.03 7.01 7.00 5.14 5.11
2.31 2.29
1.56
1.26
-0.00
CN
N
N
N
O
O
F
5.73
1.00
2.02
6.00
f1 (ppm)

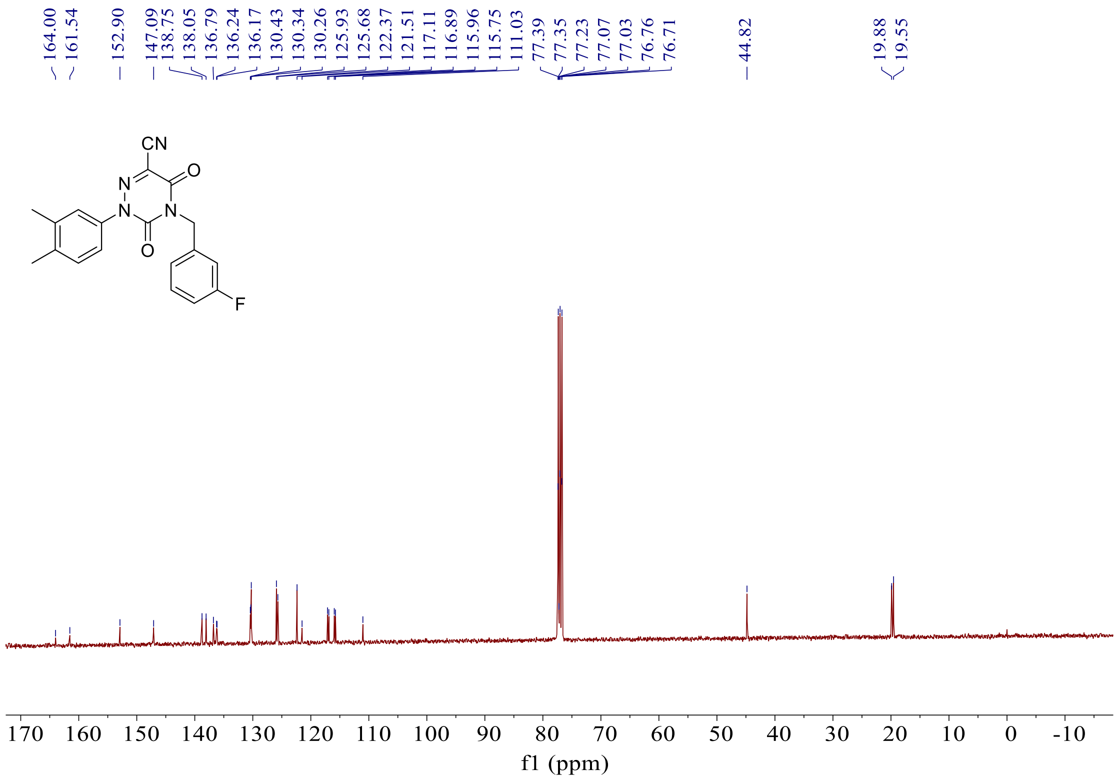

164.00 161.54
152.90
147.09 138.75 138.05 136.79 136.24 136.17 130.43 130.34 130.26 125.93 125.68 122.37 121.51 117.11 116.89 115.96 115.75 111.03
77.39 77.35 77.23 77.07 77.03 76.76 76.71
44.82
19.88 19.55
CN
N
N
N
O
O
F
f1 (ppm)

I10

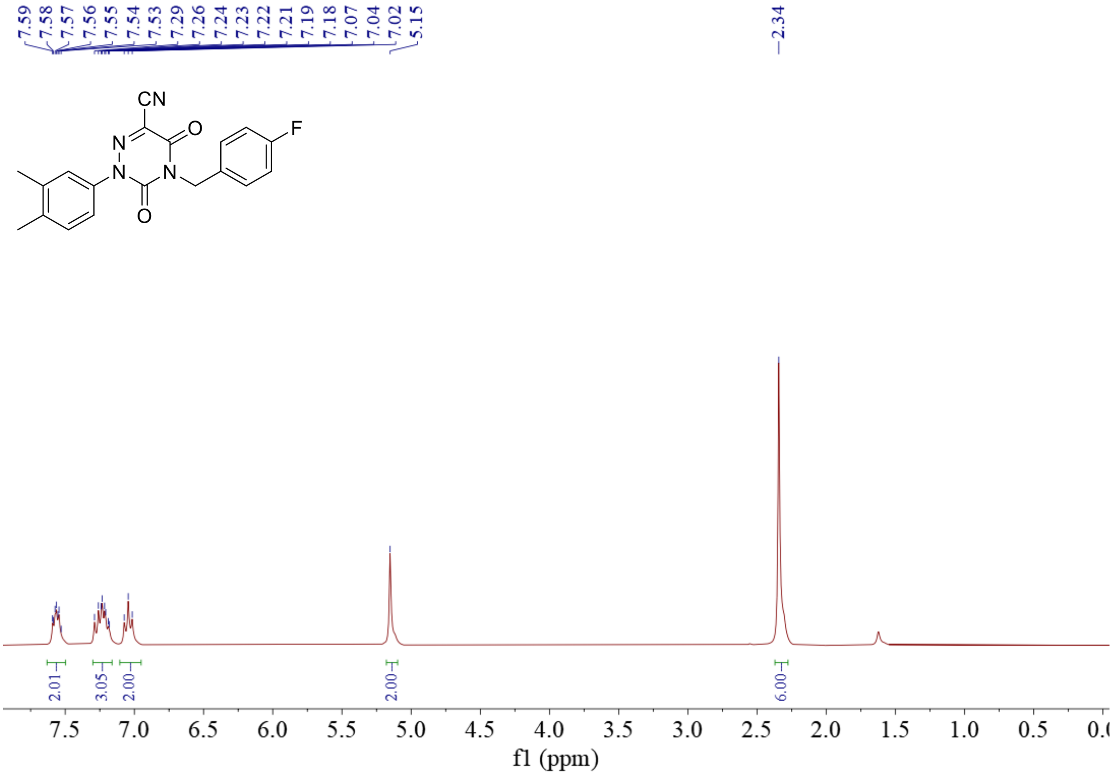
7.59
7.58
7.57
7.56
7.55
7.54
7.53
7.29
7.26
7.24
7.23
7.22
7.21
7.19
7.18
7.07
7.04
7.02
5.15
2.34
CN
N
N
N
O
O
F
2.01
3.05
2.00
2.00
6.00
7.5
7.0
6.5
6.0
5.5
5.0
4.5
4.0
3.5
3.0
2.5
2.0
1.5
1.0
0.5
f1 (ppm)

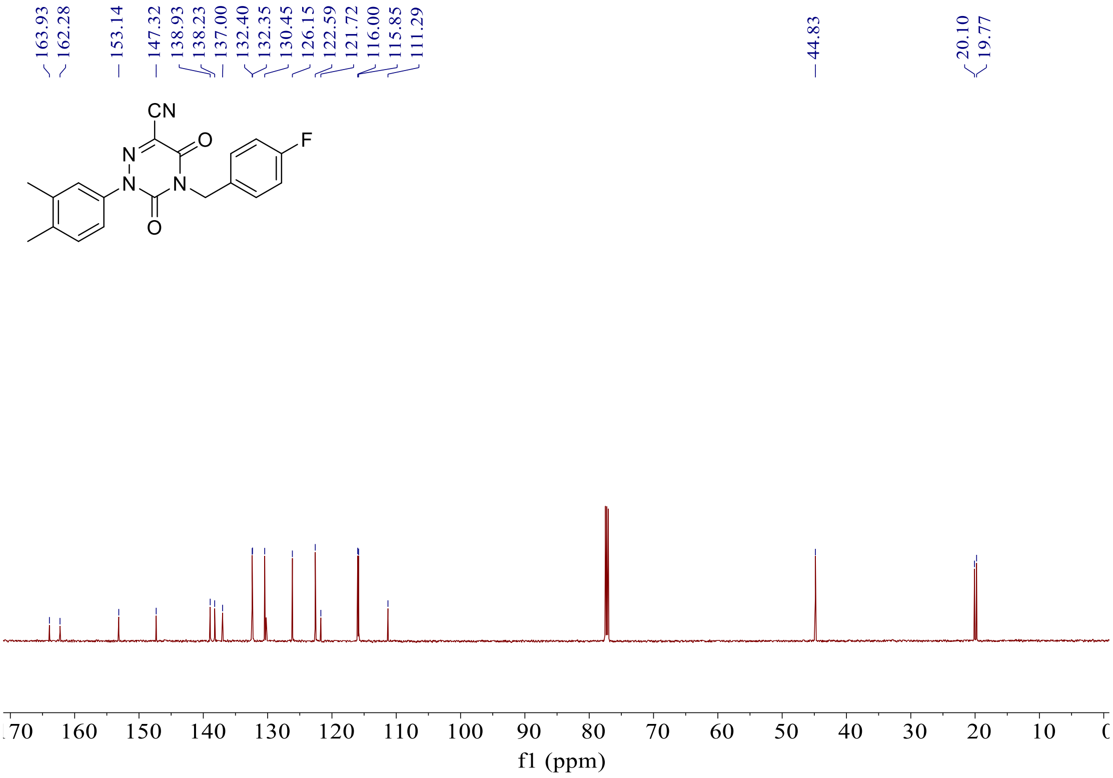
163.93
162.28
153.14
147.32
138.93
138.23
137.00
132.40
132.35
130.45
126.15
122.59
121.72
116.00
115.85
111.29
44.83
20.10
19.77
CN
N
N
N
O
O
F
160
150
140
130
120
110
100
90
80
70
60
50
40
30
20
10
f1 (ppm)

J4

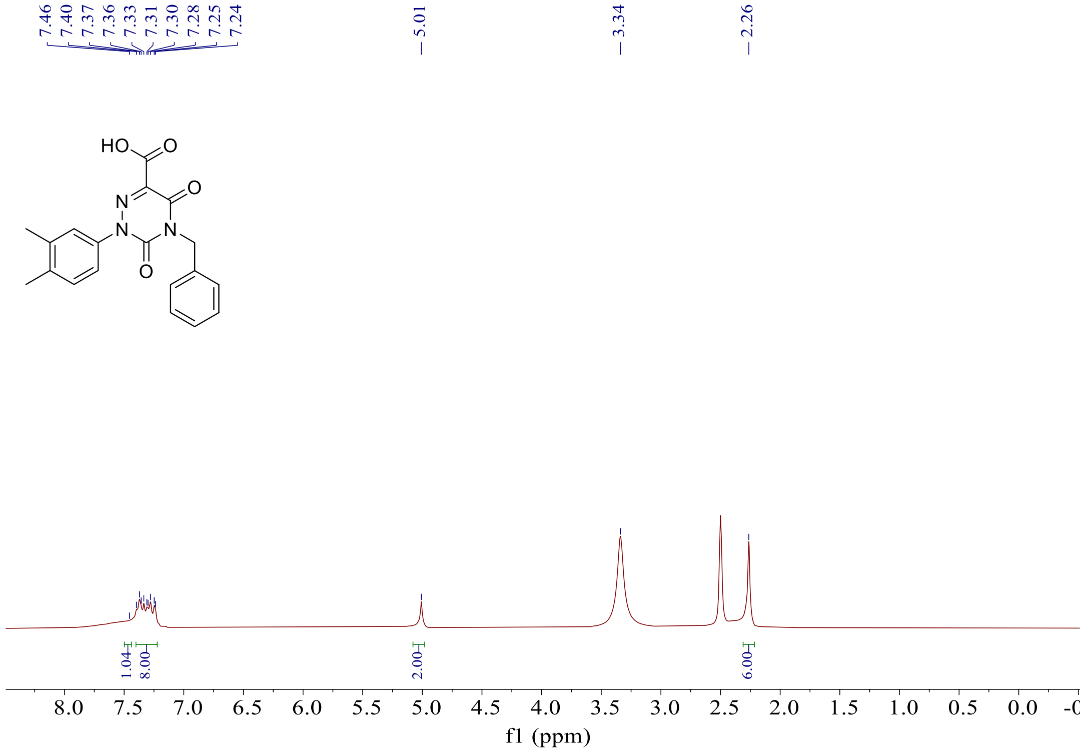
7.46
7.40
7.37
7.36
7.33
7.31
7.30
7.28
7.25
7.24
5.01
3.34
2.26
HO
O
O
N
N
N
O
1.04
8.00
2.00
6.00
8.0 7.5 7.0 6.5 6.0 5.5 5.0 4.5 4.0 3.5 3.0 2.5 2.0 1.5 1.0 0.5 0.0
f1 (ppm)

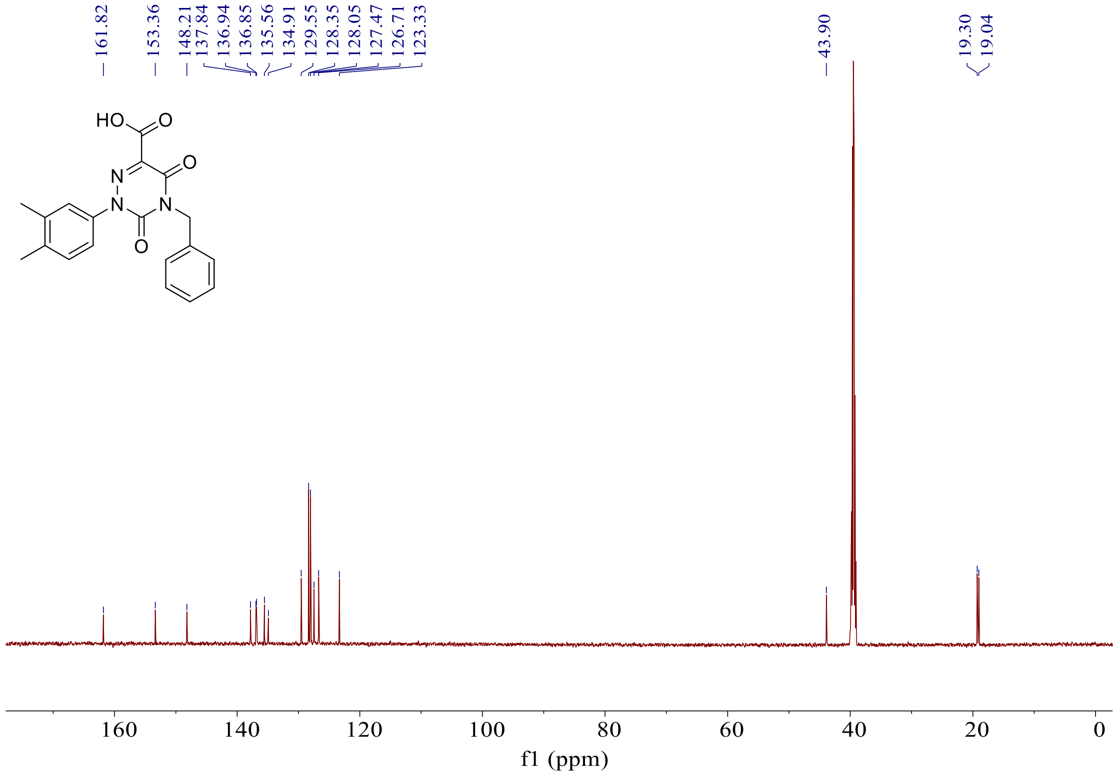
161.82
153.36
148.21
137.84
136.94
136.85
135.56
134.91
129.55
128.35
128.05
127.47
126.71
123.33
43.90
19.30
19.04
HO
O
O
N
N
N
O
160 140 120 100 80 60 40 20 0
f1 (ppm)

L1

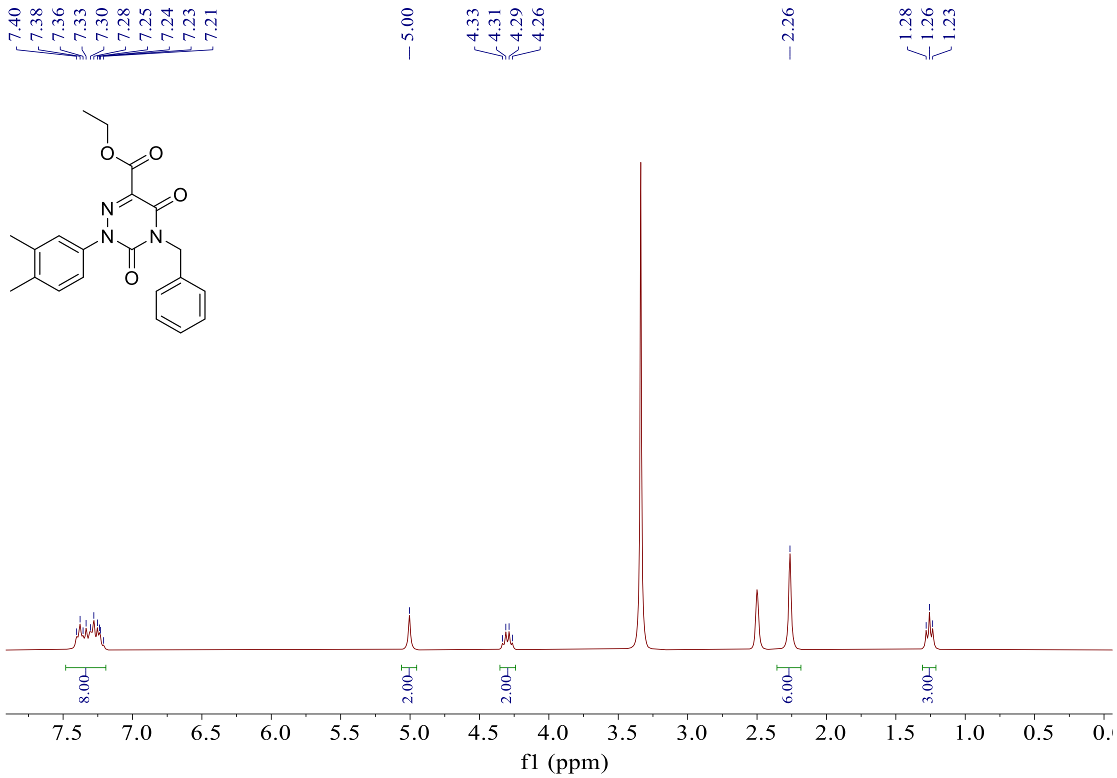
7.40 7.38 7.36 7.33 7.30 7.28 7.25 7.24 7.23 7.21
5.00
4.33 4.31 4.29 4.26
2.26
1.28 1.26 1.23
8.00
2.00
2.00
6.00
3.00
7.5 7.0 6.5 6.0 5.5 5.0 4.5 4.0 3.5 3.0 2.5 2.0 1.5 1.0 0.5
f1 (ppm)

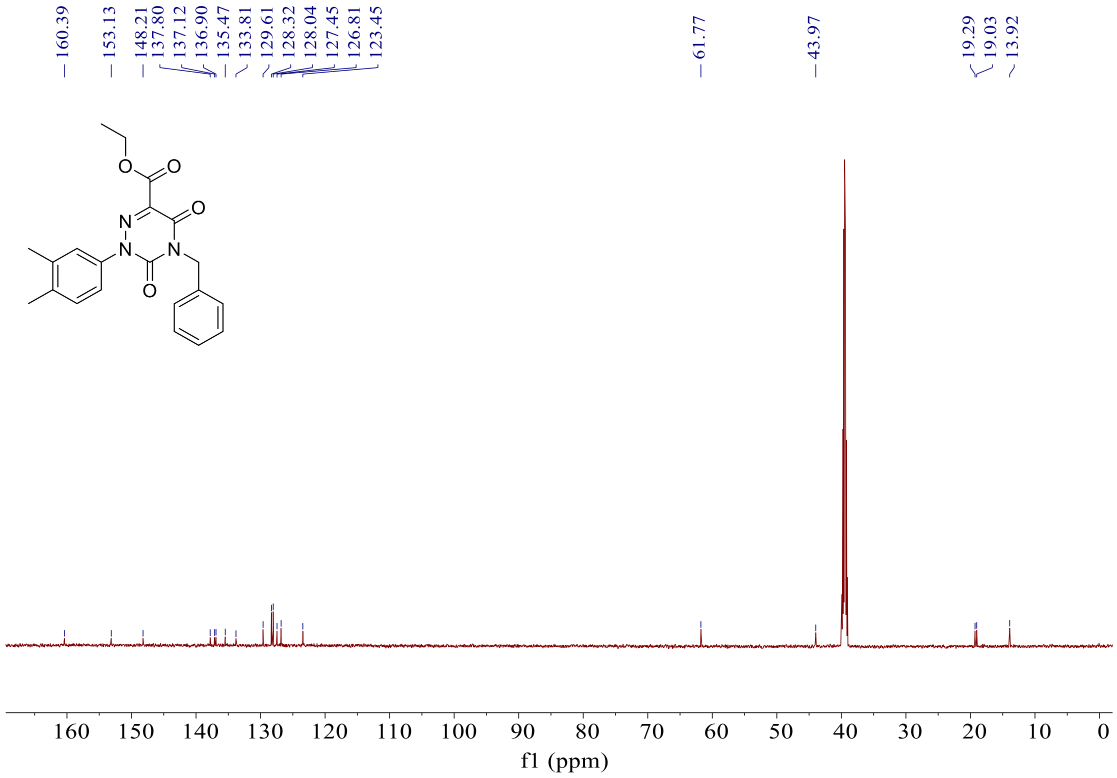
160.39
153.13
148.21 137.80 137.12 136.90 135.47 133.81 129.61 128.32 128.04 127.45 126.81 123.45
61.77
43.97
19.29 19.03 13.92
160 150 140 130 120 110 100 90 80 70 60 50 40 30 20 10 0
f1 (ppm)

L2

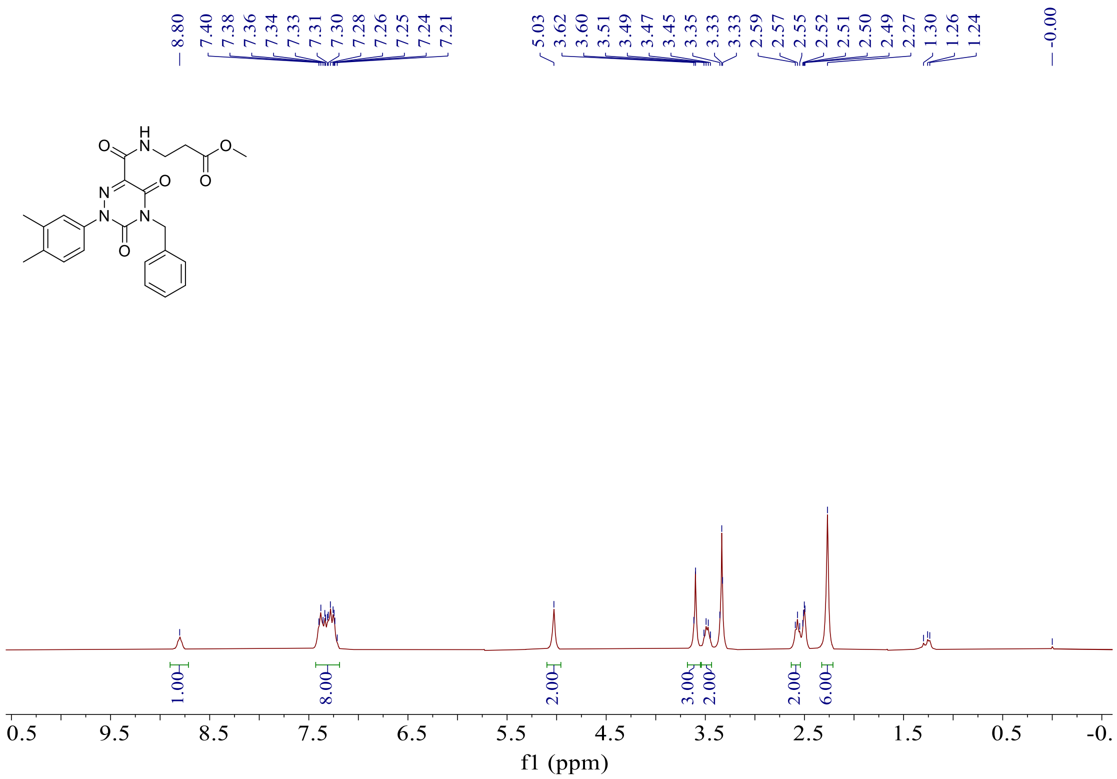
8.80 7.40 7.38 7.36 7.34 7.33 7.31 7.30 7.28 7.26 7.25 7.24 7.21 5.03 3.62 3.60 3.51 3.49 3.47 3.45 3.35 3.33 3.33 2.59 2.57 2.55 2.52 2.51 2.50 2.49 2.27 1.30 1.26 1.24 -0.00
1.00 8.00 2.00 3.00 2.00 2.00 6.00
f1 (ppm)

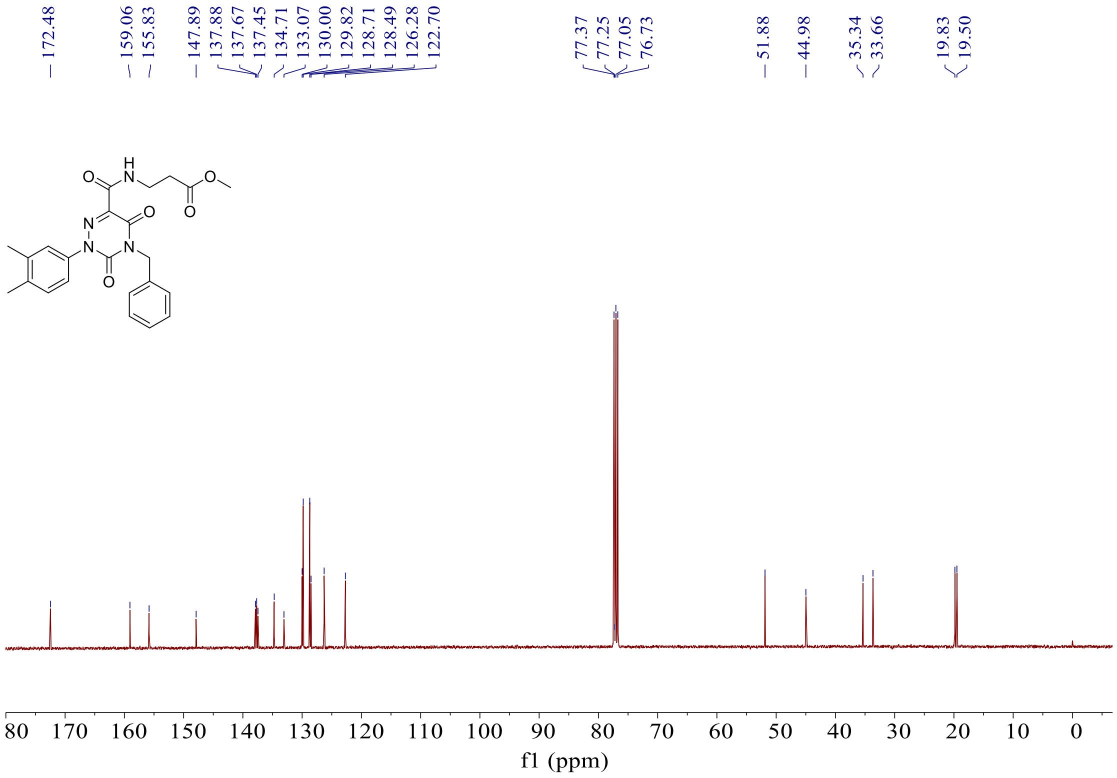
172.48 159.06 155.83 147.89 137.88 137.67 137.45 134.71 133.07 130.00 129.82 128.71 128.49 126.28 122.70 77.37 77.25 77.05 76.73 51.88 44.98 35.34 33.66 19.83 19.50
f1 (ppm)

L3

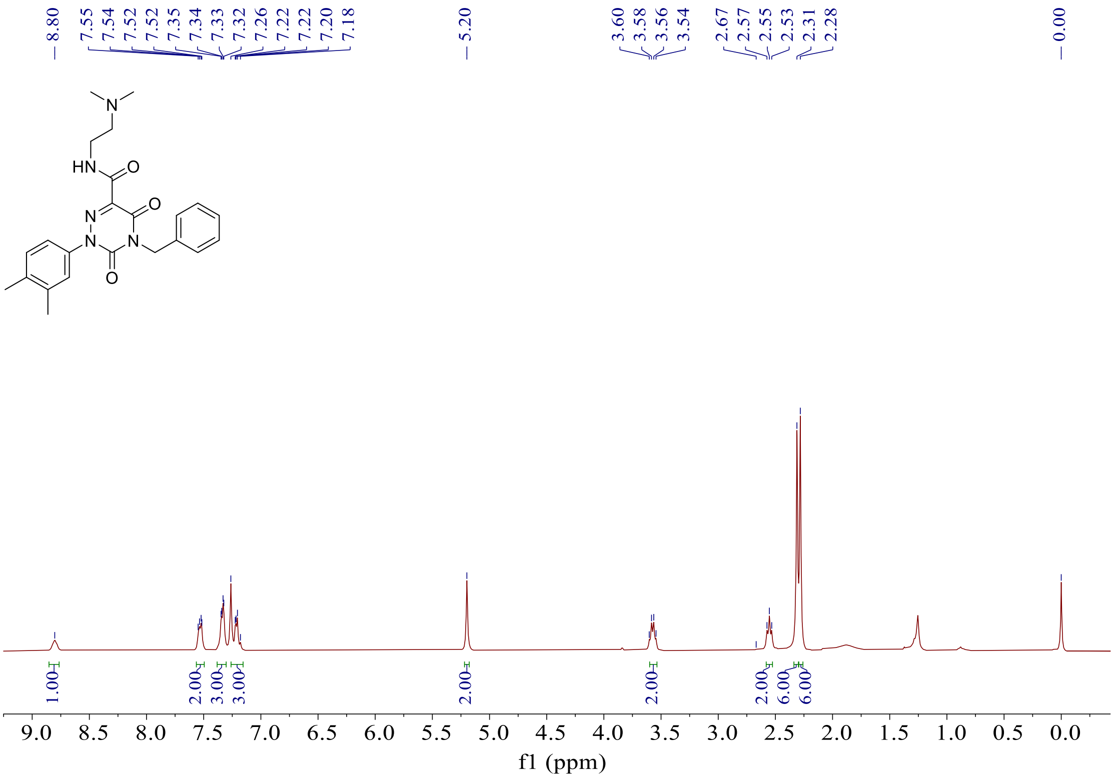
8.80
7.55 7.54 7.52 7.52 7.35 7.34 7.33 7.32 7.26 7.22 7.22 7.20 7.18
5.20
3.60 3.58 3.56 3.54
2.67 2.57 2.55 2.53 2.31 2.28
0.00
1.00 2.00 3.00 3.00 2.00 2.00 2.00 6.00 6.00
9.0 8.5 8.0 7.5 7.0 6.5 6.0 5.5 5.0 4.5 4.0 3.5 3.0 2.5 2.0 1.5 1.0 0.5 0.0
f1 (ppm)

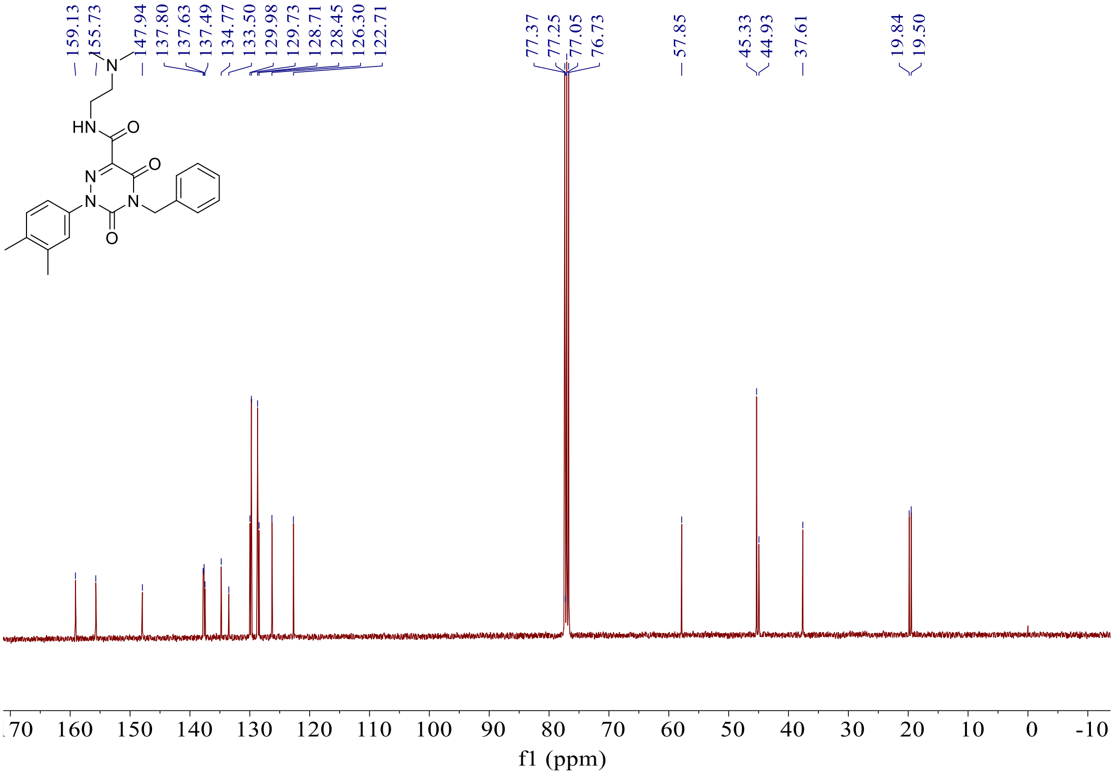
159.13 155.73 147.94 137.80 137.63 137.49 134.77 133.50 129.98 129.73 128.71 128.45 126.30 122.71
77.37 77.25 77.05 76.73
57.85
45.33 44.93
37.61
19.84 19.50
170 160 150 140 130 120 110 100 90 80 70 60 50 40 30 20 10 0 -10
f1 (ppm)

L4

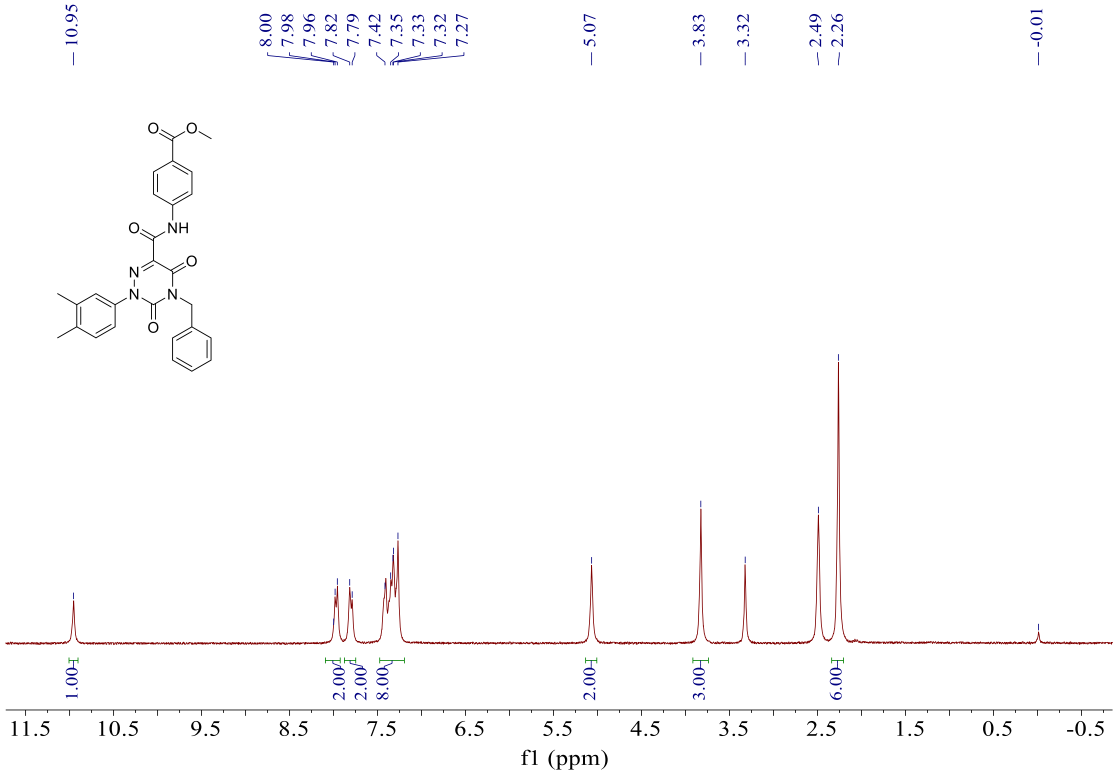
10.95
8.00
7.98
7.96
7.82
7.79
7.42
7.35
7.33
7.32
7.27
5.07
3.83
3.32
2.49
2.26
-0.01
O
O
NH
O
N
N
N
O
O
1.00
2.00
2.00
8.00
2.00
3.00
6.00
11.5
10.5
9.5
8.5
7.5
6.5
5.5
4.5
3.5
2.5
1.5
0.5
-0.5
f1 (ppm)

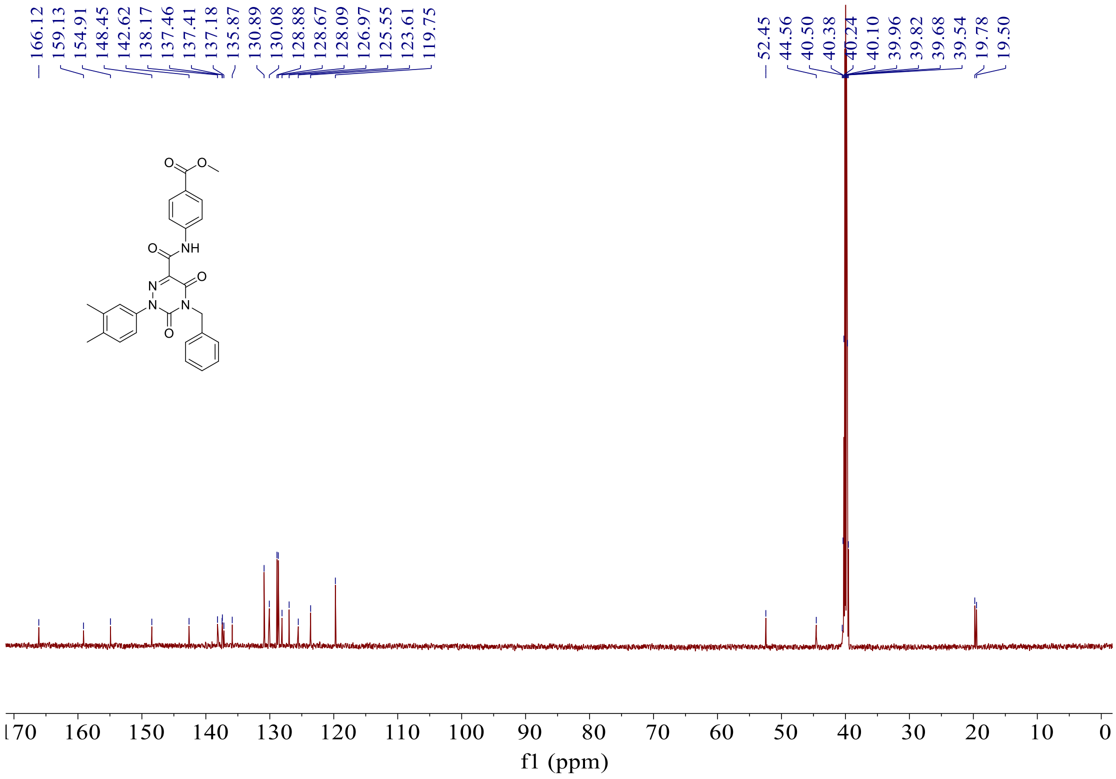
166.12
159.13
154.91
148.45
142.62
138.17
137.46
137.41
137.18
135.87
130.89
130.08
128.88
128.67
128.09
126.97
125.55
123.61
119.75
52.45
44.56
40.50
40.38
40.24
40.10
39.96
39.82
39.68
39.54
19.78
19.50
170
160
150
140
130
120
110
100
90
80
70
60
50
40
30
20
10
0
f1 (ppm)

L5

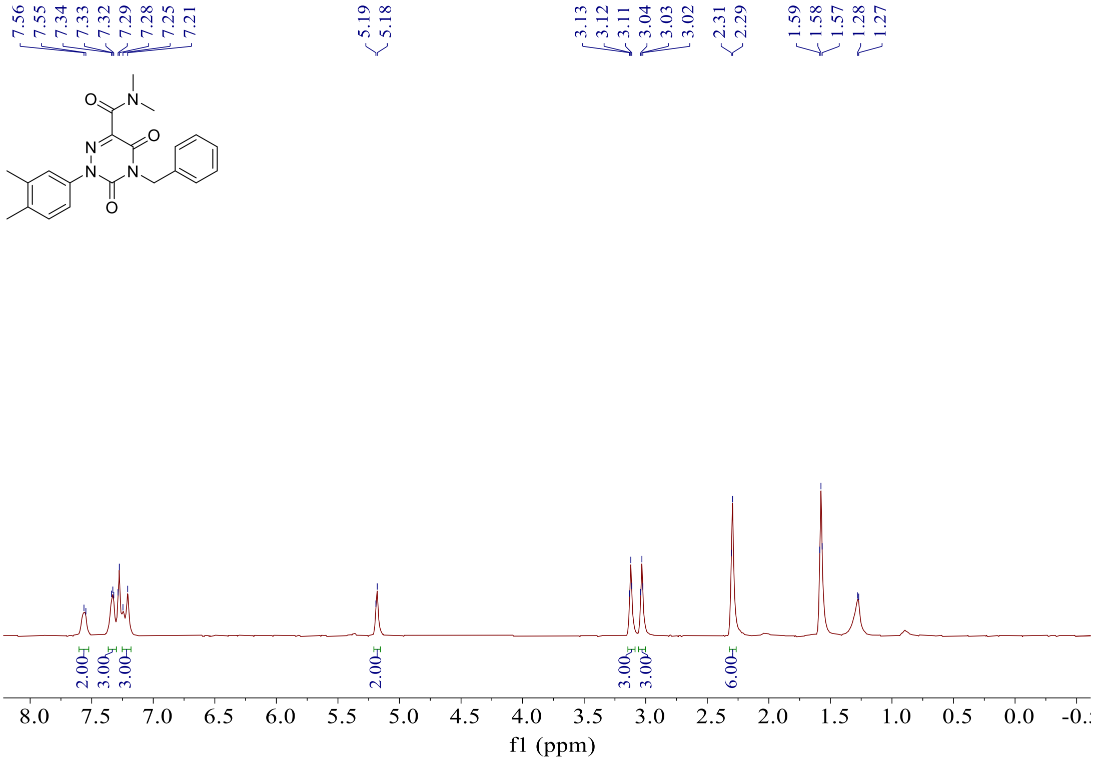
7.56 7.55 7.34 7.33 7.32 7.29 7.28 7.25 7.21
5.19 5.18
3.13 3.12 3.11 3.04 3.03 3.02 2.31 2.29
1.59 1.58 1.57 1.28 1.27
2.00 3.00 3.00 2.00 3.00 3.00 6.00
8.0 7.5 7.0 6.5 6.0 5.5 5.0 4.5 4.0 3.5 3.0 2.5 2.0 1.5 1.0 0.5 0.0
f1 (ppm)

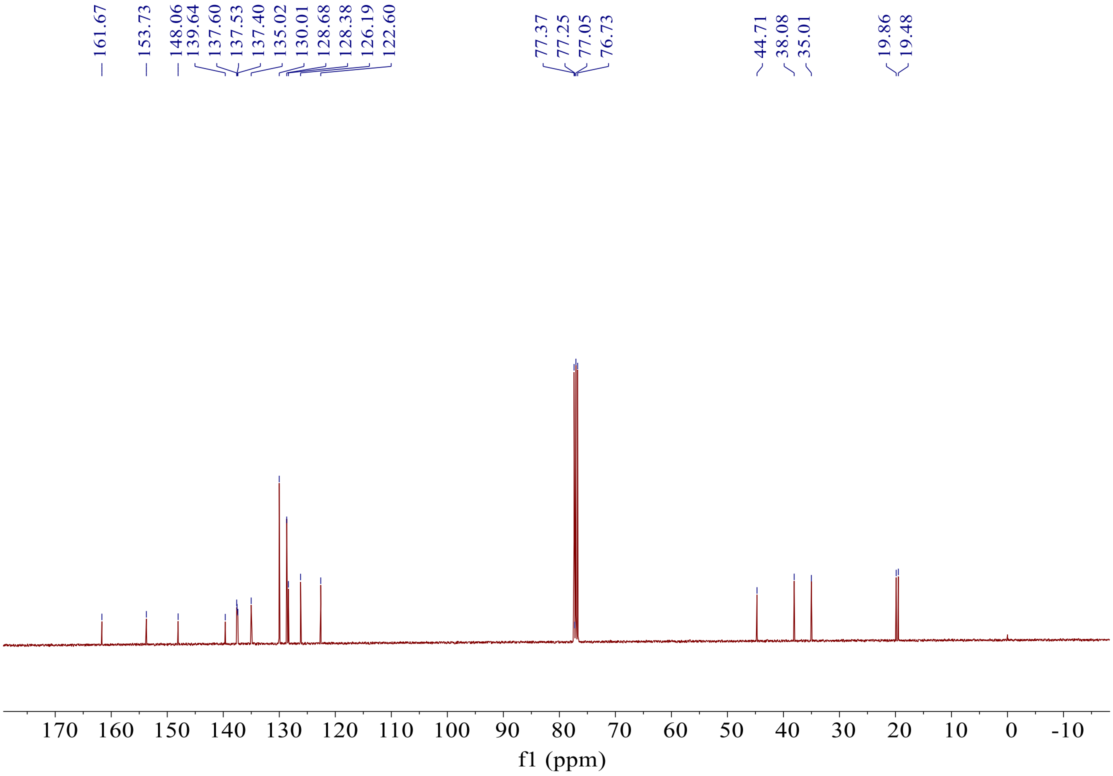
161.67
153.73
148.06 139.64 137.60 137.53 137.40 135.02 130.01 128.68 128.38 126.19 122.60
77.37 77.25 77.05 76.73
44.71 38.08 35.01
19.86 19.48
170 160 150 140 130 120 110 100 90 80 70 60 50 40 30 20 10 0 -10
f1 (ppm)

L6

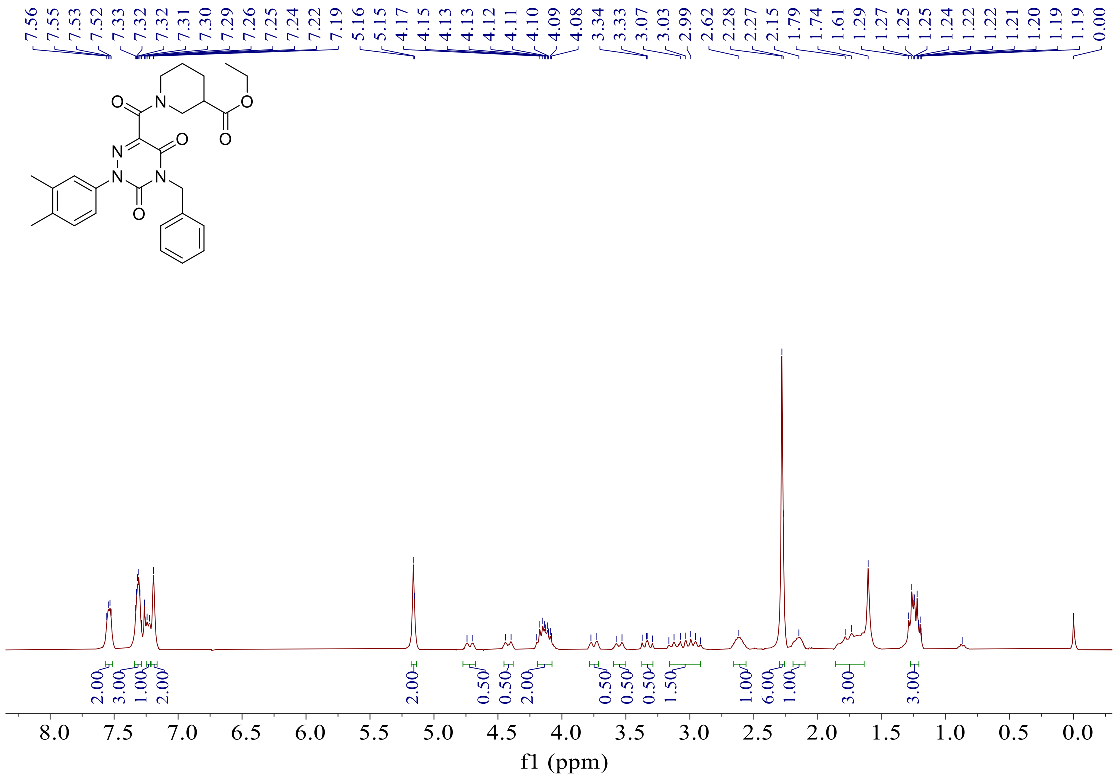
7.56 7.55 7.53 7.52 7.33 7.32 7.32 7.31 7.30 7.29 7.26 7.25 7.24 7.22 7.19 5.16 5.15 4.17 4.15 4.13 4.12 4.11 4.10 4.09 4.08 3.34 3.33 3.07 3.03 2.99 2.62 2.28 2.27 2.15 1.79 1.74 1.61 1.29 1.27 1.25 1.25 1.24 1.22 1.22 1.21 1.20 1.19 1.19 0.00
2.00 3.00 1.00 2.00 2.00 0.50 0.50 2.00 0.50 0.50 0.50 1.50 1.00 6.00 1.00 3.00 3.00
f1 (ppm)

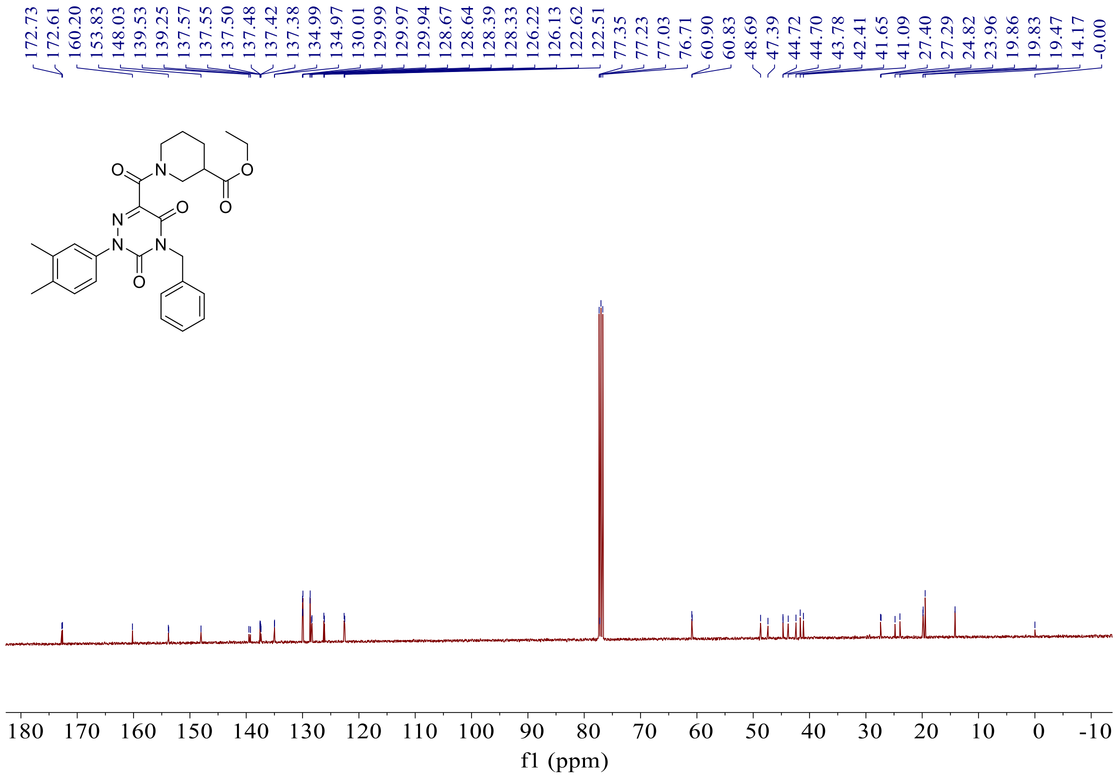
172.73 172.61 160.20 153.83 148.03 139.53 139.25 137.57 137.55 137.50 137.48 137.42 137.38 134.99 134.97 130.01 129.99 129.97 129.94 128.67 128.64 128.39 128.33 126.22 126.13 122.62 122.51 77.35 77.23 77.03 76.71 60.90 60.83 48.69 47.39 44.72 44.70 43.78 42.41 41.65 41.09 27.40 27.29 24.82 23.96 19.86 19.83 19.47 14.17 -0.00
f1 (ppm)

L7

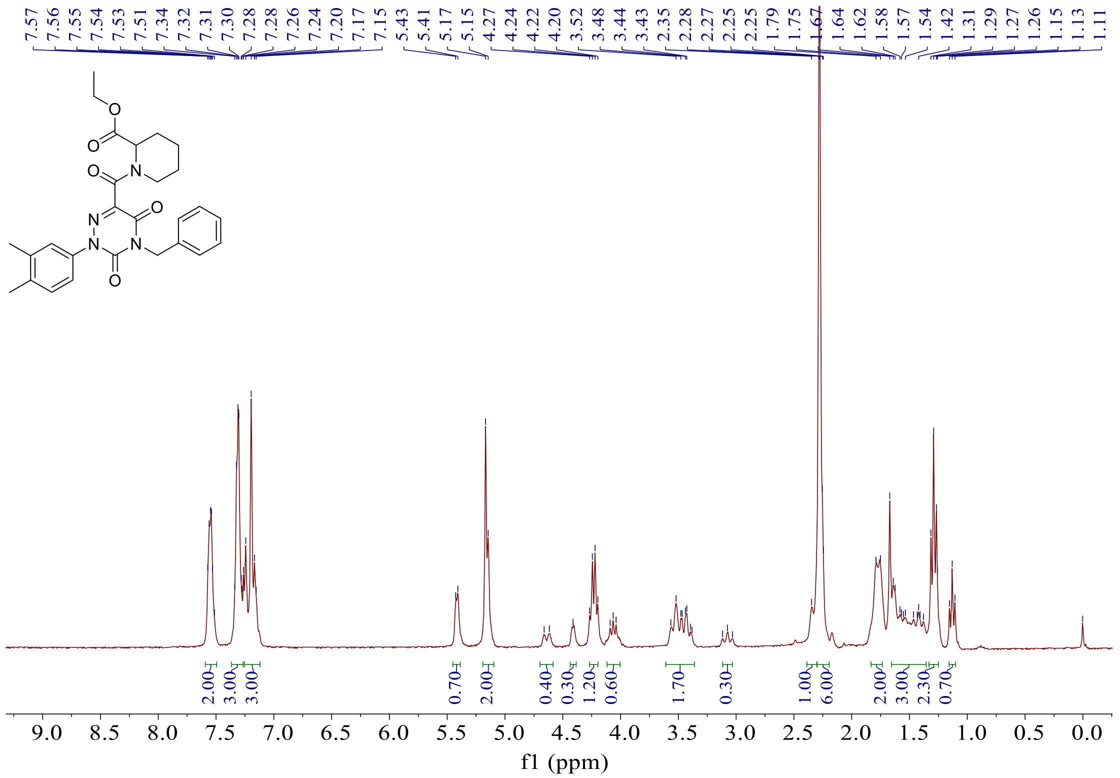
7.57 7.56 7.55 7.54 7.53 7.51 7.34 7.32 7.31 7.30 7.28 7.28 7.26 7.24 7.20 7.17 7.15 5.43 5.41 5.17 5.15 4.27 4.24 4.22 4.20 3.52 3.48 3.44 3.43 2.35 2.28 2.27 2.25 2.25 1.79 1.75 1.67 1.64 1.62 1.58 1.57 1.54 1.42 1.31 1.29 1.27 1.26 1.15 1.13 1.11
2.00 3.00 3.00 0.70 2.00 0.40 0.30 1.20 0.60 1.70 0.30 1.00 6.00 2.00 3.00 2.30 0.70
9.0 8.5 8.0 7.5 7.0 6.5 6.0 5.5 5.0 4.5 4.0 3.5 3.0 2.5 2.0 1.5 1.0 0.5 0.0
f1 (ppm)

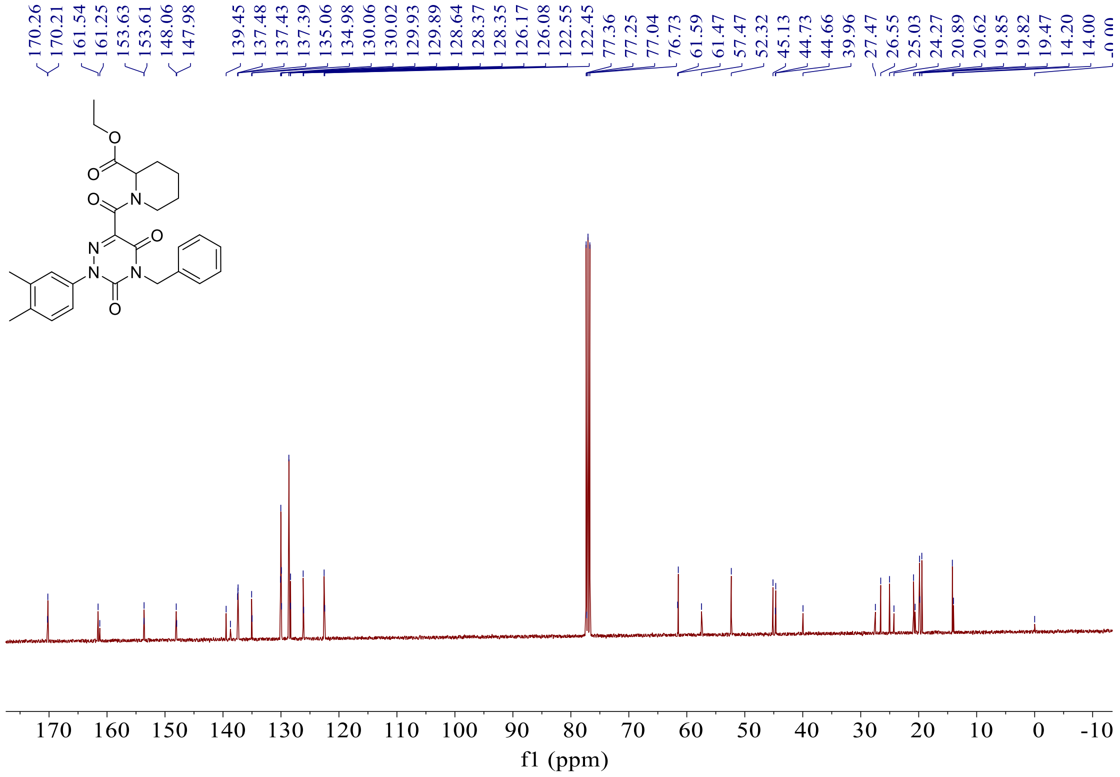
170.26 170.21 161.54 161.25 153.63 153.61 148.06 147.98 139.45 137.48 137.43 137.39 135.06 134.98 130.06 130.02 129.93 129.89 128.64 128.37 128.35 126.17 126.08 122.55 122.45 77.36 77.25 77.04 76.73 61.59 61.47 57.47 52.32 45.13 44.73 44.66 39.96 27.47 26.55 25.03 24.27 20.89 20.62 19.85 19.82 19.47 14.20 14.00
170 160 150 140 130 120 110 100 90 80 70 60 50 40 30 20 10 0 -10
f1 (ppm)

L8

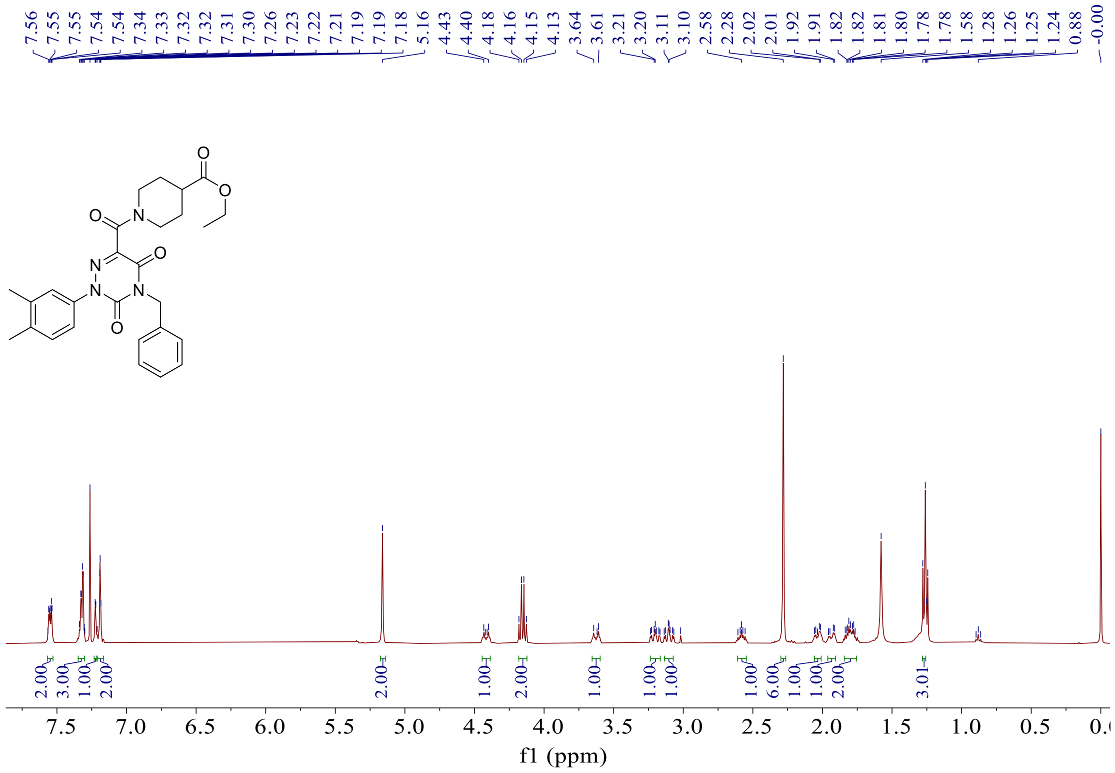
7.56 7.55 7.55 7.54 7.54 7.34 7.33 7.32 7.32 7.31 7.30 7.26 7.23 7.22 7.21 7.19 7.19 7.18 5.16 4.43 4.40 4.18 4.16 4.15 4.13 3.64 3.61 3.21 3.20 3.11 3.10 2.58 2.28 2.02 2.01 1.92 1.91 1.82 1.82 1.81 1.80 1.78 1.78 1.58 1.28 1.26 1.25 1.24 0.88 -0.00
2.00 3.00 1.00 2.00 2.00 1.00 2.00 1.00 1.00 1.00 1.00 6.00 1.00 1.00 2.00 3.01
7.5 7.0 6.5 6.0 5.5 5.0 4.5 4.0 3.5 3.0 2.5 2.0 1.5 1.0 0.5 0.0
f1 (ppm)

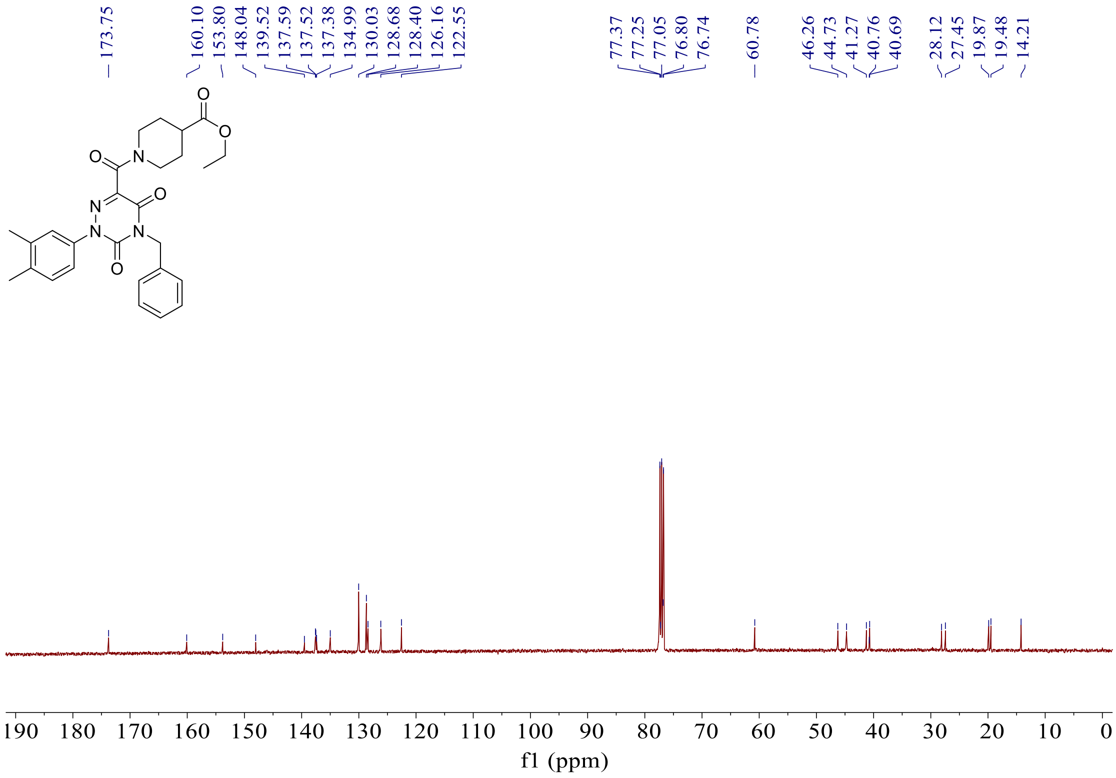
173.75 160.10 153.80 148.04 139.52 137.59 137.52 137.38 134.99 130.03 128.68 128.40 126.16 122.55 77.37 77.25 77.05 76.80 76.74 60.78 46.26 44.73 41.27 40.76 40.69 28.12 27.45 19.87 19.48 14.21
190 180 170 160 150 140 130 120 110 100 90 80 70 60 50 40 30 20 10 0
f1 (ppm)

L9

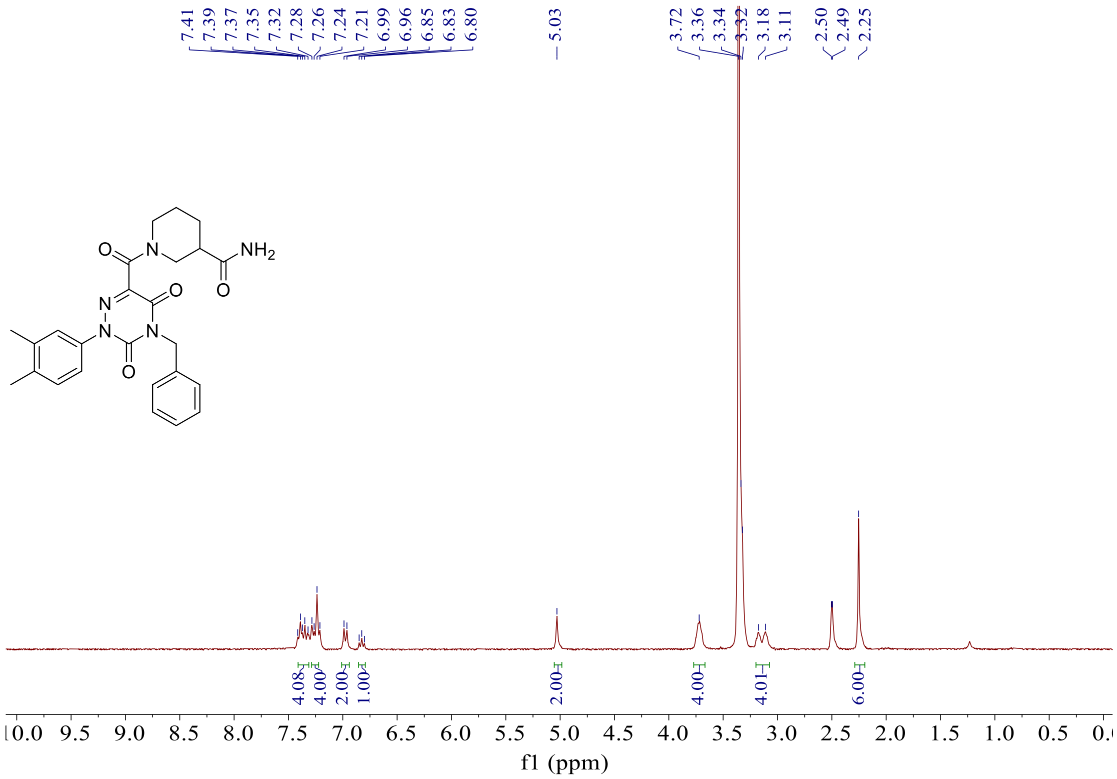
7.41 7.39 7.37 7.35 7.32 7.28 7.26 7.24 7.21 6.99 6.96 6.85 6.83 6.80
5.03
3.72 3.36 3.34 3.32 3.18 3.11
2.50 2.49 2.25
O
N
NH2
N
O
O
N
N
N
O
4.08 4.00 2.00 1.00
2.00
4.00
4.01
6.00
10.0 9.5 9.0 8.5 8.0 7.5 7.0 6.5 6.0 5.5 5.0 4.5 4.0 3.5 3.0 2.5 2.0 1.5 1.0 0.5 0.0
f1 (ppm)

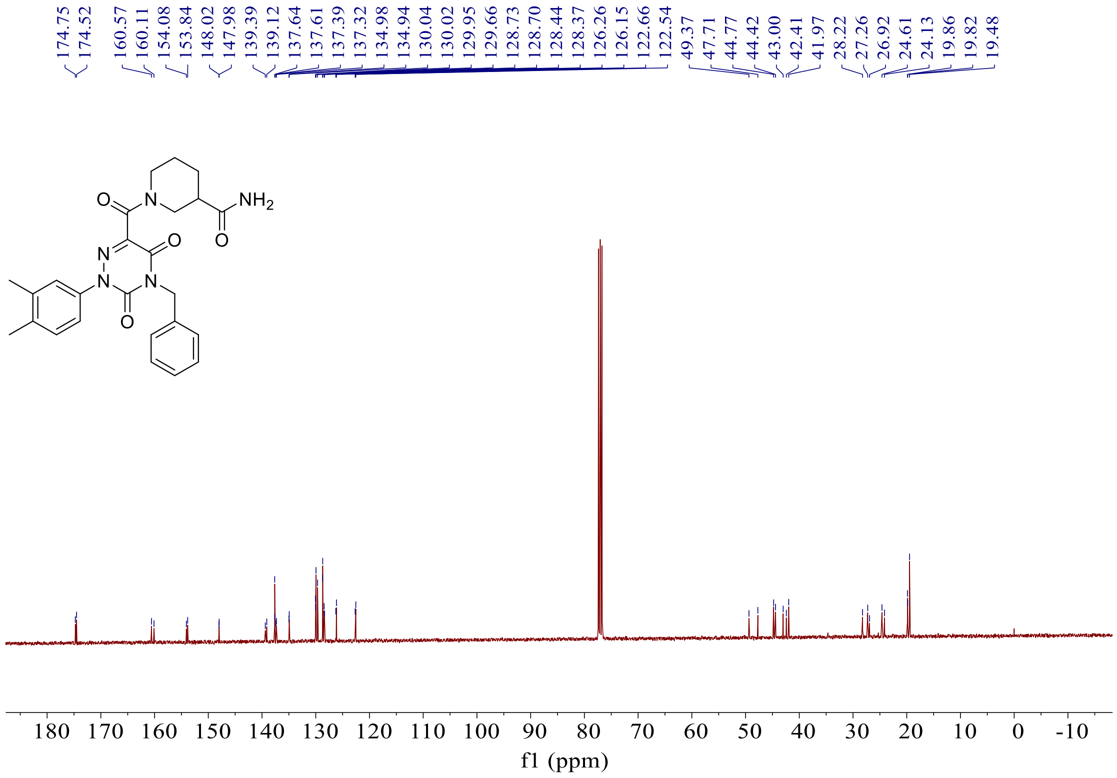
174.75 174.52
160.57 160.11 154.08 153.84 148.02 147.98 139.39 139.12 137.64 137.61 137.39 137.32 134.98 134.94 130.04 130.02 129.95 129.66 128.73 128.70 128.44 128.37 126.26 126.15 122.66 122.54
49.37 47.71 44.77 44.42 43.00 42.41 41.97 28.22 27.26 26.92 24.61 24.13 19.86 19.82 19.48
O
N
NH2
N
O
O
N
N
N
O
180 170 160 150 140 130 120 110 100 90 80 70 60 50 40 30 20 10 0 -10
f1 (ppm)

L10

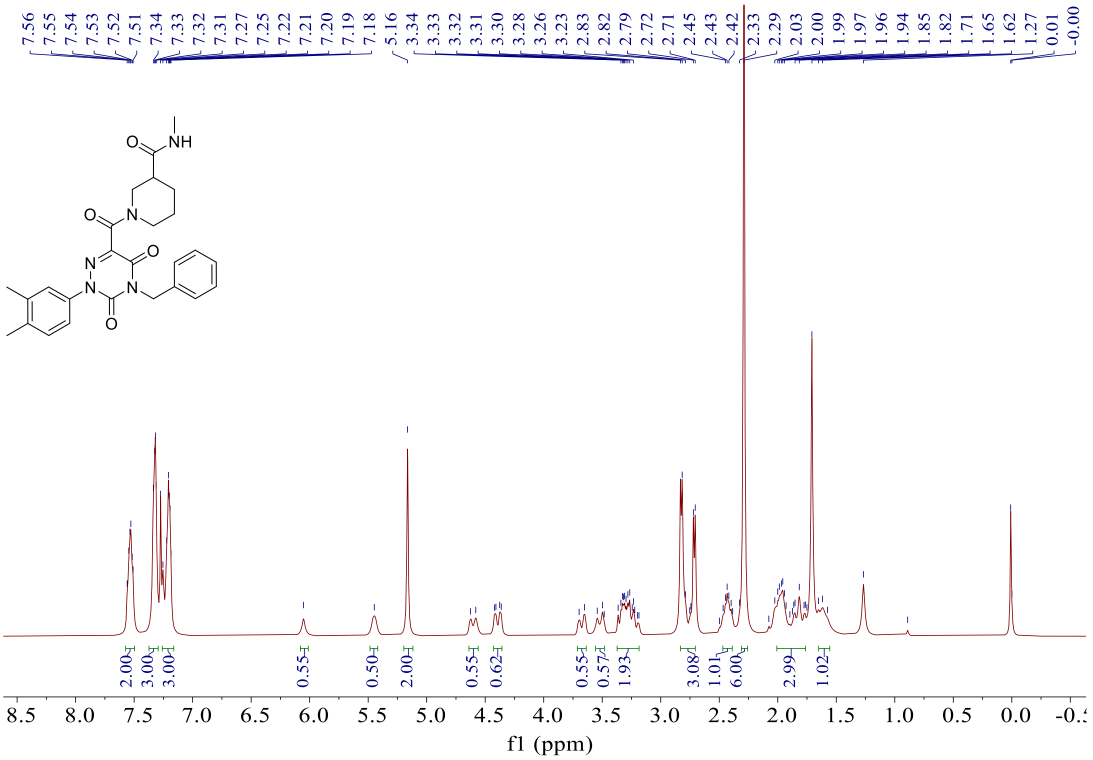

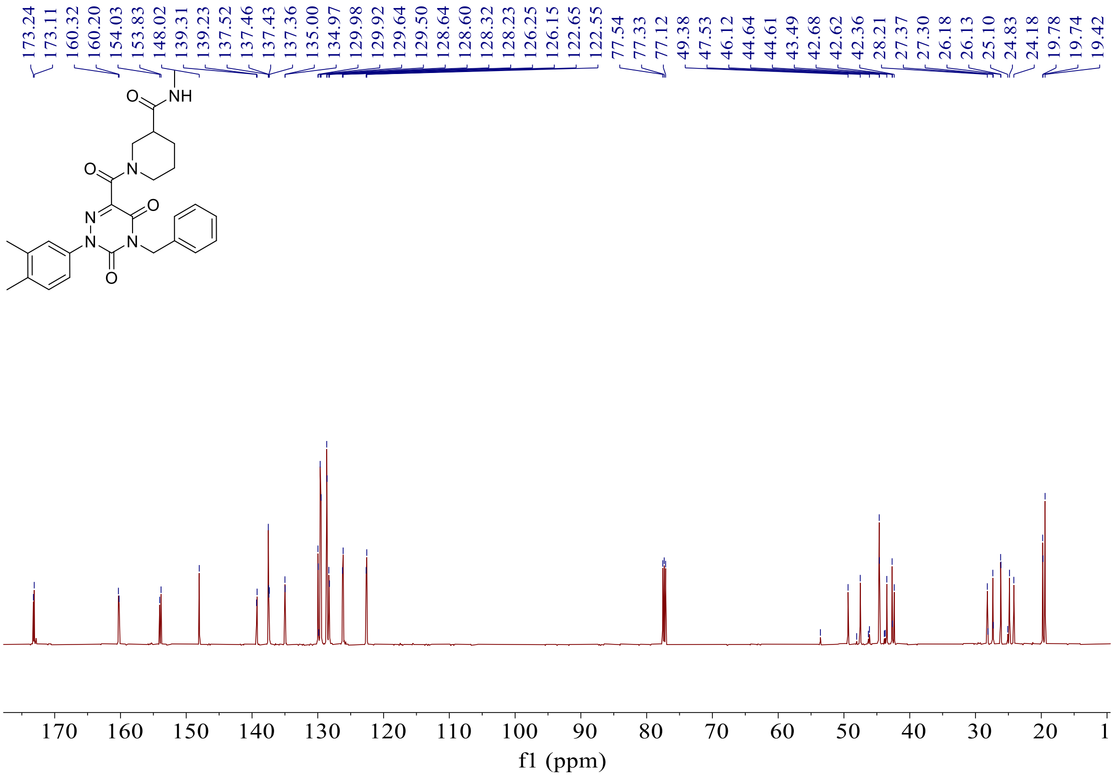

L11

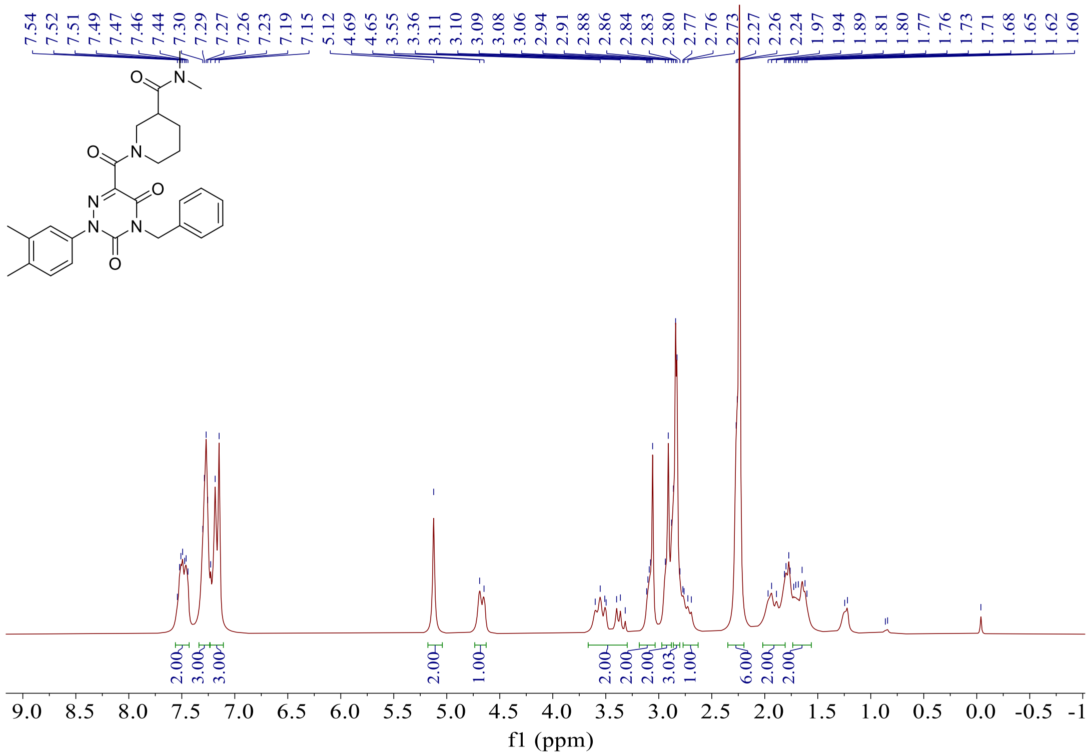
7.54
7.52
7.51
7.49
7.47
7.46
7.44
7.30
7.29
7.27
7.26
7.23
7.19
7.15
5.12
4.69
4.65
3.55
3.36
3.11
3.10
3.09
3.08
3.06
2.94
2.91
2.88
2.86
2.84
2.83
2.80
2.77
2.76
2.73
2.27
2.26
2.24
1.97
1.94
1.89
1.81
1.80
1.77
1.76
1.73
1.71
1.68
1.65
1.62
1.60
2.00
3.00
3.00
2.00
1.00
2.00
2.00
2.00
3.03
1.00
6.00
2.00
2.00
9.0 8.5 8.0 7.5 7.0 6.5 6.0 5.5 5.0 4.5 4.0 3.5 3.0 2.5 2.0 1.5 1.0 0.5 0.0 -0.5 -1
f1 (ppm)

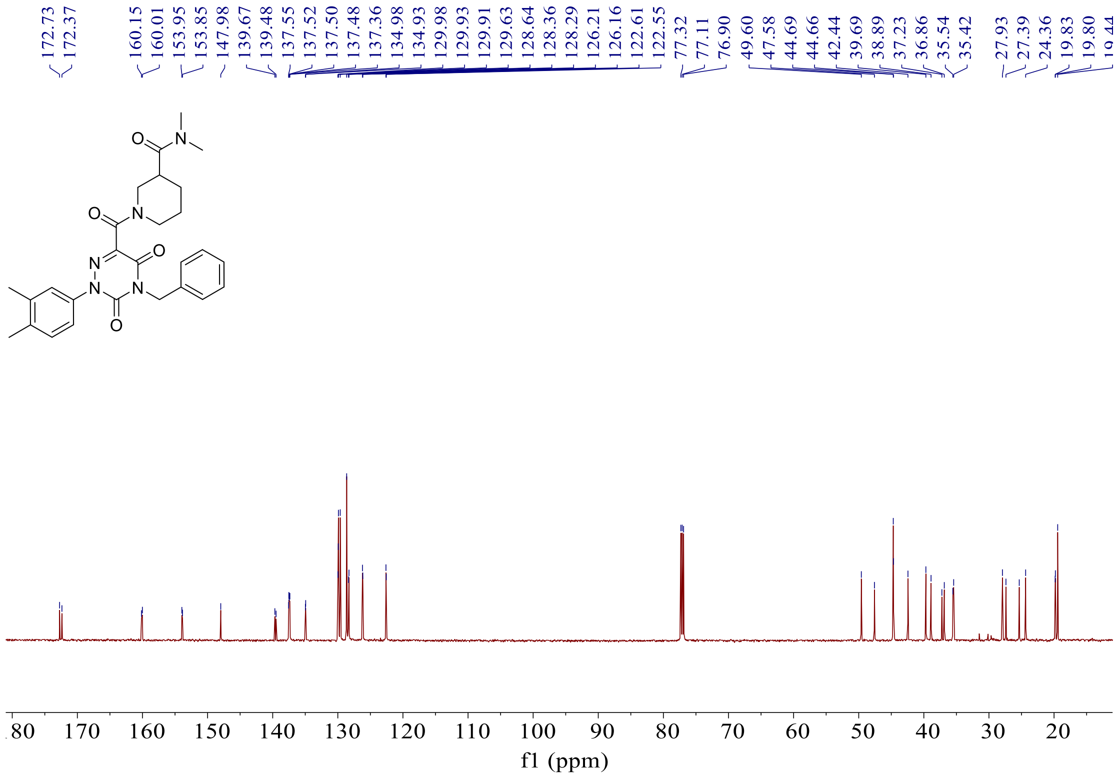
172.73
172.37
160.15
160.01
153.95
153.85
147.98
139.67
139.48
137.55
137.52
137.50
137.48
137.36
134.98
134.93
129.98
129.93
129.91
129.63
128.64
128.36
128.29
126.21
126.16
122.61
122.55
77.32
77.11
76.90
49.60
47.58
44.69
44.66
42.44
39.69
38.89
37.23
36.86
35.54
35.42
27.93
27.39
24.36
19.83
19.80
80 170 160 150 140 130 120 110 100 90 80 70 60 50 40 30 20
f1 (ppm)

L12

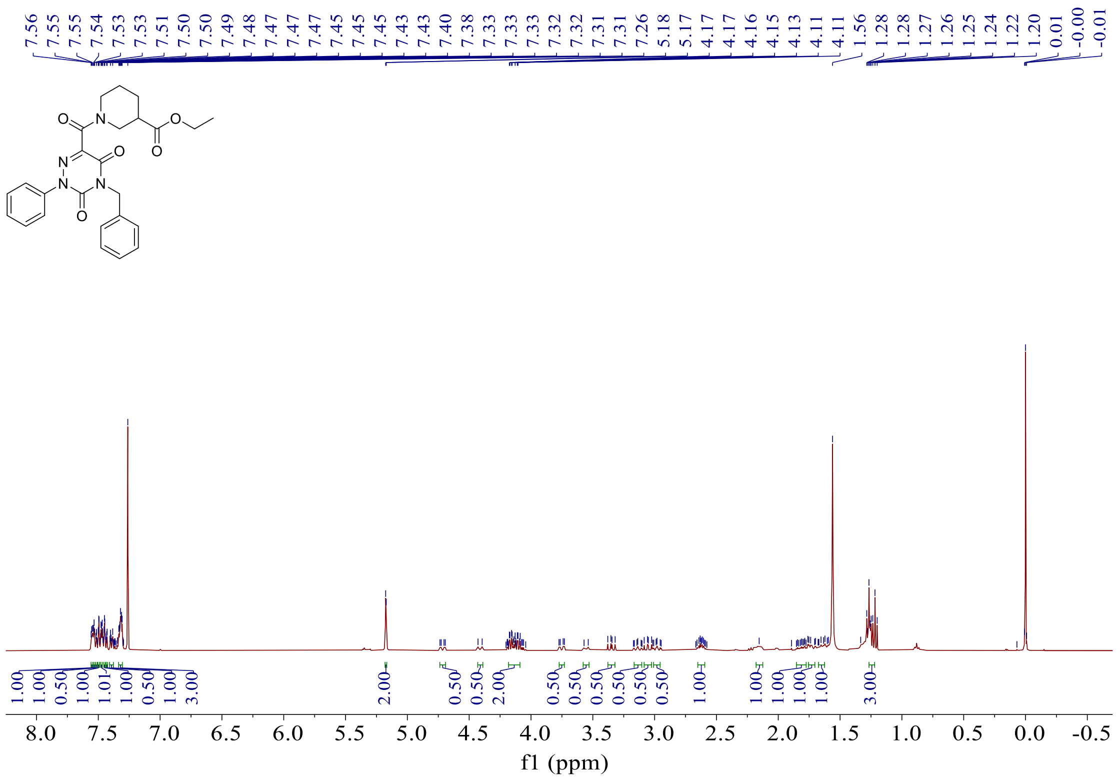

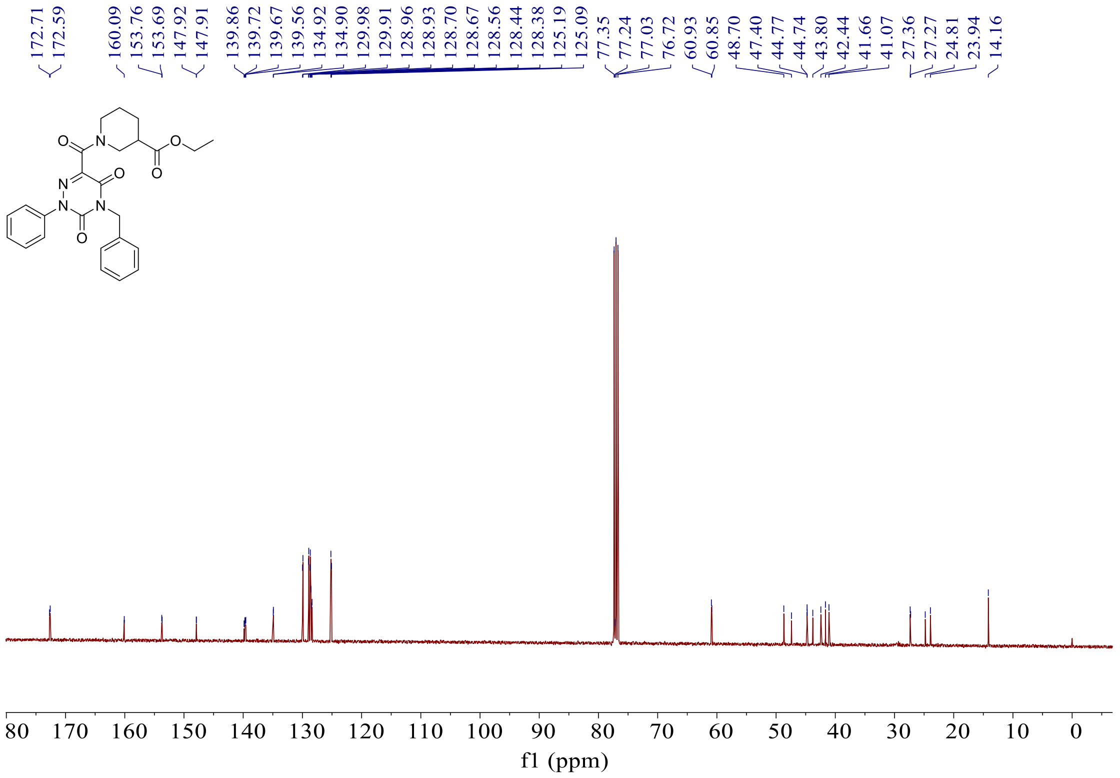

L13

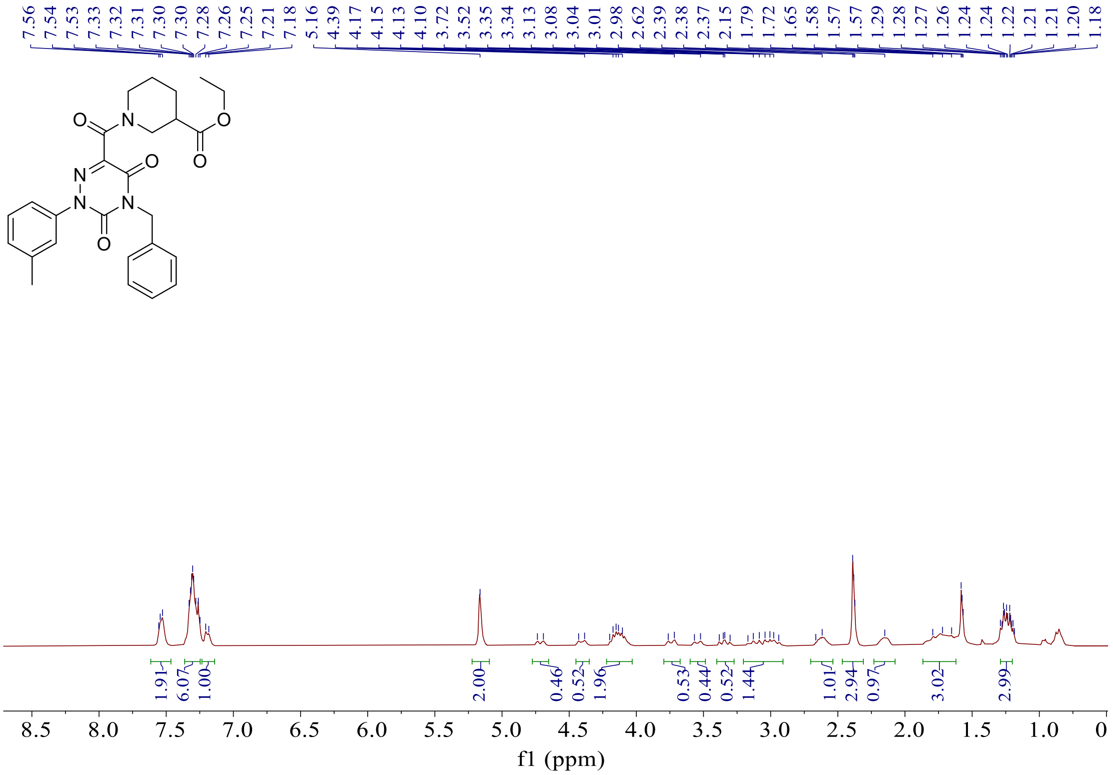
7.56 7.54 7.53 7.33 7.32 7.31 7.30 7.30 7.28 7.26 7.25 7.21 7.18 5.16 4.39 4.17 4.15 4.13 4.10 3.72 3.52 3.35 3.34 3.13 3.08 3.04 3.01 2.98 2.62 2.39 2.38 2.37 2.15 1.79 1.72 1.65 1.58 1.57 1.57 1.29 1.28 1.27 1.26 1.24 1.24 1.22 1.21 1.21 1.20 1.18
1.91 6.07 1.00 2.00 0.46 0.52 1.96 0.53 0.44 0.52 1.44 1.01 2.94 0.97 3.02 2.99
8.5 8.0 7.5 7.0 6.5 6.0 5.5 5.0 4.5 4.0 3.5 3.0 2.5 2.0 1.5 1.0 0
f1 (ppm)

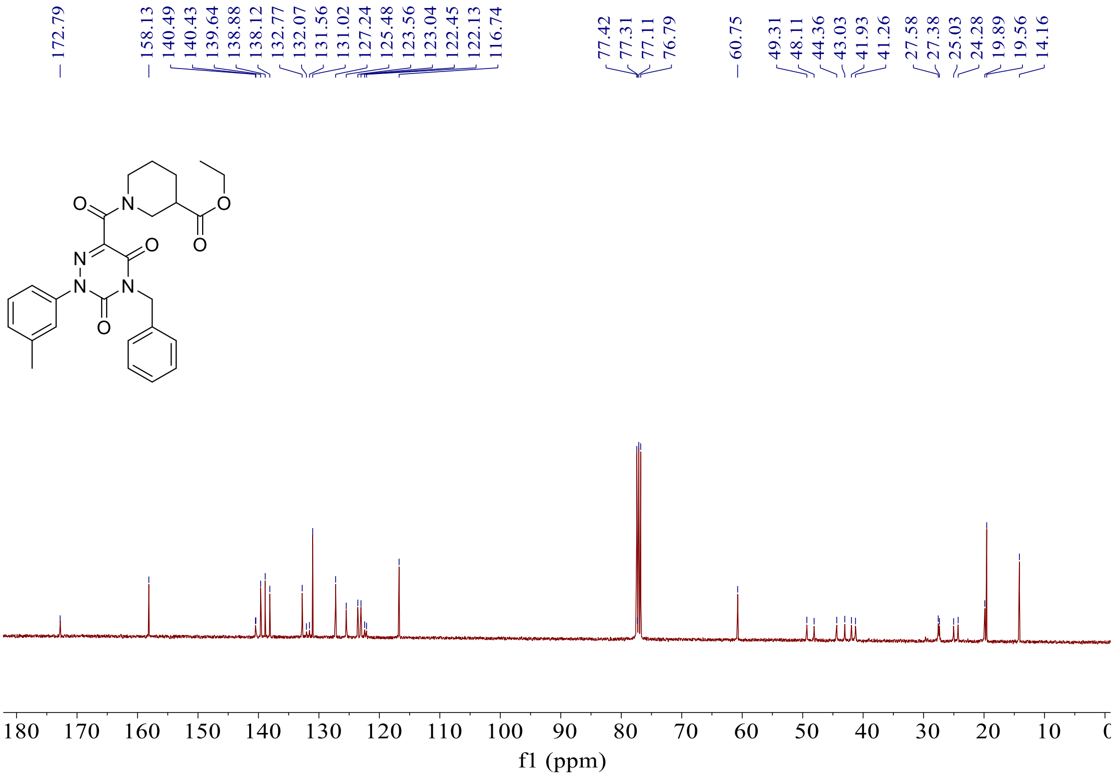
172.79 158.13 140.49 140.43 139.64 138.88 138.12 132.77 132.07 131.56 131.02 127.24 125.48 123.56 123.04 122.45 122.13 116.74 77.42 77.31 77.11 76.79 60.75 49.31 48.11 44.36 43.03 41.93 41.26 27.58 27.38 25.03 24.28 19.89 19.56 14.16
180 170 160 150 140 130 120 110 100 90 80 70 60 50 40 30 20 10 0
f1 (ppm)

L14

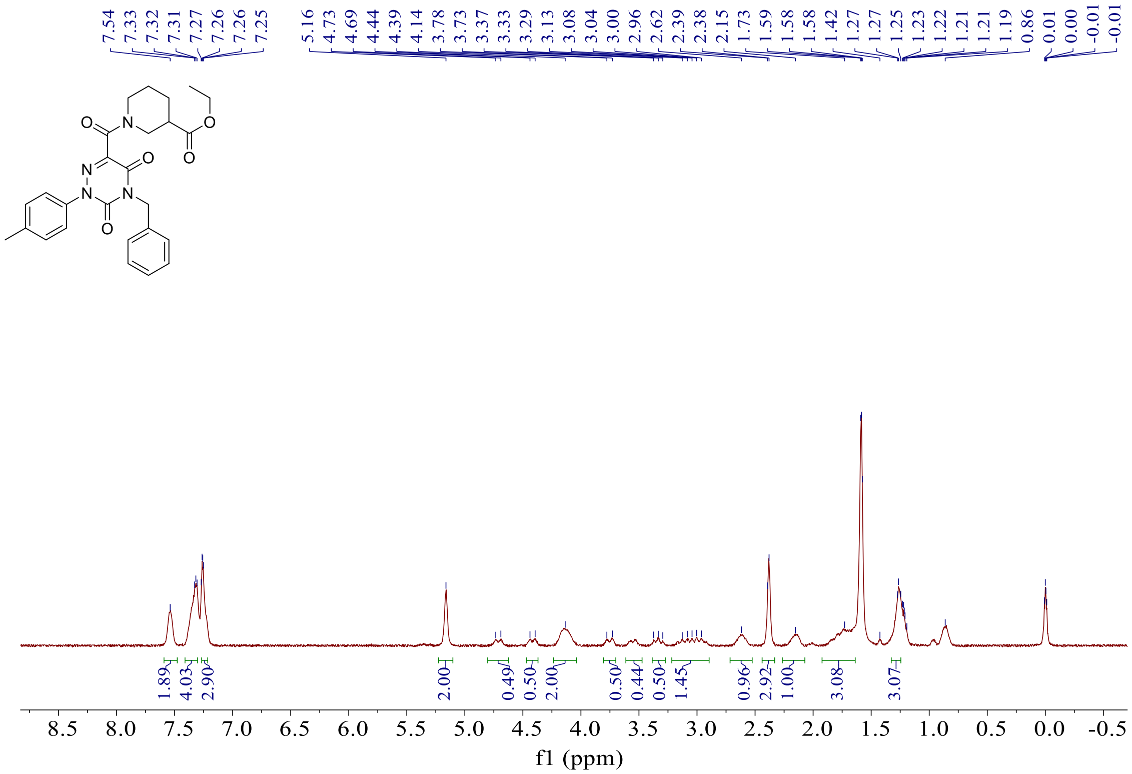
7.54 7.33 7.32 7.31 7.27 7.26 7.26 7.25 5.16 4.73 4.69 4.44 4.39 4.14 3.78 3.73 3.37 3.33 3.29 3.13 3.08 3.04 3.00 2.96 2.62 2.39 2.38 2.15 1.73 1.59 1.58 1.42 1.27 1.27 1.25 1.23 1.22 1.21 1.21 1.19 0.86 0.01 0.00 -0.01 -0.01
O
N
O
N
N
O
N
O
O
O
1.89 4.03 2.90 2.00 0.49 0.50 2.00 0.50 0.44 0.50 1.45 0.96 2.92 1.00 3.08 3.07
8.5 8.0 7.5 7.0 6.5 6.0 5.5 5.0 4.5 4.0 3.5 3.0 2.5 2.0 1.5 1.0 0.5 0.0 -0.5
f1 (ppm)

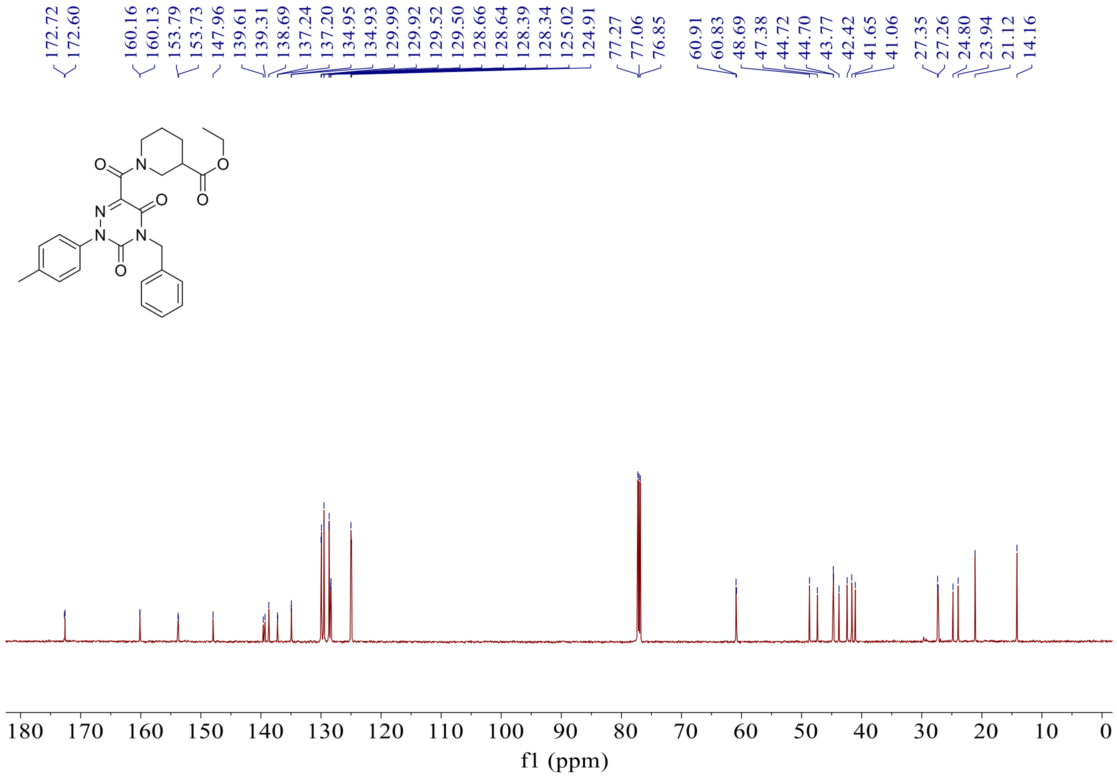
172.72 172.60 160.16 160.13 153.79 153.73 147.96 139.61 139.31 138.69 137.24 137.20 134.95 134.93 129.99 129.92 129.52 129.50 128.66 128.64 128.39 128.34 125.02 124.91 77.27 77.06 76.85 60.91 60.83 48.69 47.38 44.72 44.70 43.77 42.42 41.65 41.06 27.35 27.26 24.80 23.94 21.12 14.16
O
N
O
N
N
O
N
O
O
O
180 170 160 150 140 130 120 110 100 90 80 70 60 50 40 30 20 10 0
f1 (ppm)

L15

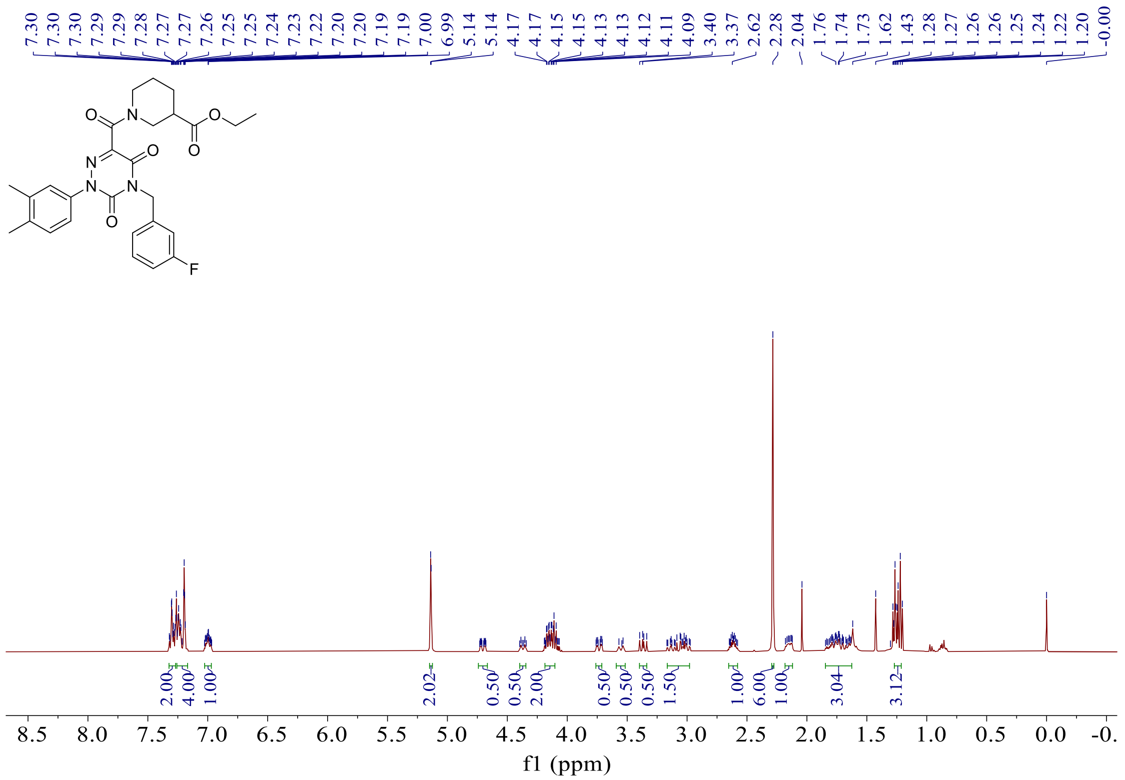

7.30 7.30 7.30 7.29 7.29 7.28 7.27 7.27 7.26 7.25 7.25 7.24 7.23 7.22 7.20 7.20 7.19 7.19 7.00 6.99 5.14 5.14 4.17 4.17 4.15 4.15 4.13 4.13 4.12 4.11 4.09 3.40 3.37 2.62 2.28 2.04 1.76 1.74 1.73 1.62 1.43 1.28 1.27 1.26 1.26 1.25 1.24 1.22 1.20 -0.00
2.00 4.00 1.00 2.02 0.50 0.50 2.00 0.50 0.50 0.50 1.50 1.00 6.00 1.00 3.04 3.12
f1 (ppm)

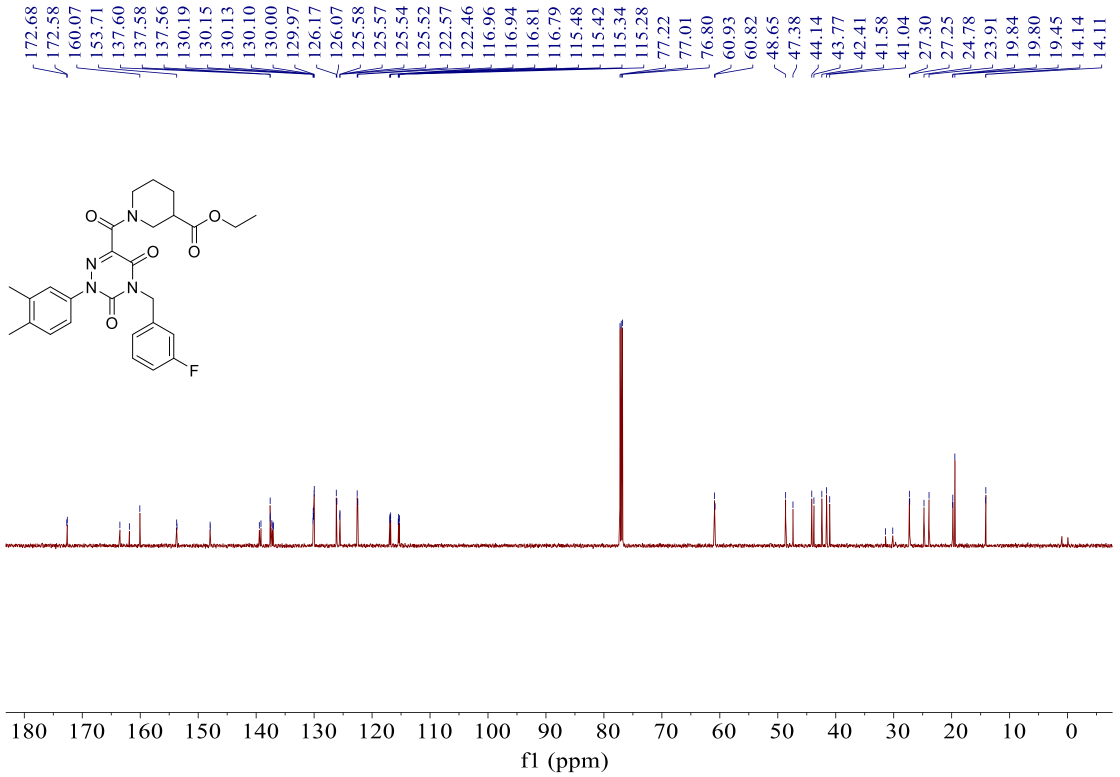

172.68 172.58 160.07 153.71 137.60 137.58 137.56 130.19 130.15 130.13 130.10 130.00 129.97 126.17 126.07 125.58 125.57 125.54 125.52 122.57 122.46 116.96 116.94 116.81 116.79 115.48 115.42 115.34 115.28 77.22 77.01 76.80 60.93 60.82 48.65 47.38 44.14 43.77 42.41 41.58 41.04 27.30 27.25 24.78 23.91 19.84 19.80 19.45 14.14 14.11
f1 (ppm)

L16

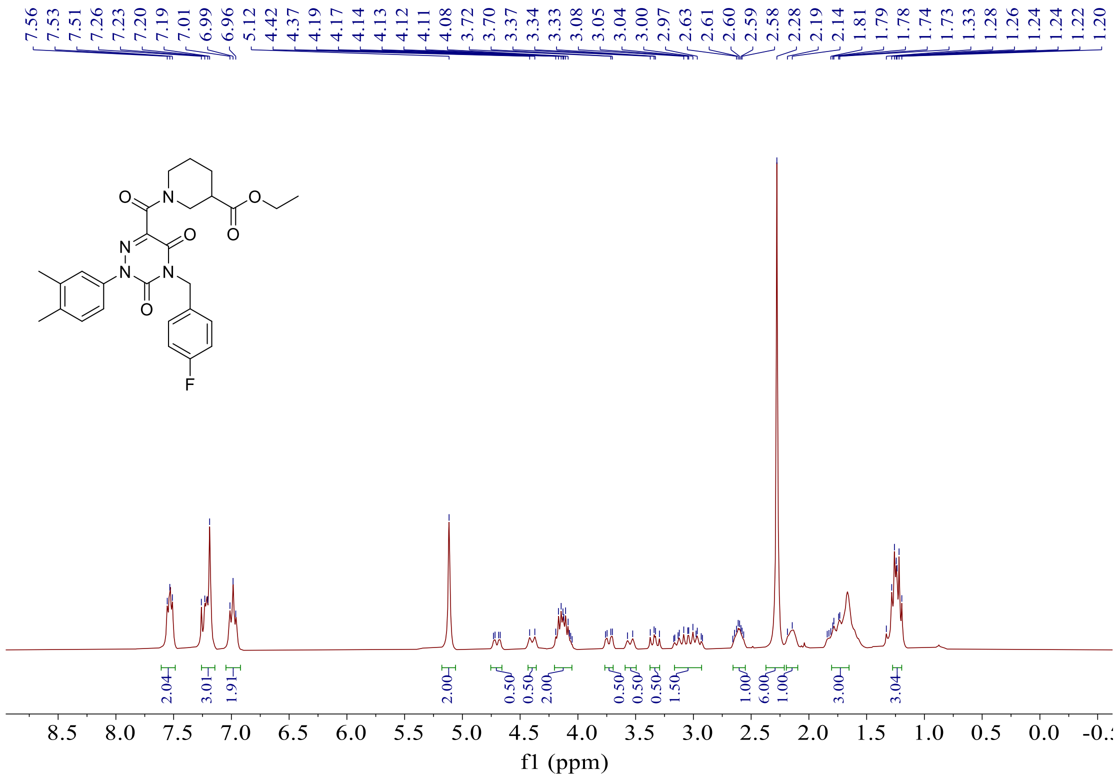

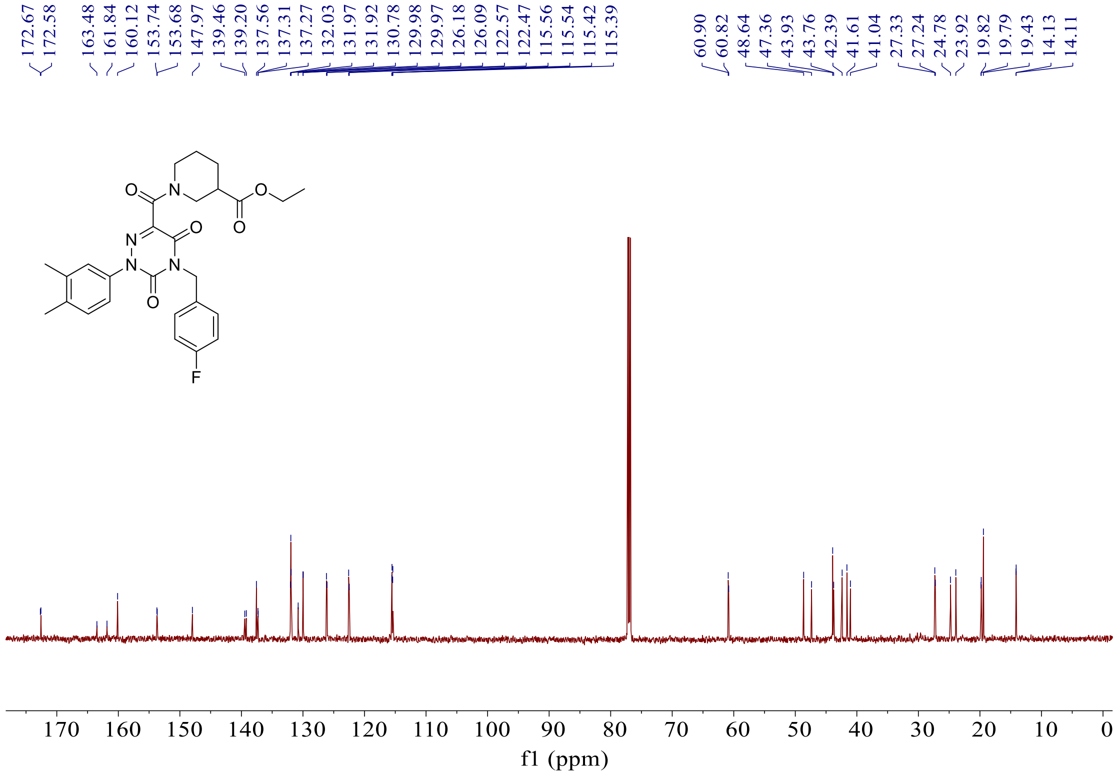

HR-MS Spectra

L5

Elemental Composition Report **Page 1**

Multiple Mass Analysis: 2 mass(es) processed - displaying only valid results
Tolerance = 5.0 PPM / DBE: min = -1.5, max = 50.0
Element prediction: Off

Monoisotopic Mass, Odd and Even Electron Ions
290 formula(e) evaluated with 1 results within limits (all results (up to 1000) for each mass)
Elements Used:
C: 0-27 H: 0-22 N: 0-4 16O: 0-3 35Cl: 0-1 37Cl: 0-1
DEFAULT
ZRF-3-436 239 (3.983) Cm (239:244-(97:113+63:86)) TOF MS EI+ 2.39e+004

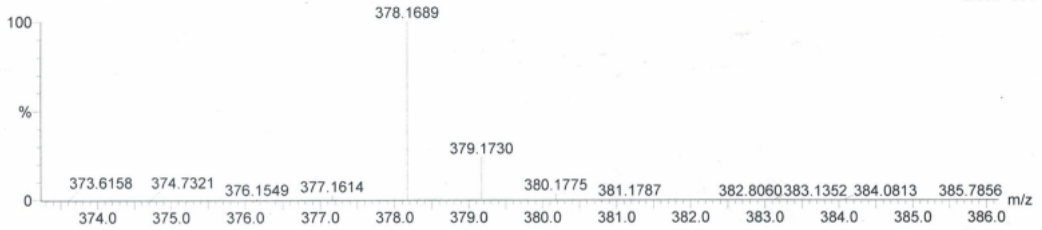

Minimum:	10.00				-1.5		
Maximum:	100.00		30.0	5.0	50.0		
Mass	RA	Calc. Mass	mDa	PPM	DBE	i-FIT	Formula
378.1689	100.00	378.1692	-0.3	-0.8	13.0	7.3	C21 H22 N4 16O3

L6

Elemental Composition Report **Page 1**

Multiple Mass Analysis: 2 mass(es) processed - displaying only valid results
Tolerance = 5.0 PPM / DBE: min = -1.5, max = 50.0
Element prediction: Off

Monoisotopic Mass, Odd and Even Electron Ions
223 formula(e) evaluated with 1 results within limits (all results (up to 1000) for each mass)
Elements Used:
C: 0-27 H: 0-30 N: 0-4 16O: 0-5 35Cl: 0-1 37Cl: 0-1
DEFAULT
ZRF-3-579 308 (5.133) Cm (308:318) TOF MS EI+ 3.91e+003

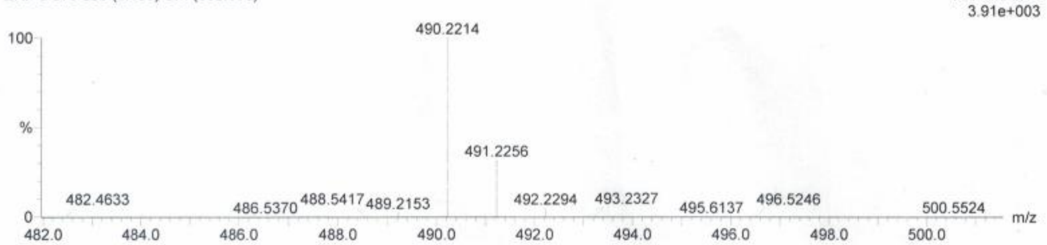

Minimum:	10.00				-1.5		
Maximum:	100.00		30.0	5.0	50.0		
Mass	RA	Calc. Mass	mDa	PPM	DBE	i-FIT	Formula
490.2214	100.00	490.2216	-0.2	-0.4	15.0	6.3	C27 H30 N4 16O5

L7

Elemental Composition Report **Page 1**

Single Mass Analysis
Tolerance = 5.0 PPM / DBE: min = -1.5, max = 50.0
Element prediction: Off
Number of isotope peaks used for i-FIT = 3

Monoisotopic Mass, Even Electron Ions
2668 formula(e) evaluated with 1 results within limits (up to 50 best isotopic matches for each mass)
Elements Used:
C: 0-27 H: 18-31 B: 0-1 N: 0-5 O: 0-5 Na: 0-1 35Cl: 0-8 K: 0-1
3-580 28 (0.536)
1: TOF MS ES+

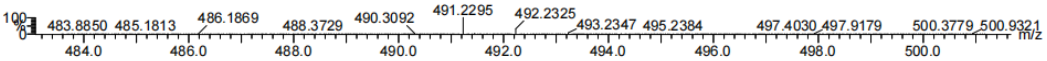

Minimum:				-1.5				
Maximum:		5.0	5.0	50.0				
Mass	Calc. Mass	mDa	PPM	DBE	i-FIT	Norm	Conf(%)	Formula
491.2295	491.2294	0.1	0.2	14.5	815.3	n/a	n/a	C27 H31 N4 O5

L8

Elemental Composition Report **Page 1**

Single Mass Analysis
Tolerance = 5.0 PPM / DBE: min = -1.5, max = 50.0
Element prediction: Off
Number of isotope peaks used for i-FIT = 3

Monoisotopic Mass, Even Electron Ions
2668 formula(e) evaluated with 1 results within limits (up to 50 best isotopic matches for each mass)
Elements Used:
C: 0-27 H: 18-31 B: 0-1 N: 0-5 O: 0-5 Na: 0-1 35Cl: 0-8 K: 0-1
3-583 4 (0.096)
1: TOF MS ES+

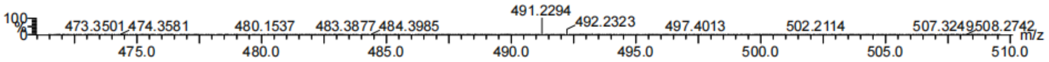

Minimum:				-1.5				
Maximum:		5.0	5.0	50.0				
Mass	Calc. Mass	mDa	PPM	DBE	i-FIT	Norm	Conf(%)	Formula
491.2294	491.2294	0.0	0.0	14.5	772.9	n/a	n/a	C27 H31 N4 O5

L14

Elemental Composition Report **Page 1**

Tolerance = 2.0 PPM / DBE: min = -1.5, max = 50.0
Element prediction: Off

Monoisotopic Mass, Odd and Even Electron Ions
173 formula(e) evaluated with 1 results within limits (all results (up to 1000) for each mass)
Elements Used:
C: 0-26 H: 0-28 N: 0-5 16O: 0-6 F: 0-3
DEFAULT
2-723 362 (6.033) Cm (350:362-(127:155+73:94))

TOF MS EI+
3.75e+003

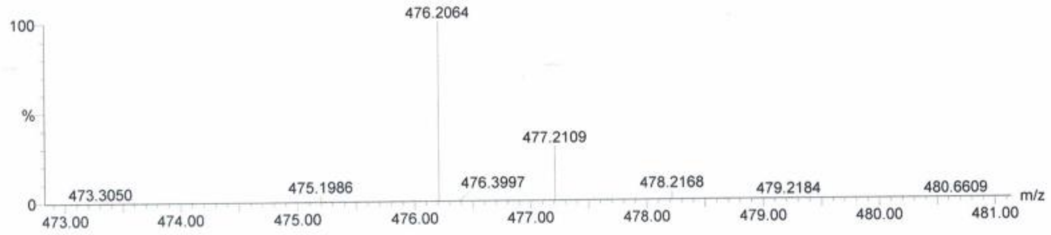

Minimum:	50.00				-1.5		
Maximum:	100.00		30.0	2.0	50.0		
Mass	RA	Calc. Mass	mDa	PPM	DBE	i-FIT	Formula
476.2064	100.00	476.2060	0.4	0.8	15.0	9.8	C26 H28 N4 16O5

L15

Elemental Composition Report **Page 1**

Multiple Mass Analysis: 2 mass(es) processed - displaying only valid results
Tolerance = 5.0 PPM / DBE: min = -1.5, max = 50.0
Element prediction: Off

Monoisotopic Mass, Odd and Even Electron Ions
429 formula(e) evaluated with 1 results within limits (all results (up to 1000) for each mass)
Elements Used:
C: 0-27 H: 0-29 N: 0-4 16O: 0-5 F: 0-1 35Cl: 0-1 37Cl: 0-1
DEFAULT
ZRF-2-336 593 (9.883) Cm (585:593-(498:507+476:490))

TOF MS EI+
1.14e+004

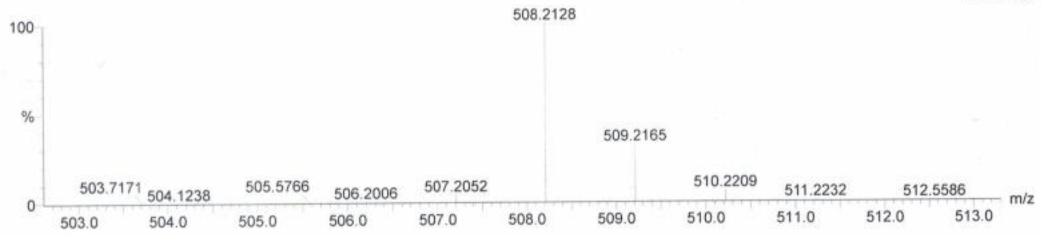

Minimum:	10.00				-1.5		
Maximum:	100.00		30.0	5.0	50.0		
Mass	RA	Calc. Mass	mDa	PPM	DBE	i-FIT	Formula
508.2128	100.00	508.2122	0.6	1.2	15.0	0.6	C27 H29 N4 16O5 F

HPLC Spectra

L5

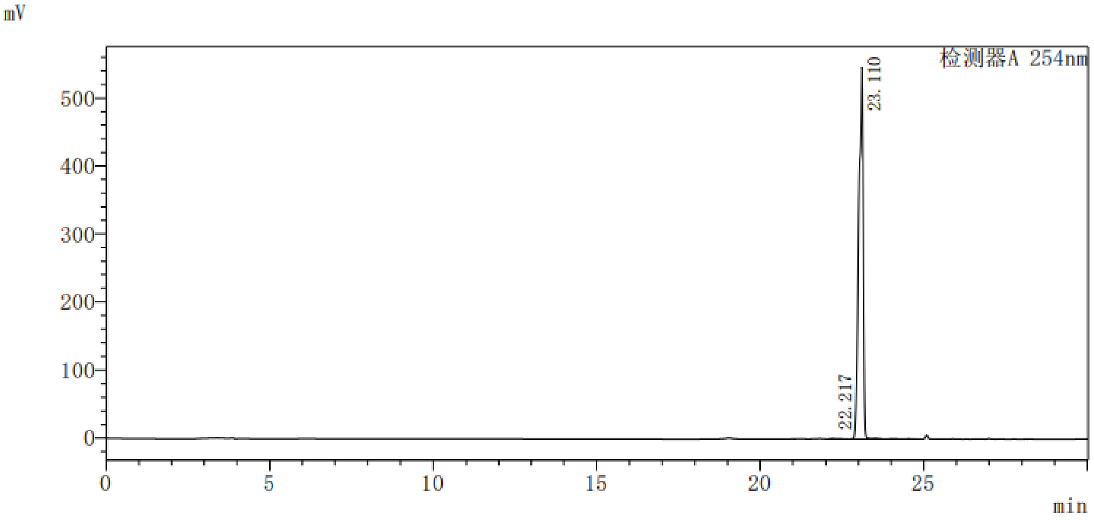

Detector A 254 nm

Peaks Time Area% Area Height

峰号	保留时间	面积%	面积	高度
1	22.217	0.150	8032	598
2	23.110	99.850	5332524	546577
总计		100.000	5340556	547175

L6

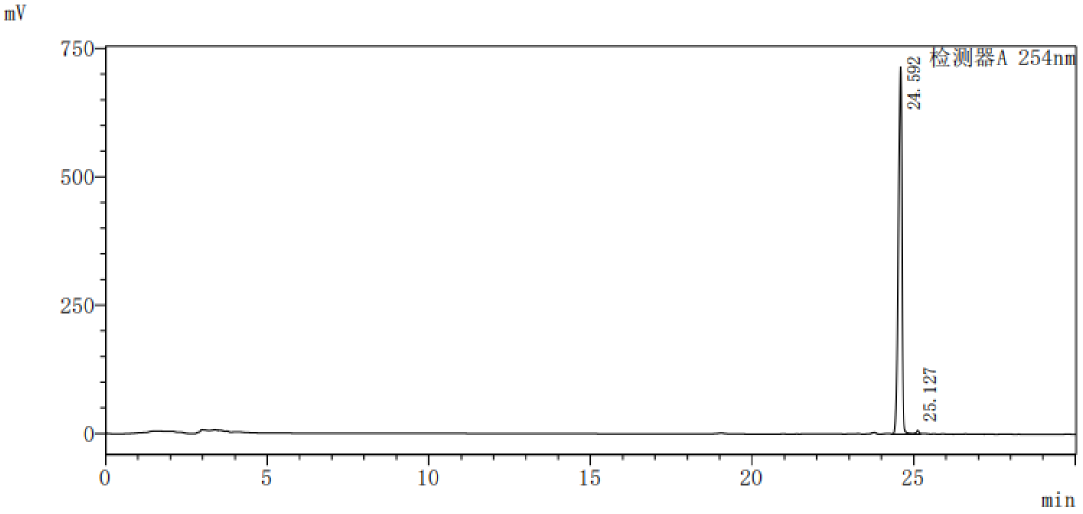

Detector A 254 nm

Peaks Time Area% Area Height

峰号	保留时间	面积%	面积	高度
1	24.592	99.143	5400749	715446
2	25.127	0.857	46700	6824
总计		100.000	5447448	722271

L7

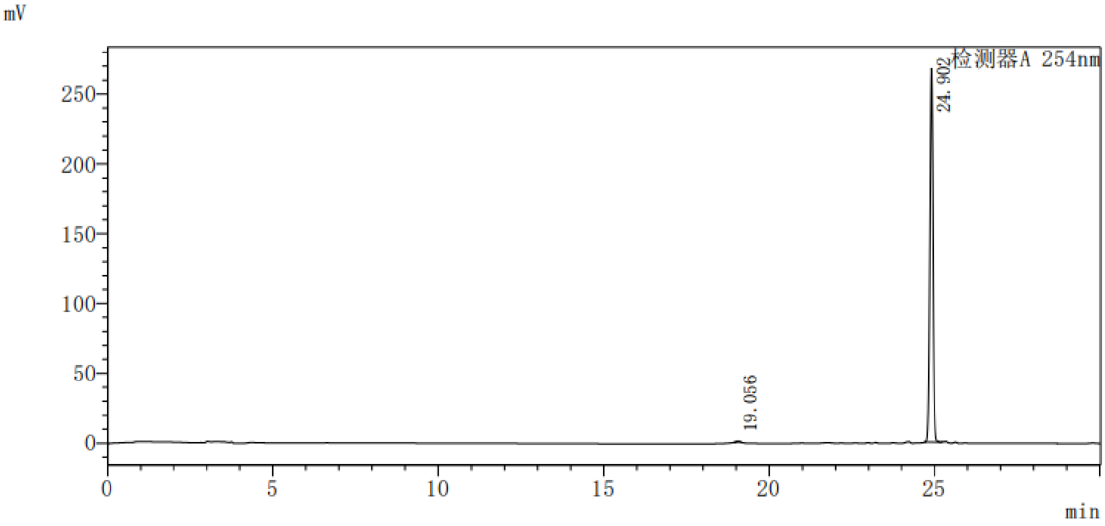

Detector A 254 nm

Peaks Time Area% Area Height

峰号	保留时间	面积%	面积	高度
1	19.056	0.222	3937	643
2	24.902	99.778	1769507	267666
总计		100.000	1773444	268309

L8

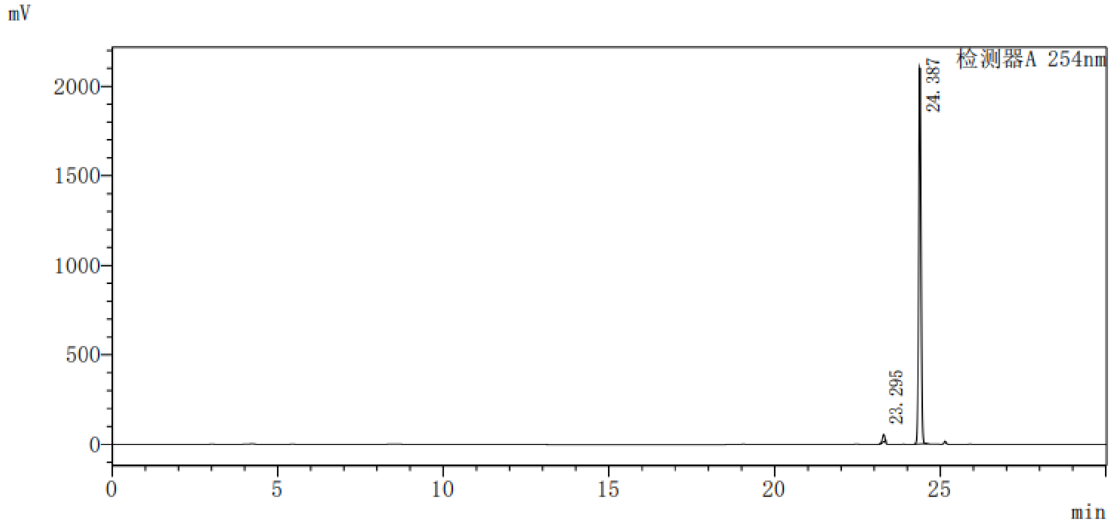

Detector A 254 nm

Peaks Time Area% Area Height

峰号	保留时间	面积%	面积	高度
1	23.295	1.816	197683	40932
2	24.387	98.184	10686956	2098368
总计		100.000	10884639	2139301

L14

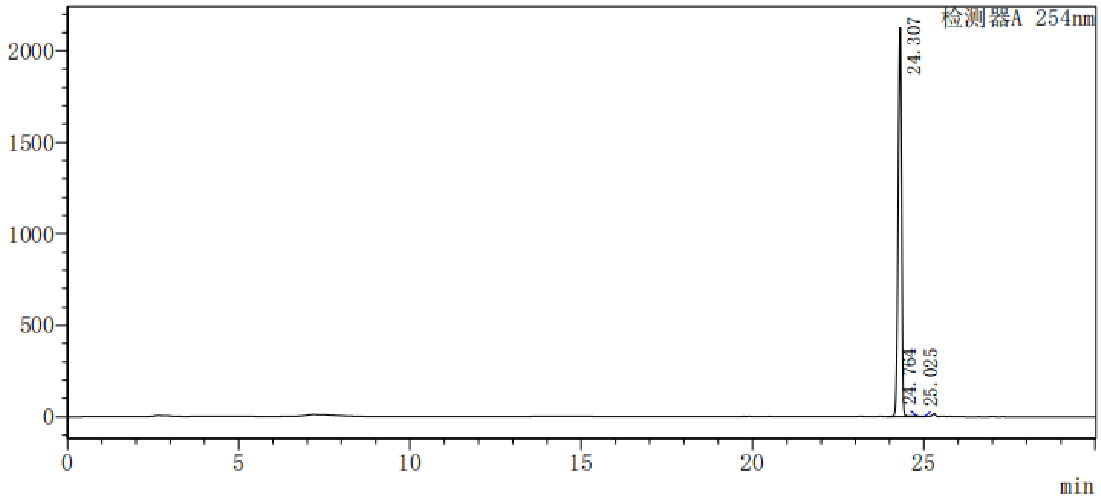

Detector A 254 nm

Peaks Time Area% Area Height

峰号	保留时间	面积%	面积	高度
1	24.307	99.368	14354539	2123978
2	24.764	0.552	79685	7865
3	25.025	0.080	11541	1989
总计		100.000	14445764	2133832

L15

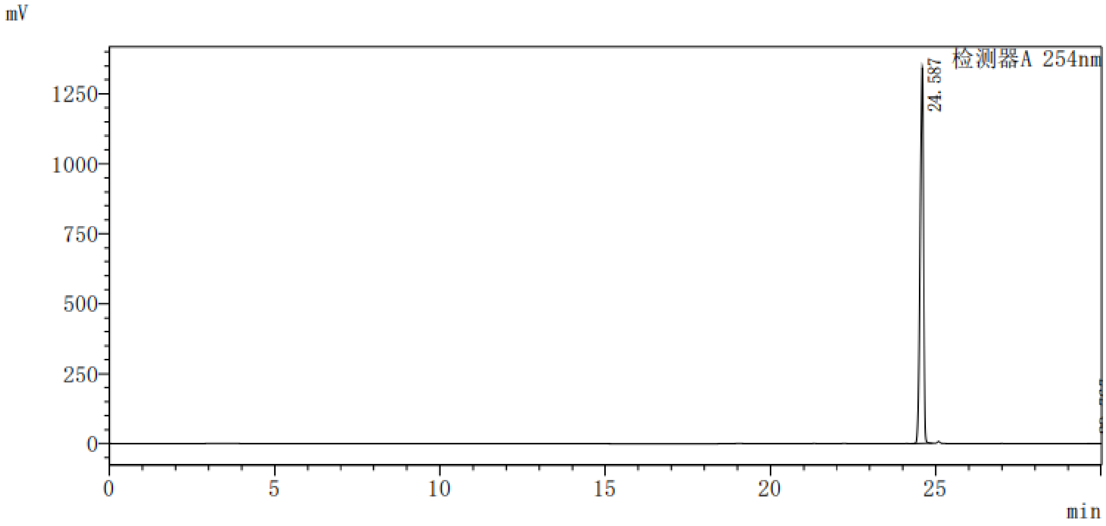

Detector A 254 nm

Peaks Time Area% Area Height

峰号	保留时间	面积%	面积	高度
1	24.587	99.975	9946250	1341675
2	29.767	0.025	2442	238
总计		100.000	9948692	1341913

Supplementary figure legends

Fig. S1. C1 has no effect on the mRNA level of eEF2K. Effects of **C1** treatment on the eEF2K mRNA levels in MDA-MB-231 and HCC1806 cells. Results are expressed as mean ± SEM. n.s., not significant.

Fig. S2. Synthesis of compound C1-Biotin. Reagents and conditions: (i) Et_3N, DCM, *N*-(prop-2-yn-1-yl)piperidine-3-carboxamide, 0 °C to rt, 60%; (ii) H_2O, CuI, acetonitrile, sodium ascorbate, N_2, 62%.

Fig. S1

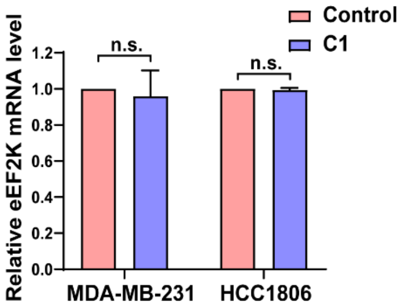

Control
C1
Relative eEF2K mRNA level
n.s.
n.s.
1.2
1.0
0.8
0.6
0.4
0.2
0.0
MDA-MB-231
HCC1806

Fig. S2

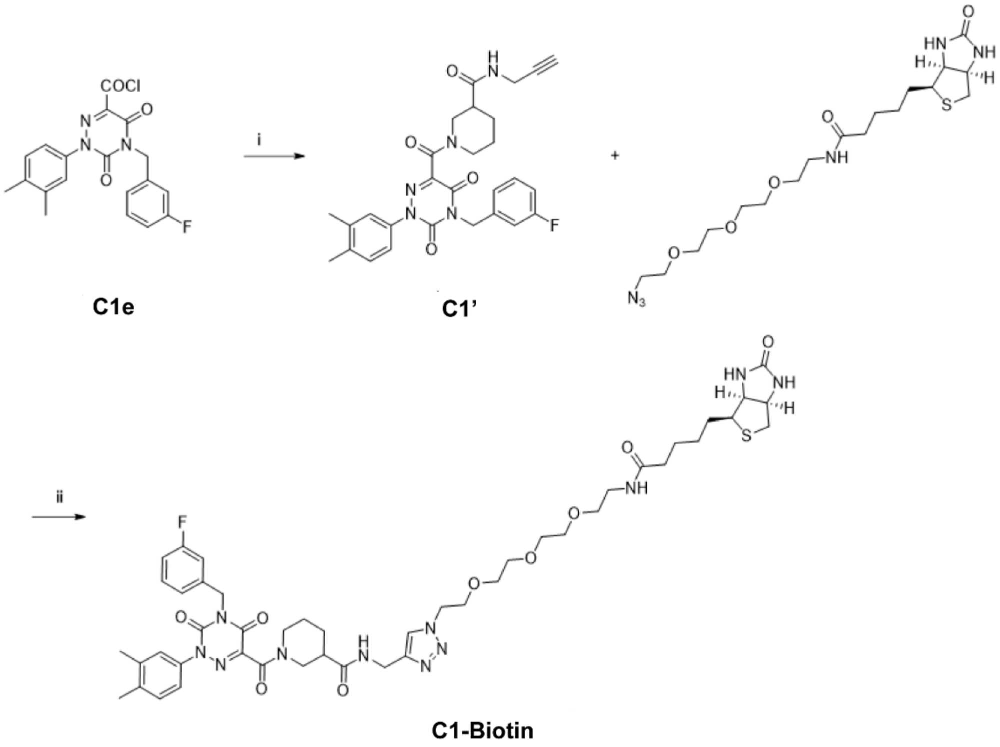

Table S1: The structures and anti-tumor activities of A-484954 derivatives against MDA-MB-231 cells.

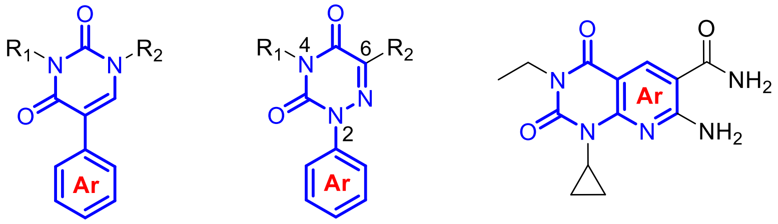

Series I　　Series II　　A-484954

No.	Structure	R_1	Ar	R_2	Inhibition (%) @ 20 μM	IC_{50} (nM)
**D2**	**I**	Bn	Py	*c*-Pr	10.48%	-
**D3**		Bn	Py	Bn	-20.90%	-
**D4**		Bn	Ph		2.52%	-
**I1**	**II**	Et	Ph	CN	16.83%	-
**I2**		Bn	3-Me-Ph		34.94%	-
**I3**		Bn	4-Me-Ph		65.12%	-
**I4**		Bn	3,4-di-Me-Ph		96.44%	950.6
**I5**		Me	3,4-di-Me-Ph		8.33%	-
**I6**		Pr	3,4-di-Me-Ph		36.57%	-
**I7**		Bn	3,4-di-Me-Ph	$CONH_2$	-10.91%	-
**J4**		Bn	3,4-di-Me-Ph	COOH	-13.98%	-
**L1**		Bn	3,4-di-Me-Ph	COOEt	-16.52%	-
**L2**		Bn	3,4-di-Me-Ph	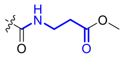	23.38%	--
**L3**		Bn	3,4-di-Me-Ph	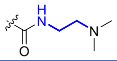	97.71%	> 1000
**L4**		Bn	3,4-di-Me-Ph	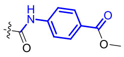	11.83%	-
**L5**		Bn	3,4-di-Me-Ph	$CON(Me)_2$	89.81%	866.9
**L6**		Bn	3,4-di-Me-Ph	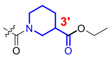	94.79%	110.9
**L7**		Bn	3,4-di-Me-Ph	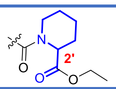	93.94%	836.1
**L8**		Bn	3,4-di-Me-Ph	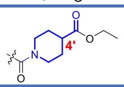	93.44%	939.9

**L9**		Bn	3,4-di-Me-Ph	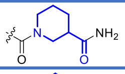	9.62%	-
**L10**		Bn	3,4-di-Me-Ph	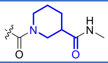	46.18%	-
**L11**		Bn	3,4-di-Me-Ph	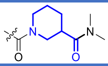	89.49%	> 1000
**L12**		Bn	Ph		28.40%	/
**L13**		Bn	3-Me-Ph		85.28%	>1000
**L14**		Bn	4-Me-Ph	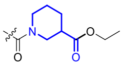	86.33%	683.3
**L15**		3-F-Bn	3,4-di-Me-Ph		98.44%	131.2
**L16**		4-F-Bn	3,4-di-Me-Ph		99.36%	> 1000
**I9**		3-F-Bn	3,4-di-Me-Ph	CN	91.06%	> 1000
**I10**		4-F-Bn	3,4-di-Me-Ph		93.58%	> 1000
**A-484954**					4.67%	-

Table S2: The kinetic parameters of 4 compounds and eEF2K protein analyzed by SPR.

Mobile phase	Stationary phase	Avg K_a (1/Ms)	Avg K_d (1/s)	Avg K_D (M)	Int.Intensity Level	ABS (tr_KD)
**I4**	eEF2K	1.65E+04	1.14E-02	6.92E-07	Strong	20.4634
**L5**	eEF2K	2.11E+02	1.01E-03	4.80E-06	Strong	17.6674
**L6**	eEF2K	1.69E+02	1.39E-04	8.22E-07	Strong	20.2151
**L15**	eEF2K	1.03E+05	1.30E-04	1.26E-09	Strong	29.5637
DMSO	eEF2K	1.15E+00	2.73E-01	2.38E-01	VW/None	2.0713
